# Position‐Dependent Stabilization of DNA/RNA Duplexes by Site‐Specific Incorporation of LNA Nucleosides

**DOI:** 10.1155/jna/7266073

**Published:** 2026-05-05

**Authors:** Elisa Tomita-Sudo, Tomoka Akita, Nae Sakimoto, Saori Tahara-Takamine, Renshin Sano, Shigenori Iwai, Junji Kawakami

**Affiliations:** ^1^ Konan Laboratory for Oligonucleotide Therapeutics (KOLOT), Konan University, Kobe, Hyogo, Japan, konan-u.ac.jp; ^2^ Frontiers of Innovative Research in Science and Technology (FIRST), Konan University, Kobe, Hyogo, Japan, konan-u.ac.jp

## Abstract

Owing to their enhanced functional properties, 2 ^′^,4 ^′^‐bridged nucleic acids/locked nucleic acids (2 ^′^,4 ^′^‐BNAs/LNAs) are considered promising candidates for antisense oligonucleotide therapeutics. The spatial arrangement of LNA residues within the oligonucleotide sequence is a key determinant of binding affinity to the target RNA. In this study, a series of antisense oligonucleotides containing LNA at different positions was synthesized, and their binding affinities toward the target RNA were systematically evaluated. Oligonucleotides with LNA modifications at the terminal regions exhibited approximately 100‐fold lower affinity than those modified near the central region. These results offer valuable insights for the rational design of LNA‐based antisense oligonucleotides.

## 1. Introduction

Numerous chemically modified nucleic acids have been developed to enhance the resistance and effectiveness of antisense oligonucleotides (ASOs) [1–3]. Among these, 2 ^′^,4 ^′^‐bridged nucleic acid/locked nucleic acid (2 ^′^,4 ^′^‐BNA/LNA) (hereafter referred to as LNA), characterized by a conformationally restricted sugar moiety, significantly improves intracellular stability and exhibits substantially increased affinity for complementary RNA strands [4, 5]. Since the formation of a stable duplex with the target RNA is essential for ASO function, LNA‐containing ASOs are considered highly promising candidates for therapeutic applications.

A key factor in the design of effective LNA‐modified ASOs is the optimization of binding affinity to the target RNA. Insufficient affinity typically results in poor therapeutic performance, while excessively strong binding can also compromise the desired biological activity [6, 7]. For example, in RNase H–dependent ASOs, efficient gene silencing requires multiple cycles of RNase H–mediated cleavage; however, overly stable DNA/RNA duplexes can hinder enzyme turnover, thereby reducing silencing efficiency [6]. Similarly, splice‐switching ASOs (SSOs) with excessively high RNA affinity have been reported to exhibit reduced exon‐skipping activity, likely due to intra‐ or intermolecular interactions that decrease the concentration of functionally available SSOs [7]. Therefore, achieving an appropriate balance in RNA binding affinity is critical for the successful design of ASOs, regardless of their mechanism of action.

Several factors influence the duplex‐stabilizing effect of incorporating LNA or other chemically modified nucleotides, including the number of LNA modifications [8–12], the identity of the substituted base (A, G, C, T, or U) [8, 10–12], and the nature of the nearest‐neighbor base pairs [10, 13–16]. Among these, the number of incorporated LNA residues is generally the most critical determinant of duplex stability, with higher numbers typically producing greater stabilizing effects. Regarding base identity, previous studies have indicated that LNA substitution at pyrimidine positions may confer a stronger stabilizing effect than at purine positions [13]. Additionally, the stabilizing influence of LNA is modulated by nearest‐neighbor interactions. However, the magnitude of variation attributable to base type or flanking sequence context is generally minor compared with that arising from the number of modifications.

Therefore, the total number of modified nucleotides is the primary parameter for achieving the desired RNA‐binding affinity in ASO design. This principle is exemplified by the design of approved exon‐skipping ASOs, which act through steric hindrance and therefore impose no positional or numerical restrictions on modifications. For example, all nucleotides in approved drugs such as nusinersen are fully modified with 2 ^′^‐O‐methoxyethyl (2 ^′^‐MOE), whereas eteplirsen, golodirsen, viltolarsen, and casimersen employ morpholino backbones. Similarly, RNase H–dependent ASOs typically adopt a 5‐10‐5 gapmer configuration, in which the terminal five nucleotides at both ends are modified with 2 ^′^‐MOE. This structural motif is consistently observed in clinically approved agents such as mipomersen, inotersen, volanesorsen, tofersen, and eplontersen.

However, owing to the pronounced duplex‐stabilizing effect of LNA, SSOs in which all nucleotides are LNA‐modified exhibit reduced exon‐skipping activity despite their high RNA‐binding affinity [7]. A similar effect is observed in RNase H–dependent ASOs, where incorporating more than three LNA residues at each terminus impairs enzyme turnover, thereby diminishing gene‐silencing efficiency [6]. These observations highlight the need to carefully optimize the number of LNA modifications in ASO design. An important question arises: If the number of LNA modifications is fixed, does their stabilizing effect remain constant regardless of their position within the oligonucleotide? Are there specific positional arrangements of LNA incorporation that result in enhanced affinity for the target RNA? Addressing these questions requires consideration of the positional distribution of LNA residues, as their contribution to duplex stability depends not only on quantity but also on location. However, few studies have systematically evaluated the positional effects of LNA incorporation on DNA/RNA duplex affinity [11]. Furthermore, existing reports often vary in both the number and sequence context of LNA‐modified oligonucleotides, limiting the ability to directly compare positional effects. In this study, the thermodynamic stability of DNA/RNA duplexes composed of LNA‐modified DNA strands and complementary RNA targets (mimicking mRNA) was investigated. Thymine was selected as the LNA base moiety to facilitate direct comparison with our previous study, in which a single LNA‐T modification was introduced at the central region of ASOs. Binding affinity was determined using equilibrium binding constants (*K*) and standard Gibbs free energies at 37°C ($\Delta G_{37}^{{}^{\circ}}$), which provide more reliable and concentration‐independent measures of duplex stability than melting temperature (*T*_m_) values.

UV melting curves were obtained for ASO/target RNA duplexes containing a single LNA‐modified thymidine at various positions within a model sequence. From these curves, thermodynamic parameters—including $\Delta G_{37}^{{}^{\circ}}$, enthalpy change (*Δ* *H* ^°^), and entropy change (*Δ* *S* ^°^)—were derived and compared to assess the influence of LNA position on duplex stability. The results demonstrated a clear positional dependence of the duplex‐stabilizing effects associated with LNA incorporation.

## 2. Materials and Methods

### 2.1. Oligonucleotides

Oligonucleotides were obtained from Gene Design, Inc. (Osaka, Japan). Antisense DNAs—designated i(DNA), ii(DNA), iii(DNA), and iv(DNA)—were synthesized using LNA‐type thymidine phosphoramidite. The specific sequences are provided in Table 1. In a previous study, all unmodified DNA/RNA duplexes used in this study were confirmed to undergo a two‐state transition, validating their suitability for thermodynamic analysis [14]. To investigate the positional effects of LNA incorporation, individual thymidine residues were systematically substituted with LNA, as detailed in Table 1. Numbers in parentheses following the oligonucleotide names indicate the position of LNA substitution; for example, i(3) denotes LNA substitution at the third thymidine position. Positional values in the table are expressed relative to the strand termini, with positive (+) and negative (−) values representing positions from the 5 ^′^ and 3 ^′^ ends, respectively. For instance, position −5 refers to the fifth nucleotide from the 3 ^′^ end.

**Table 1:** Sequences used in this study.

Oligo name	Sequence	Positional group
i(RNA)	3 ^′^‐AAGUAGUAGUAA‐5 ^′^	None
i(DNA)	5 ^′^‐TTCATCATCATT‐3 ^′^	None
i(1)	5 ^′^′‐T(L)TACTACTACTT‐3 ^′^	+1
i(2)	5 ^′^‐TT(L)CATCATCATT‐3 ^′^	+2
i(3)	5 ^′^‐TTCAT(L)CATCATT‐3 ^′^	+5
i(4)	5 ^′^‐TTCATCAT(L)CATT‐3 ^′^	−5
i(5)	5 ^′^‐TTCATCATCAT(L)T‐3 ^′^	−2
i(6)	5 ^′^‐TTCATCATCATT(L)‐3 ^′^	−1
ii(RNA)	3 ^′^‐AACUACUACUAA‐5 ^′^	None
ii(DNA)	5 ^′^‐TTGATGATGATT‐3 ^′^	None
ii(1)	5 ^′^‐T(L)TGATGATGATT‐3 ^′^	+1
ii(2)	5 ^′^‐TT(L)GATGATGATT‐3 ^′^	+2
ii(3)	5 ^′^‐TTGAT(L)GATGATT‐3 ^′^	+5
ii(4)	5 ^′^‐TTGATGAT(L)GATT‐3 ^′^	−5
ii(5)	5 ^′^‐TTGATGATGAT(L)T‐3 ^′^	−2
ii(6)	5 ^′^‐TTGATGATGATT(L)‐3 ^′^	−1
iii(RNA)	3 ^′^‐AGCAAGCAAGCA‐5 ^′^	None
iii(DNA)	5 ^′^‐TCGTTCGTTCGT‐3 ^′^	None
iii(1)	5 ^′^‐T(L)CGTTCGTTCGT‐3 ^′^	+1
iii(2)	5 ^′^‐TCGT(L)TCGTTCGT‐3 ^′^	+4
iii(3)	5 ^′^‐TCGTT(L)CGTTCGT‐3 ^′^	+5
iii(4)	5 ^′^‐TCGTTCGT(L)TCGT‐3 ^′^	−5
iii(5)	5 ^′^‐TCGTTCGTT(L)CGT‐3 ^′^	−4
iii(6)	5 ^′^‐TCGTTCGTTCGT(L)‐3 ^′^	−1
iv(RNA)	3 ^′^‐AUGAAUGAAUGA‐5 ^′^	None
iv(DNA)	5 ^′^‐TACTTACTTACT‐3 ^′^	None
iv(1)	5 ^′^‐T(L)ACTTACTTACT‐3 ^′^	+1
vi(2)	5 ^′^‐TACT(L)TACTTACT‐3 ^′^	+4
iv(3)	5 ^′^‐TACTT(L)ACTTACT‐3 ^′^	+5
iv(4)	5 ^′^‐TACTTACT(L)TACT‐3 ^′^	−5
iv(5)	5 ^′^‐TACTTACTT(L)ACT‐3 ^′^	−4
iv(6)	5 ^′^‐TACTTACTTACT(L)‐3 ^′^	−1

*Note:* LNA monomers are shown as T(L).

All sample solutions contained 10 mM phosphate buffer (pH 7.0), 1 mM EDTA, and 100 mM NaCl. Antisense DNA and complementary RNA strands were mixed at a 1:1 molar ratio, with final concentrations specified in Figures S1–S5. Duplex formation was achieved by heating the solutions to 95°C, followed by gradual cooling to 10°C at a rate of 0.5°C/min prior to measurement.

### 2.2. Melting Experiments

UV melting experiments were conducted to monitor temperature‐dependent changes in absorbance at 260 nm using a UV‐1800 spectrophotometer equipped with a TMSPC‐8 temperature controller (Shimadzu, Kyoto, Japan). Measurements were performed in quartz cuvettes with path lengths of 1 and 0.1 cm. To prevent sample evaporation, pressure‐bonded adhesive seals were applied. Melting curves were obtained by heating the samples at a rate of 0.5°C/min. Thermodynamic parameters, including $\Delta G_{37}^{{}^{\circ}}$, *Δ* *H* ^°^, *Δ* *S* ^°^, and *T*_m_, were determined by curve fitting according to Equation (1), where *R* is the universal gas constant. This analytical procedure followed the method described in detail in Reference [17], hereafter referred to as the curve fitting method. The melting curves and the results of the curve fitting are shown in Figure S1. The experiments were performed at least in duplicate for each sample at various concentrations. Under the assumption that both *Δ* *H* ^°^ and *Δ* *S* ^°^ are independent of concentration, the average values and standard deviations (SDs) of *Δ* *H* ^°^, *Δ* *S* ^°^, and the derived $\Delta G_{37}^{{}^{\circ}}$ obtained from all curves at all concentration points were calculated, and the SDs were used as error values.

(1) lnK=−ΔH°RT+ΔS°R.

Additionally, the relationship between *T*_m_ and the total strand concentration (*C*_t_) was analyzed using Equation (2), based on the van′t Hoff plot method. Least‐squares fitting was performed using the analytical functions in Microsoft Excel. From the fitting results, *Δ* *H* ^°^, *Δ* *S* ^°^, and their standard errors were obtained, and $\Delta G_{37}^{{}^{\circ}}$ was subsequently calculated (see Figures S2–S5). This approach is hereafter referred to as the van′t Hoff method.

(2) 1Tm=ln Ct4×RΔH°+ΔS°ΔH°.

For evaluation, the average values of the thermodynamic parameters obtained from both the curve fitting and van′t Hoff methods were used (Table 2).

**Table 2:** Averaged parameters obtained by curve fittings and van′t Hoff plot.

	$\Delta G_{37}^{{}^{\circ}}$	*Δ* *H* ^°^	Δ*S*°	−*T* *Δ* *S* ^°^^a^	*T*_*m*_^b^	$\Delta \Delta G_{37}^{{}^{\circ}}$	*Δ* *Δ* *H* ^°^	−*T* *Δ* *Δ* *S* ^°^	*Δ* *T*_*m*_^b^
	kcal·mol^−1^	kcal·mol^−1^	cal·mol^−1^·K^−1^	kcal·mol^−1^	°C	kcal·mol^−1^	kcal·mol^−1^	kcal·mol^−1^	°C
i(DNA/RNA)^c^	−8.5	−85.2	−247.5	76.8	41.4				
i(1/RNA)	−8.9	−87.3	−252.8	78.4	43.0	−0.4	−2.1	1.6	1.6
i(2/RNA)	−9.8	−89.6	−257.1	79.7	45.8	−1.4	−4.3	3.0	4.4
i(3/RNA)^c^	−9.7	−84.0	−239.6	74.3	46.4	−1.2	1.2	−2.4	4.9
i(4/RNA)^c^	−9.6	−84.1	−240.2	74.5	46.1	−1.1	1.2	−2.3	4.6
i(5/RNA)	−8.9	−89.5	−260.0	80.6	43.2	−0.4	−4.3	3.9	1.8
i(6/RNA)	−8.3	−80.8	−233.9	72.6	41.1	0.2	4.4	−4.2	−0.3
ii(DNA/RNA)^c^	−7.3	−77.7	−227.0	70.4	37.2				
ii(1/RNA)	−7.6	−81.1	−237.0	73.5	38.3	−0.3	−3.4	3.1	1.1
ii(2/RNA)	−8.7	−85.9	−248.9	77.2	42.1	−1.3	−8.1	6.8	4.9
ii(3/RNA)^c^	−8.9	−77.6	−221.5	68.7	43.3	−1.5	0.2	−1.7	6.1
ii(4/RNA)^c^	−8.8	−79.5	−228.2	70.8	42.8	−1.4	−1.8	0.4	5.6
ii(5/RNA)	−7.8	−78.2	−226.9	70.4	39.1	−0.5	−0.4	0.0	1.9
ii(6/RNA)	−7.2	−74.1	−215.6	66.9	36.7	0.2	3.7	−3.5	−0.6
iii(DNA/RNA)^c^	−12.1	−89.9	−250.8	77.8	54.7				
iii(1/RNA)	−12.8	−97.6	−273.4	84.8	55.5	−0.7	−7.7	7.0	0.9
iii(2/RNA)^c^	−13.5	−91.8	−252.4	78.3	59.3	−1.3	−1.9	0.5	4.7
iii(3/RNA)^c^	−14.1	−96.7	−266.4	82.6	60.4	−1.9	−6.8	4.8	5.7
iii(4/RNA)^c^	−13.3	−91.8	−253.2	78.5	58.6	−1.2	−1.9	0.8	3.9
iii(5/RNA)^c^	−13.8	−94.3	−259.6	80.5	60.0	−1.7	−4.4	2.8	5.3
iii(6/RNA)	−12.5	−97.0	−272.3	84.4	54.7	−0.4	−7.0	6.7	0.0
iv(DNA/RNA)^c^	−8.4	−88.6	−258.7	80.2	40.7				
iv(1/RNA)	−9.1	−101.3	−297.3	92.2	42.6	−0.7	−12.7	12.0	1.9
iv(2/RNA)^c^	−10.0	−92.5	−266.1	82.5	46.3	−1.6	−3.9	2.3	5.5
iv(3/RNA)^c^	−10.5	−95.1	−272.7	84.6	47.6	−2.1	−6.4	4.4	6.9
iv(4/RNA)^c^	−9.8	−91.6	−263.6	81.8	45.7	−1.4	−2.9	1.5	4.9
iv(5/RNA)^c^	−10.2	−91.0	−260.5	80.8	47.2	−1.8	−2.4	0.5	6.4
iv(6/RNA)	−8.5	−93.8	−275.1	85.3	41.0	−0.1	−5.2	5.1	0.3

^a^Calculated at 37°C.

^b^Obtained by the curve fitting at *C*_*t*_ = 30 *μ*M.

^c^From our previous study [14].

### 2.3. Estimation of the Stabilization Effect of LNA

The differences in thermodynamic parameters—$\Delta \Delta G_{37}^{{}^{\circ}}$, *Δ* *Δ* *H* ^°^, and *Δ* *Δ* *S* ^°^—were calculated as shown in Table 2, using the following equations:

(3) ΔΔG37°=ΔG37°LNA-containing DNA/RNA−ΔG37°DNA/RNA,

(4) ΔΔH°=ΔH°LNA-containing DNA/RNA−ΔH°DNA/RNA,

(5) ΔΔS°=ΔS°LNA-containing DNA/RNA−ΔS°DNA/RNA.

The duplex‐stabilizing effect of LNA incorporation was assessed using $\Delta \Delta G_{37}^{{}^{\circ}}$, where a negative value indicates enhanced thermodynamic stability of the DNA/RNA duplex.

To express the stabilizing effect in terms of the change in binding constant, the following derivation was applied. Starting with the standard thermodynamic relation $\Delta G_{37}^{{}^{\circ}}=-RT\text{ln}K$, Equation (3) can be rewritten as

(6) ΔΔG37°=−RTlnKLNA-containing DNA/RNA−−RTlnKDNA/RNA ,

which simplifies to

(7) ΔΔG37°=−RTlnKLNA-containing DNA/RNA/KDNA/RNA.

Next, by assigning the following values in Equation (7), *R* ≈ 0.00199 kcal∙mol^−1^∙K^−1^, *T* at 37^°^C = 310.15 K, and ln*X* ≈ 2.303 log_10_ *X*, and substituting numerical values for the constants, the following expressions are obtained:

(8) ΔΔG37°≈1.4 log10KLNA-containing DNA/RNA/KDNA/RNA,

(9) KLNA-containing DNA/RNA/KDNA/RNA≈10ΔΔG37°/1.4.

For instance, if $\Delta \Delta G_{37}^{{}^{\circ}}=-2.8$ kcal⋅mol^−1^, the corresponding increase in the binding constant is approximately 100‐fold, as calculated from Equation (9).

## 3. Results

### 3.1. Measurement of Melting Curves and Determination of Thermodynamic Parameters

Four antisense DNA sequences were designed to investigate the positional effects of LNA incorporation (Table 1). Among these, i(DNA) and ii(DNA) had similar sequences, each containing two terminal thymidines (TT) at both the 5 ^′^ and 3 ^′^ ends. Thymidines were located at the first, second, fifth, eighth, eleventh, and twelfth positions from the 5 ^′^ end. The third sequence, designated iii(DNA), contained the highest proportion of guanine and cytosine residues. In iii(DNA) and iv(DNA), thymidines were positioned at the first, fourth, fifth, eighth, ninth, and twelfth positions from the 5 ^′^ end.

Melting curves for all duplex samples were obtained by monitoring UV absorbance. Pressure‐bonded adhesive seals were applied to prevent sample evaporation, thereby ensuring that elevated temperatures did not compromise the accuracy of melting curve analyses. Thermodynamic parameters were determined using both the curve fitting method (Figure S1) and the van′t Hoff plot method (Figures S2–S5).

### 3.2. Comparison of Thermodynamic Parameters Obtained From Melting Experiments

The thermodynamic parameters determined by both analytical methods—curve fitting and van′t Hoff plots—are summarized in Table 2. To determine the reliability of the results, the associated errors were first evaluated. The maximum errors for $\Delta G_{37}^{{}^{\circ}}$, *Δ* *H* ^°^, and −*T* *Δ* *S* ^°^ obtained from curve fitting were 0.2, 5.2, and 5.0 kcal∙mol^−1^, respectively, whereas those from van′t Hoff analysis were 0.9, 7.1, and 6.3 kcal·mol^−1^. In all cases, the errors were within 10% of the corresponding mean values for each sequence, confirming the accuracy of the measurements.

Comparison of the thermodynamic values obtained by the two methods showed differences ranging from 0.0 to 0.3 kcal·mol^−1^ for $\Delta G_{37}^{{}^{\circ}}$, 0.6 to 20.9 kcal·mol^−1^ for *Δ* *H* ^°^, and 0.6 to 20.6 kcal·mol^−1^ for −*T* *Δ* *S* ^°^. The largest discrepancies were observed for i(6)/RNA and iv(1)/RNA, both containing LNA modifications at terminal positions. Even in these cases, the differences between the two methods did not exceed 15%. For the remaining sequences, the parameters obtained by both methods were in good agreement, supporting the overall reliability and consistency of the thermodynamic data.

### 3.3. Dependence of the Duplex‐Stabilizing Effect on LNA Position

The average thermodynamic parameter values obtained from both analytical methods were used for comparison (Table 2). All four unmodified DNA/RNA duplexes consisted of 12 base pairs. Among these, the iii(DNA)/RNA duplex had the highest GC content (50%, with 6 of 12 bases) and exhibited a $\Delta G_{37}^{{}^{\circ}}$ approximately 4 kcal∙mol^−1^ more negative than those of the other unmodified duplexes, indicating greater thermodynamic stability. Similarly, LNA‐modified duplexes derived from Sequence iii demonstrated higher stability than those based on the other sequences.

**Figure 1:**
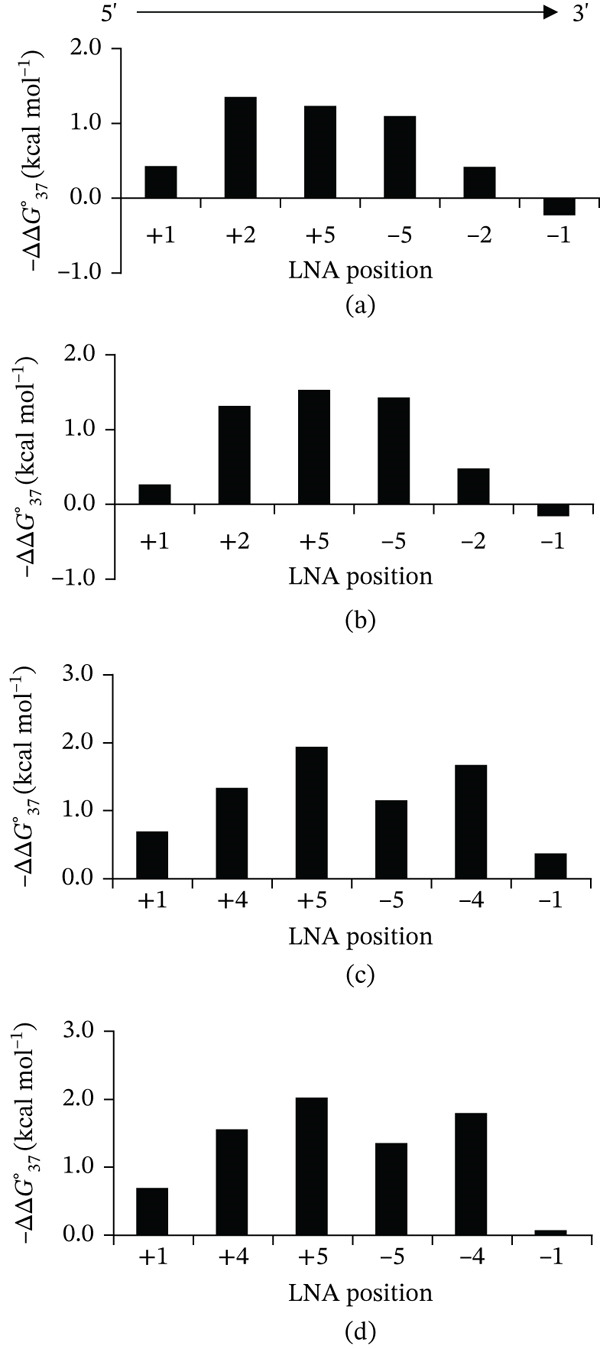
$\Delta \Delta G_{37}^{{}^{\circ}}$ values for duplexes with Sequences i–iv.

**Figure 2:**
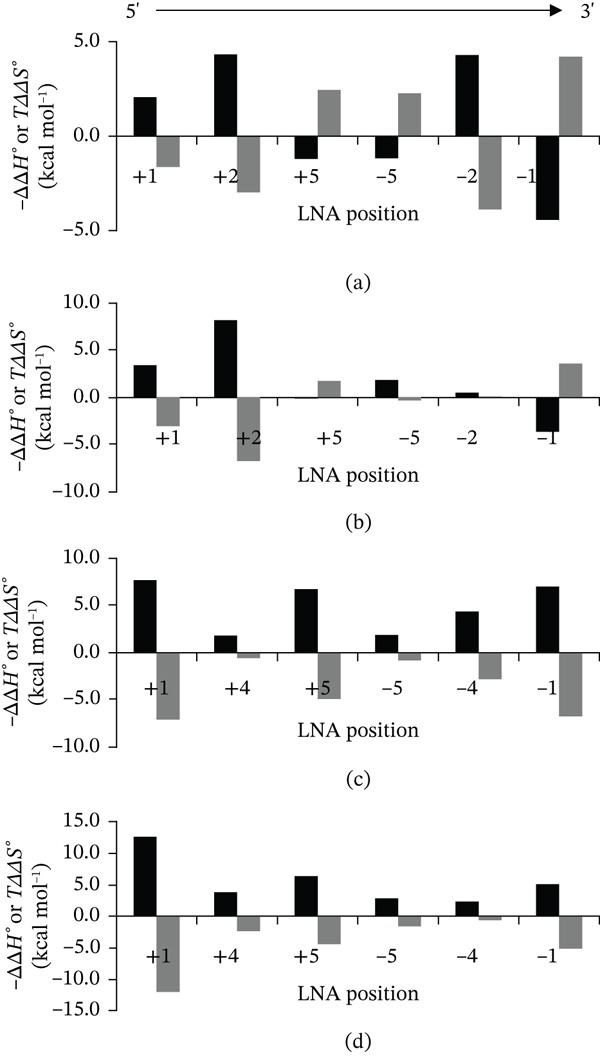
*Δ* *Δ* *H* ^°^ and −*T* *Δ* *Δ* *S* ^°^ values for duplexes with Sequences i–iv. Black bars represent *Δ* *Δ* *H* ^°^, and gray bars represent −*T* *Δ* *Δ* *S* ^°^.

Thermodynamic parameters of unmodified and LNA‐modified DNA/RNA duplexes were compared (Figure 1). The $\Delta \Delta G_{37}^{{}^{\circ}}$ values ranged from −2.1 to −0.2 kcal∙mol^−1^. Notably, in certain cases—specifically i(6)/RNA and ii(6)/RNA—positive $\Delta \Delta G_{37}^{{}^{\circ}}$ values were observed, indicating that LNA incorporation at these positions led to duplex destabilization rather than stabilization. Comparisons of *Δ* *Δ* *H* ^°^ and −*T* *Δ* *Δ* *S* ^°^ (Figure 2) showed that most LNA‐modified duplexes were stabilized enthalpically.

In contrast, several duplexes—including i(3/RNA), i(4/RNA), i(6/RNA), ii(3/RNA), and ii(6)/RNA—exhibited entropy‐driven stabilization. While *Δ* *Δ* *H* ^°^ and −*T* *Δ* *Δ* *S* ^°^ did not show a strong positional dependence as $\Delta \Delta G_{37}^{{}^{\circ}}$, their absolute values were generally larger when LNA was incorporated at terminal positions compared with those introduced near the center of the sequence.

## 4. Discussion

LNA modifications enhance both nuclease resistance and binding affinity, making them promising candidates for incorporation into ASOs. However, in RNase H–dependent ASOs, the positional distribution of LNA is constrained by structural requirements. A continuous natural DNA/RNA hybrid region of at least six base pairs—and preferably eight—is necessary for efficient RNase H recognition and cleavage [6]. Consequently, when modified nucleotides are introduced at both termini to improve exonuclease resistance, the central region available for LNA incorporation is limited, particularly in shorter ASOs, unless a sufficiently long gapmer structure is used [18–20]. In the case of SSOs, full LNA substitution across all nucleotides has been reported to reduce therapeutic effectiveness. Therefore, careful optimization of both the number and position of LNA modifications is essential for maximizing ASO biological activity [21–23]. In this study, the binding affinities of ASOs containing LNA at different positions were quantitatively evaluated against their target RNA, and the relationship between LNA position and potential therapeutic performance was systematically investigated.

**Figure 3:**
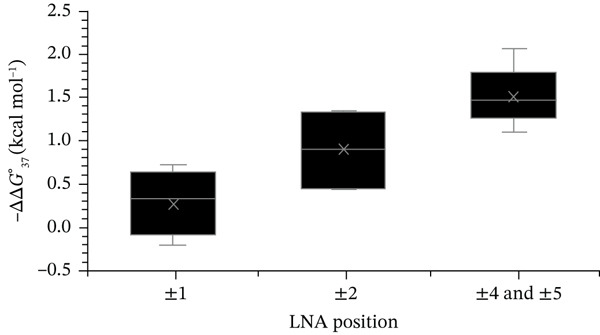
Positional dependence of LNA modification. The graph shows the average $\Delta \Delta G_{37}^{{}^{\circ}}$ values for all sequences classified into the ±1, ±2, and ±4/±5 groups, as defined in Table 1. Data for ±4 and ±5 are combined.

As shown in Figure 1, incorporation of LNA near the strand center produced a stabilization effect of approximately −1.5 kcal·mol^−1^. However, thermodynamic stability progressively decreased as the LNA position shifted toward the termini, with modifications at the 3 ^′^ end exhibiting minimal or no stabilizing effect. To clarify this positional relationship, all LNA‐containing duplexes were classified into groups based on substitution position, as defined in Table 1. Specifically, the ±1 group included LNAs at terminal positions, ±2 near the ends, and ±4/±5 near the strand center. The corresponding $-\Delta \Delta G_{37}^{{}^{\circ}}$ values were as follows: ±1 ranged from 0.7 to −0.2 kcal·mol^−1^, ±2 from 1.4 to 0.4 kcal·mol^−1^, and ±4/±5 from 2.1 to 1.1 kcal·mol^−1^ (Figure 3). These results indicate that the duplex‐stabilizing effect of a single LNA substitution increases in the order ±1 < ±2 < ±4/±5. In summary, LNA substitutions near the strand center confer greater thermodynamic stability than modifications located near or at the termini.

**Figure 4:**
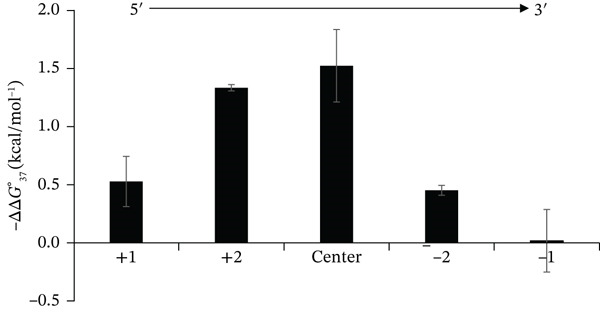
Positional dependence of LNA modification. The graph presents the average $\Delta \Delta G_{37}^{{}^{\circ}}$ values for the +1, +2, center (±4 and ±5), −2, and −1 groups. The stabilizing effect of LNA varies with its position, with central modifications exhibiting the highest enhancement of duplex stability.

The ±1 group was further subdivided to examine the positional dependence of LNA modifications at the termini. The $-\Delta \Delta G_{37}^{{}^{\circ}}$ values for the +1 and −1 subgroups ranged from 0.3 to 0.7 kcal∙mol^−1^ and −0.2 to 0.4 kcal∙mol^−1^, respectively, indicating that LNA incorporation at the 3 ^′^ end produced minimal duplex stabilization. A similar pattern was observed for the ±2 group: The +2 subgroup exhibited $-\Delta \Delta G_{37}^{{}^{\circ}}$ values of 1.3–1.4 kcal·mol^−1^, whereas the −2 subgroup ranged from 0.4 to 0.5 kcal·mol^−1^, again suggesting a relatively weaker stabilizing effect for 3 ^′^‐end modifications. The average $-\Delta \Delta G_{37}^{{}^{\circ}}$ values for each subgroup are summarized in Figure 4. These results clearly demonstrate that the stabilizing effect of LNA depends strongly on its position within the oligonucleotide. Notably, the difference in $\Delta \Delta G_{37}^{{}^{\circ}}$ between modifications at the center and those at the −1 position was approximately 1.5 kcal·mol^−1^. According to Equation (9), this corresponds to an approximate 10‐fold increase in binding affinity achieved simply by relocating the LNA modification from the terminus to the strand center. Though we did not investigate the effect of LNA modification at the ±3 position, we evaluated the binding properties of shortmers, potentially generated during ASO manufacturing, toward the therapeutic target RNA in another publication [24], which suggests that the stabilizing effect is likely to change gradually from the ends. A similar positional dependence has been reported for RNA/RNA duplexes, where the stabilizing effect increases as the modification site approaches the duplex center, although the magnitude of this effect varies depending on the type of modified nucleoside [25]. Unlike previous studies, the present work employed a constant sequence, incorporating LNA at different positions to isolate the effect of modification location. The results clearly demonstrate that the thermodynamic stability of DNA/RNA duplexes is significantly influenced by LNA position, providing valuable insights and practical guidance for the rational design of LNA‐containing ASOs.

The stabilizing effect of LNA modifications is strongly influenced by their position within the oligonucleotide strand. In this study, LNA incorporation at the center of the DNA strand produced a stabilization of approximately 1.5 kcal·mol^−1^, whereas modifications at the termini exhibited little to no stabilizing effect. One possible explanation is that the intrinsic stabilizing potential of LNA is fully realized only when the modification is positioned near the duplex center, while this effect is diminished or lost at the ends. Nucleotides in terminal regions exhibit greater structural flexibility and are more prone to transient dissociation from their complementary bases—a phenomenon known as fraying [26–30]. When LNA is placed at the duplex ends, the enhanced rigidity and stacking interactions typically conferred by the modification are likely disrupted by such dynamic fraying, thereby reducing its stabilizing effect. Specifically, the reduced stabilization observed for 3 ^′^‐end LNA modifications can be explained by the absence of an adjacent nucleotide necessary for structural support. As proposed by Petersen et al., the sugar moiety of the nucleotide immediately 3 ^′^ to an LNA‐modified nucleotide adopts an N‐type sugar puckering conformation, which contributes to overall duplex stabilization [31]. In the case of 3 ^′^‐terminal LNA modifications, this adjacent nucleotide is absent, potentially limiting conformational rigidity and thus diminishing the stabilizing effect relative to internal LNA substitutions.

Based on the thermodynamic data obtained in this study, the design of LNA‐modified ASO therapeutics can be reconsidered from a stability‐oriented perspective, though the functional or therapeutic performance of ASOs has not been evaluated. Incorporation of LNA at the termini provides minimal thermodynamic benefit. Therefore, in exon‐skipping ASO therapeutics, LNA modifications are more effectively placed in the central region of the strand rather than at the ends. In contrast, RNase H–dependent ASOs require an unmodified DNA/RNA hybrid region of approximately seven bases near the center to serve as a substrate for enzymatic cleavage. As a result, LNA modifications in this central region should be avoided. An optimal design in such cases would exclude LNA substitutions not only from the central seven bases but also from the first and second positions at both termini, where stabilization is limited. This strategy can guide the efficient placement of multiple LNA residues in ASOs while maintaining functional activity. The position‐dependent effects of single LNA modifications on duplex thermodynamic stability, as demonstrated in this study using LNA‐T, provide a valuable framework for the rational design of ASOs.

## 5. Conclusions

In this study, we determined the thermodynamic parameters for duplex formation between antisense oligodeoxyribonucleotides containing a single LNA at various positions and their complementary RNA strands. LNA incorporation in the central region produced the greatest duplex stabilization, whereas the stabilizing effect progressively decreased as the modification site approached the termini. In particular, LNA at the 3 ^′^ end showed little or no stabilizing effect. These findings indicate that the thermodynamic contribution of LNA is strongly position‐dependent and suggest that terminal positions, especially the first and second nucleotides from either end, are less favorable sites for LNA incorporation when duplex stabilization is a primary design objective.

## Author Contributions

Conceptualization: Junji Kawakami and Saori Tahara‐Takamine. Investigation: Elisa Tomita‐Sudo and Saori Tahara‐Takamine. Data curation: Elisa Tomita‐Sudo and Renshin Sano. Writing—original draft preparation: Elisa Tomita‐Sudo, Tomoka Akita, and Nae Sakimoto. Supervision: Shigenori Iwai and Junji Kawakami. Funding acquisition: Junji Kawakami.

## Funding

This study was funded by AMED, JP21ae0121022, JP21ae0121023, JP21ae0121024, and JP24mk0121278h0401.

## Disclosure

All authors have read and agreed to the published version of the manuscript.

## Conflicts of Interest

The authors declare no conflicts of interest.

## Supporting information


**Supporting Information** Additional supporting information can be found online in the Supporting Information section. The supporting information includes the data to obtain the thermodynamic parameters for each duplex. Figure S1: Melting curves and van′t Hoff plots of the duplexes. Figures S2–S5: Linear least squares fitting of van′t Hoff plots showing inverse melting point temperatures (1/*T*_m_) at different total oligonucleotide concentrations (*C*_t_) of the duplexes with Sequences i–iv.

## Data Availability

The data that supports the findings of this study are available in the supporting information of this article.
