# Supplementary material for: Position‐Dependent Stabilization of DNA/RNA Duplexes by Site‐Specific Incorporation of LNA Nucleosides
Source: J Nucleic Acids. 2026 May 5;2026:7266073. doi: 10.1155/jna/7266073 (PMC13139905; doi:10.1155/jna/7266073)
Supplement: Supplementary file 1 — Supporting Information Additional supporting information can be found online in the Supporting Information section. The supporting information includes the data to obtain the thermodynamic parameters for each duplex. Figure S1: Melting curves and van′t Hoff plots of the duplexes. Figures S2–S5: Linear least squares fitting of van′t Hoff plots showing inverse melting point temperatures (1/T m) at different total oligonucleotide concentrations (C t) of the duplexes with Sequences i–iv. [file JNA-2026-7266073-s001.pdf]

Supporting Information

Position-dependent stabilization of DNA/RNA duplexes by site-specific incorporation of LNA nucleosides

Elisa Tomita-Sudo[1], Tomoka Akita[2], Nae Sakimoto[1], Saori Tahara-Takamine[2], Renshin Sano[2], Shigenori Iwai[1] and Junji Kawakami[1, 2,]*

[1] Konan Laboratory for Oligonucleotide Therapeutics (KOLOT), Konan University, 7-1-20 Minatojima-minamimachi, Chuo-ku, Kobe 650-0047, Japan

[2] Faculty of Frontiers of Innovative Research in Science and Technology (FIRST), Konan University, 7-1-20 Minatojima-minamimachi, Chuo-ku, Kobe 650-0047, Japan

[*] Corresponding author. Tel.: +81-78-303-1359; Fax: +81-78-303-1495; Email: kawakami@konan-u.ac.jp

i(DNA/RNA) C_t = 30 μM

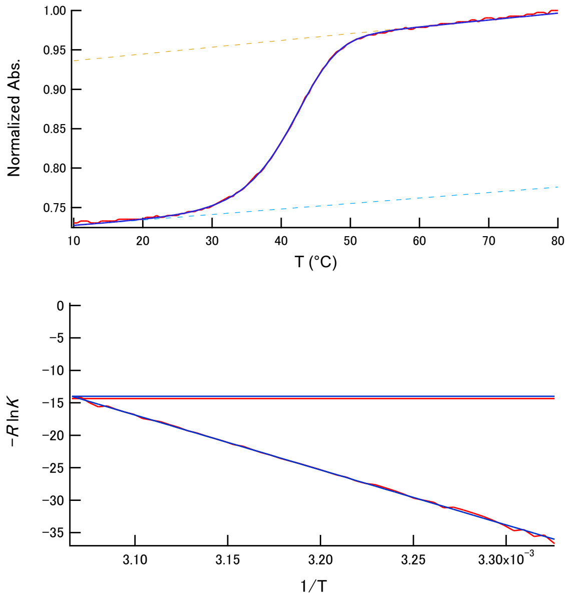

ΔG°_{37} = –8.48 kcal/mol

ΔH° = –84.28 kcal/mol

ΔS° = –244.38 cal/mol·K

T_m = 41.52°C

ΔG°_{37vh} = –8.50 kcal/mol

ΔH°_{vh} = –84.64 kcal/mol

ΔS°_{vh} = –245.51 cal/mol·K

$T_{m\ vh}$ = 41.55°C

i(DNA/RNA) C_t = 20 μM

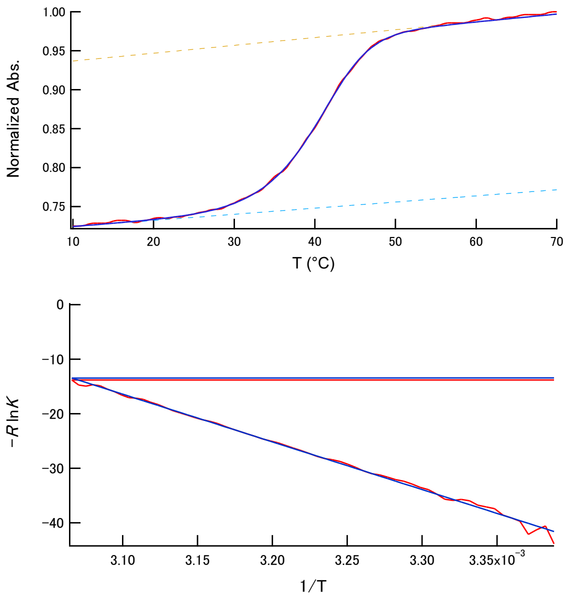

ΔG°_{37} = –8.43 kcal/mol

ΔH° = –86.21 kcal/mol

ΔS° = –250.76 cal/mol·K

T_m = 40.31°C

ΔG°_{37vh} = –8.46 kcal/mol

ΔH°_{vh} = –87.43 kcal/mol

ΔS°_{vh} = –254.61 cal/mol·K

$T_{m\ vh}$ = 40.37°C

i(DNA/RNA) C_t = 15 μM

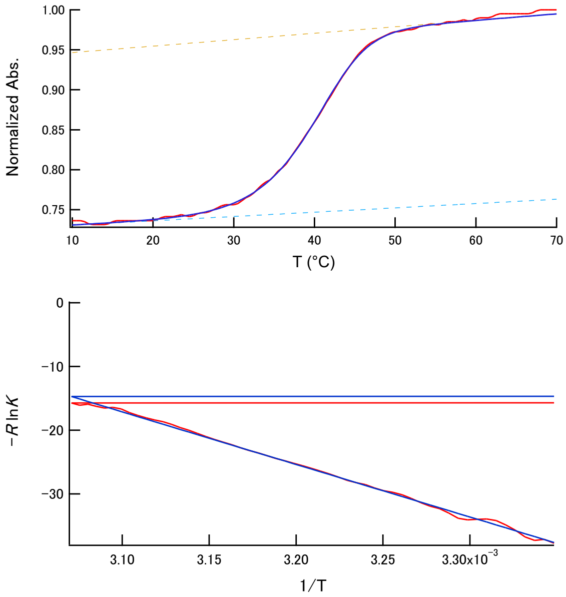

$\Delta G°_{37}$ = −8.48 kcal/mol

$\Delta H°$ = −83.29 kcal/mol

$\Delta S°$ = −241.22 cal/mol·K

T_m = 39.93°C

$\Delta G°_{37vh}$ = −8.49 kcal/mol

$\Delta H°_{vh}$ = −82.51 kcal/mol

$\Delta S°_{vh}$ = −238.64 cal/mol·K

$T_{m\ vh}$ = 40.00°C

i(DNA/RNA) C_t = 10 μM

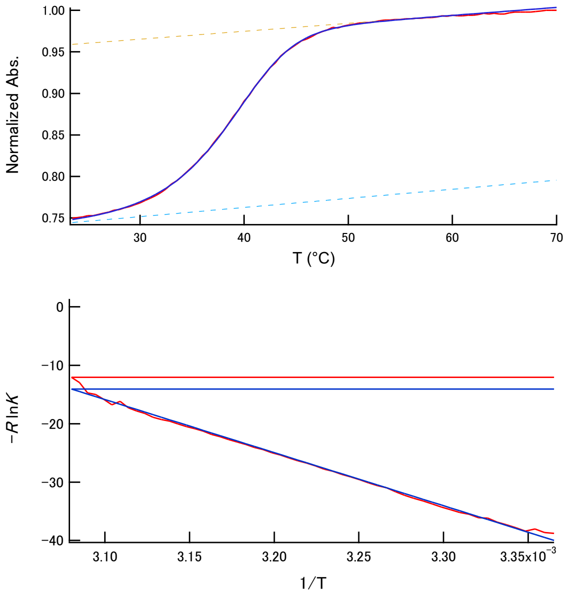

$\Delta G°_{37}$ = −8.45 kcal/mol

$\Delta H°$ = −90.05 kcal/mol

$\Delta S°$ = −263.10 cal/mol·K

T_m = 38.74°C

$\Delta G°_{37vh}$ = −8.42 kcal/mol

$\Delta H°_{vh}$ = −90.83 kcal/mol

$\Delta S°_{vh}$ = −265.72 cal/mol·K

$T_{m\ vh}$ = 38.60°C

i(DNA/RNA) C_t = 7.5 µM

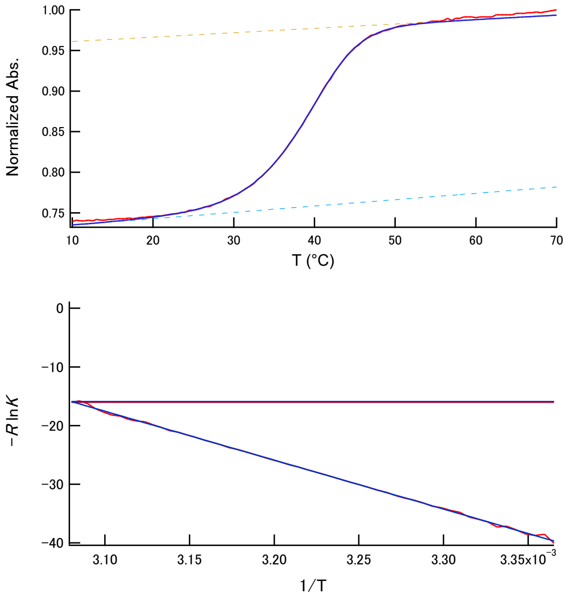

$\Delta G°_{37}$ = –8.66 kcal/mol

$\Delta H°$ = –83.21 kcal/mol

$\Delta S°$ = –240.36 cal/mol·K

T_m = 39.00°C

$\Delta G°_{37vh}$ = –8.66 kcal/mol

$\Delta H°_{vh}$ = –83.37 kcal/mol

$\Delta S°_{vh}$ = –240.89 cal/mol·K

$T_{m\ vh}$ = 38.99°C

i(DNA/RNA) C_t = 5 µM

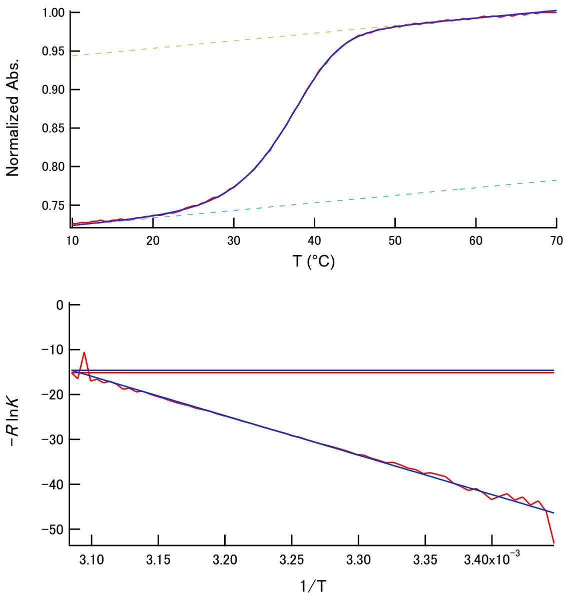

$\Delta G°_{37}$ = –8.33 kcal/mol

$\Delta H°$ = –85.09 kcal/mol

$\Delta S°$ = –247.50 cal/mol·K

T_m = 36.83°C

$\Delta G°_{37vh}$ = –8.32 kcal/mol

$\Delta H°_{vh}$ = –87.91 kcal/mol

$\Delta S°_{vh}$ = –256.62 cal/mol·K

$T_{m\ vh}$ = 36.81°C

i(DNA/RNA) C_t = 4.29 μM

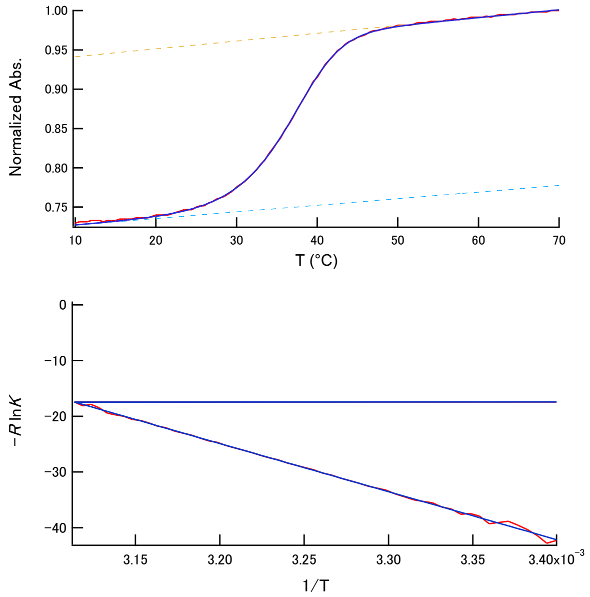

$\Delta G°_{37}$ = –8.37 kcal/mol

$\Delta H°$ = –86.11 kcal/mol

$\Delta S°$ = –250.66 cal/mol·K

T_m = 36.64°C

$\Delta G°_{37vh}$ = –8.37 kcal/mol

$\Delta H°_{vh}$ = –86.39 kcal/mol

$\Delta S°_{vh}$ = –251.54 cal/mol·K

$T_{m\ vh}$ = 36.65°C

i(DNA/RNA) C_t = 3.75 μM

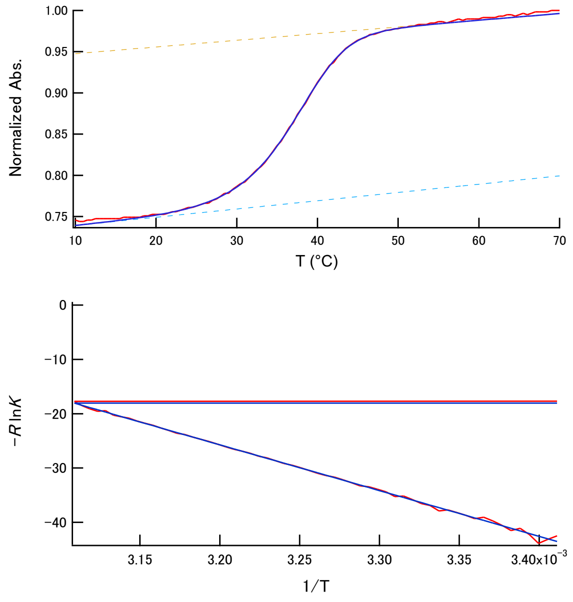

$\Delta G°_{37}$ = –8.61 kcal/mol

$\Delta H°$ = –84.16 kcal/mol

$\Delta S°$ = –243.57 cal/mol·K

T_m = 37.21°C

$\Delta G°_{37vh}$ = –8.61 kcal/mol

$\Delta H°_{vh}$ = –84.26 kcal/mol

$\Delta S°_{vh}$ = –243.92 cal/mol·K

$T_{m\ vh}$ = 37.21°C

i(1/RNA) C_t = 30 μM

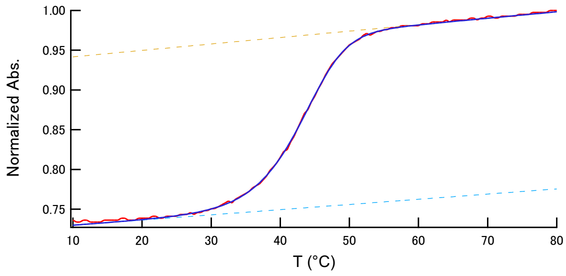

$\Delta G°_{37}$ = –8.95 kcal/mol

$\Delta H°$ = –87.86 kcal/mol

$\Delta S°$ = –254.44 cal/mol·K

T_m = 43.02°C

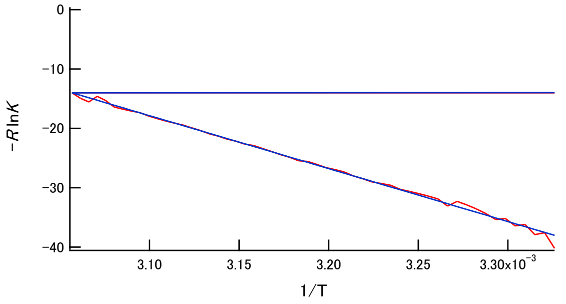

$\Delta G°_{37vh}$ = –8.98 kcal/mol

$\Delta H°_{vh}$ = –89.16 kcal/mol

$\Delta S°_{vh}$ = –258.52 cal/mol·K

$T_{m\ vh}$ = 43.04°C

i(1/RNA) C_t = 20 μM

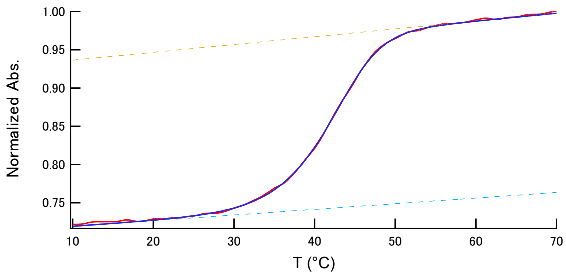

$\Delta G°_{37}$ = –8.94 kcal/mol

$\Delta H°$ = –89.72 kcal/mol

$\Delta S°$ = –260.48 cal/mol·K

T_m = 41.97°C

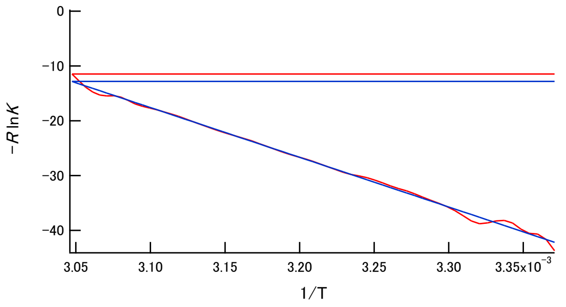

$\Delta G°_{37vh}$ = –8.96 kcal/mol

$\Delta H°_{vh}$ = –90.71 kcal/mol

$\Delta S°_{vh}$ = –263.60 cal/mol·K

$T_{m\ vh}$ = 41.98°C

i(1/RNA) C_t = 15 µM

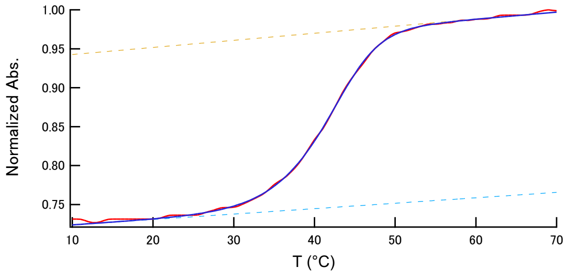

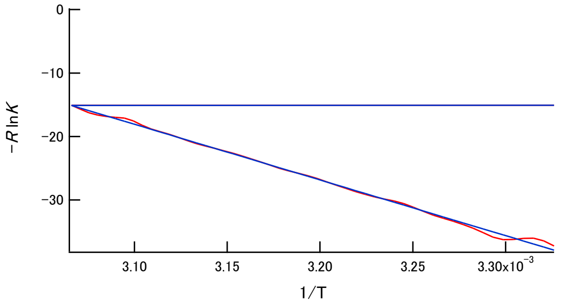

$\Delta G°_{37}$ = –8.99 kcal/mol

$\Delta H°$ = –88.41 kcal/mol

$\Delta S°$ = –256.04 cal/mol·K

T_m = 41.61°C

$\Delta G°_{37vh}$ = –8.99 kcal/mol

$\Delta H°_{vh}$ = –87.74 kcal/mol

$\Delta S°_{vh}$ = –253.92 cal/mol·K

$T_{m\ vh}$ = 41.61°C

i(1/RNA) C_t = 10 µM

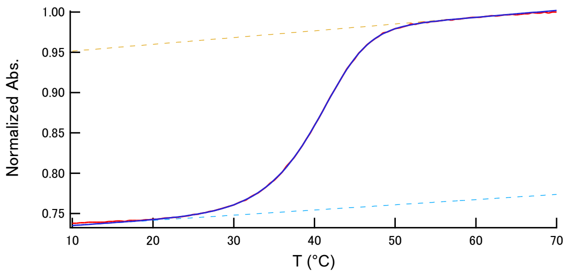

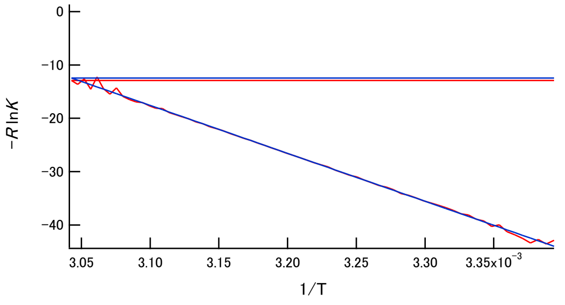

$\Delta G°_{37}$ = –8.92 kcal/mol

$\Delta H°$ = –89.47 kcal/mol

$\Delta S°$ = –259.73 cal/mol·K

T_m = 40.39°C

$\Delta G°_{37vh}$ = –8.92 kcal/mol

$\Delta H°_{vh}$ = –89.90 kcal/mol

$\Delta S°_{vh}$ = –261.10 cal/mol·K

$T_{m\ vh}$ = 40.37°C

i(1/RNA) C_t = 5 μM

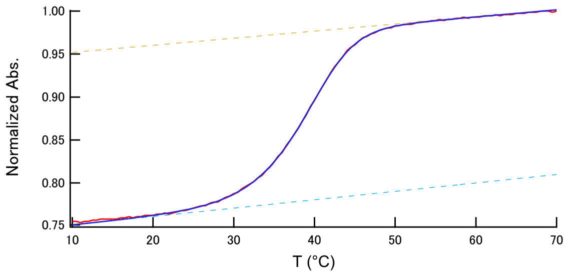

$\Delta G°_{37}$ = –8.91 kcal/mol

$\Delta H°$ = –89.60 kcal/mol

$\Delta S°$ = –260.19 cal/mol·K

T_m = 38.85°C

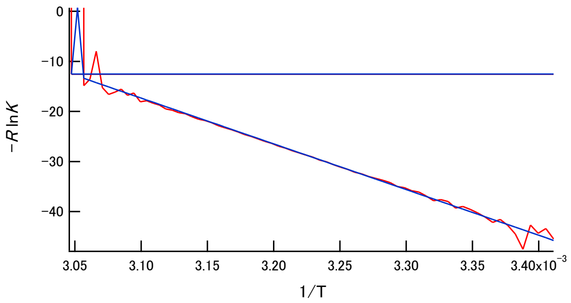

$\Delta G°_{37vh}$ = –8.90 kcal/mol

$\Delta H°_{vh}$ = –91.36 kcal/mol

$\Delta S°_{vh}$ = –265.87 cal/mol·K

$T_{m\ vh}$ = 38.78°C

i(1/RNA) C_t = 4.29 μM

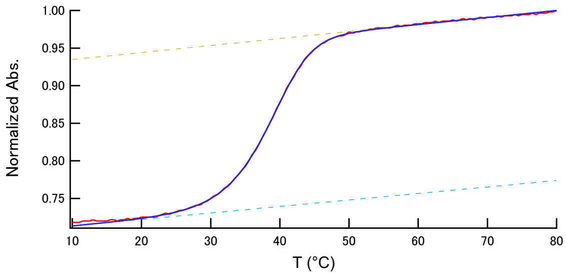

$\Delta G°_{37}$ = –8.91 kcal/mol

$\Delta H°$ = –90.86 kcal/mol

$\Delta S°$ = –264.21 cal/mol·K

T_m = 38.52°C

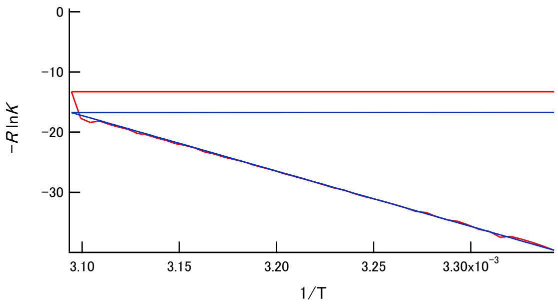

$\Delta G°_{37vh}$ = –8.90 kcal/mol

$\Delta H°_{vh}$ = –92.15 kcal/mol

$\Delta S°_{vh}$ = –268.43 cal/mol·K

$T_{m\ vh}$ = 38.45°C

i(2/RNA) C_t = 30 µM

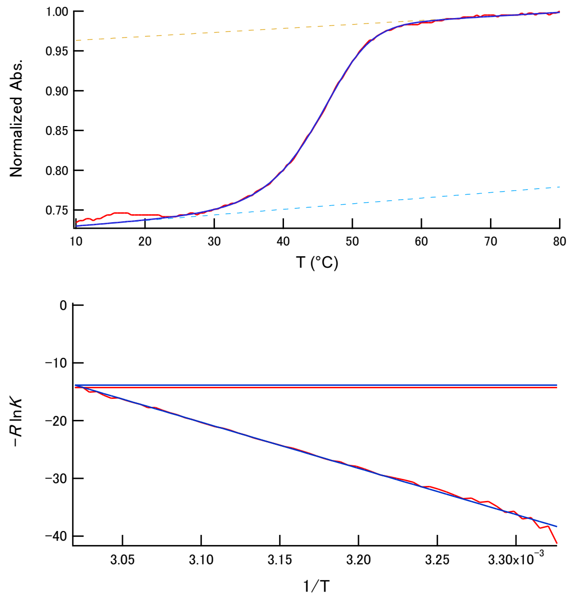

ΔG°_{37} = –9.33 kcal/mol

ΔH° = –78.69 kcal/mol

ΔS° = –223.64 cal/mol·K

T_m = 45.31°C

ΔG°_{37vh} = –9.37 kcal/mol

ΔH°_{vh} = –79.98 kcal/mol

ΔS°_{vh} = –227.65 cal/mol·K

$T_{m\ vh}$ = 45.36°C

i(2/RNA) C_t = 20 µM

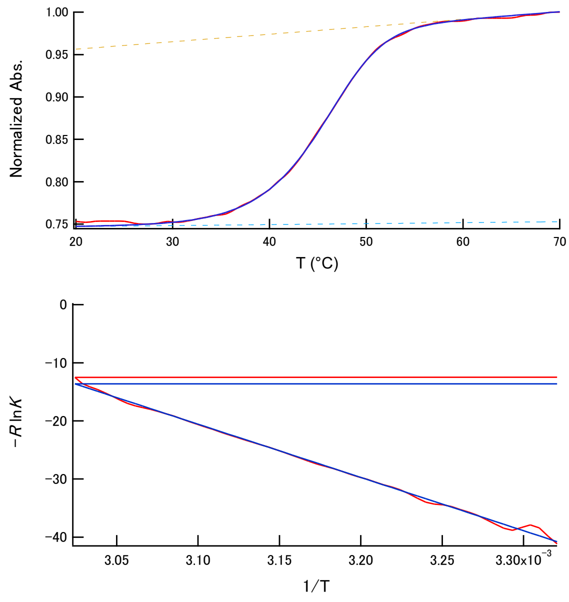

ΔG°_{37} = –9.92 kcal/mol

ΔH° = –91.50 kcal/mol

ΔS° = –263.01 cal/mol·K

T_m = 45.36°C

ΔG°_{37vh} = –9.91 kcal/mol

ΔH°_{vh} = –91.65 kcal/mol

ΔS°_{vh} = –263.56 cal/mol·K

$T_{m\ vh}$ = 45.30°C

i(2/RNA) C_t = 15 μM

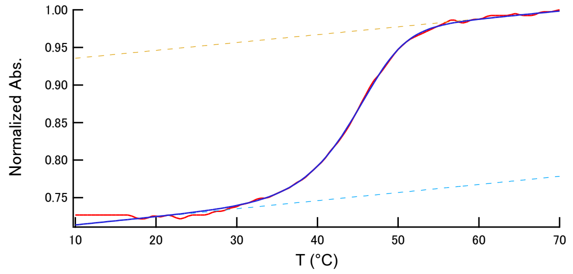

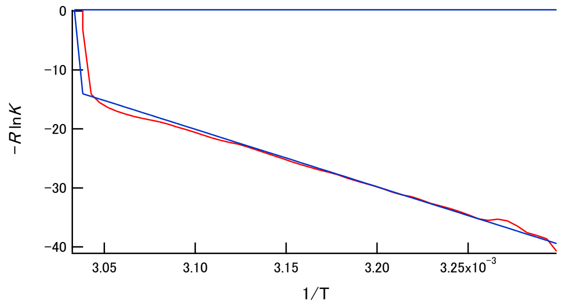

$\Delta G°_{37}$ = –9.95 kcal/mol

$\Delta H°$ = –92.62 kcal/mol

$\Delta S°$ = –266.55 cal/mol·K

T_m = 44.72°C

$\Delta G°_{37vh}$ = –9.98 kcal/mol

$\Delta H°_{vh}$ = –97.50 kcal/mol

$\Delta S°_{vh}$ = –282.18 cal/mol·K

$T_{m\ vh}$ = 44.43°C

i(2/RNA) C_t = 10 μM

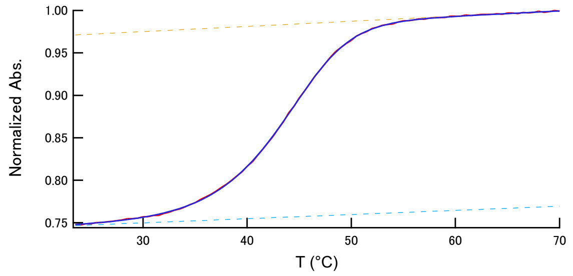

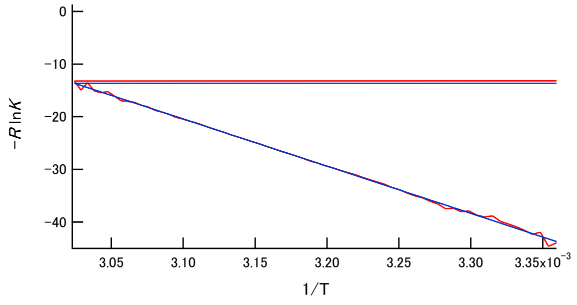

$\Delta G°_{37}$ = –9.79 kcal/mol

$\Delta H°$ = –89.42 kcal/mol

$\Delta S°$ = –256.77 cal/mol·K

T_m = 43.51°C

$\Delta G°_{37vh}$ = –9.79 kcal/mol

$\Delta H°_{vh}$ = –89.68 kcal/mol

$\Delta S°_{vh}$ = –257.59 cal/mol·K

$T_{m\ vh}$ = 43.50°C

i(2/RNA) C_t = 5 μM

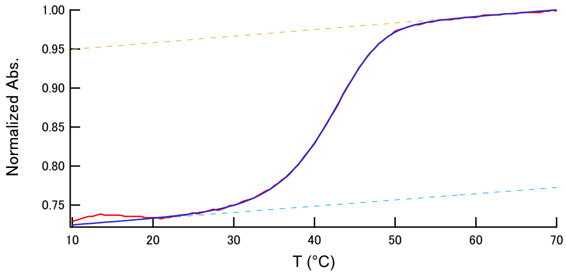

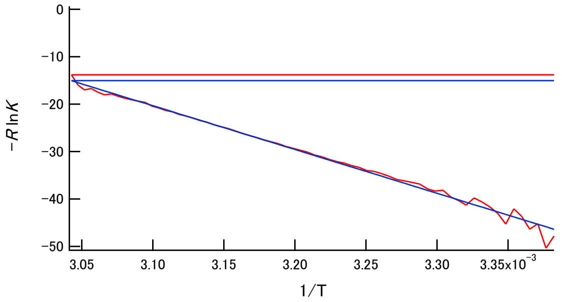

$\Delta G°_{37}$ = –9.80 kcal/mol

$\Delta H°$ = –89.54 kcal/mol

$\Delta S°$ = –257.10 cal/mol·K

T_m = 42.01°C

$\Delta G°_{37vh}$ = –9.87 kcal/mol

$\Delta H°_{vh}$ = –92.33 kcal/mol

$\Delta S°_{vh}$ = –265.89 cal/mol·K

$T_{m\ vh}$ = 42.09°C

i(2/RNA) C_t = 4.29 μM

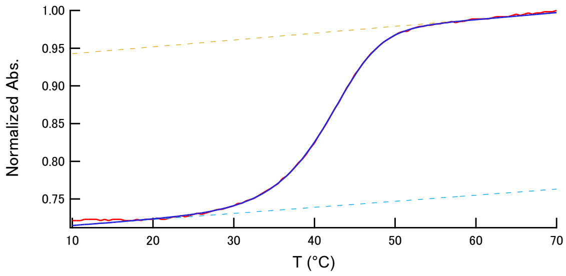

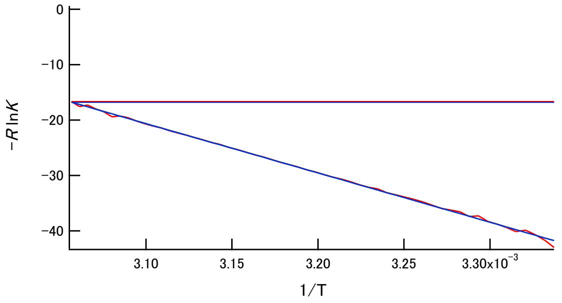

$\Delta G°_{37}$ = –9.81 kcal/mol

$\Delta H°$ = –88.36 kcal/mol

$\Delta S°$ = –253.26 cal/mol·K

T_m = 41.78°C

$\Delta G°_{37vh}$ = –9.83 kcal/mol

$\Delta H°_{vh}$ = –89.04 kcal/mol

$\Delta S°_{vh}$ = –255.41 cal/mol·K

$T_{m\ vh}$ = 41.79°C

i(3/RNA) C_t = 30 μM

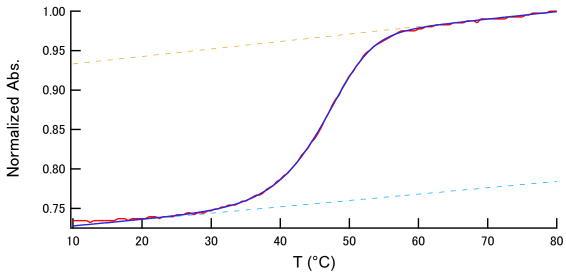

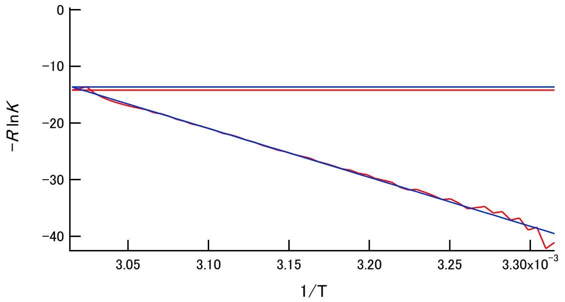

$\Delta G°_{37}$ = –9.78 kcal/mol

$\Delta H°$ = –84.71 kcal/mol

$\Delta S°$ = –241.61 cal/mol·K

T_m = 46.45°C

$\Delta G°_{37vh}$ = –9.83 kcal/mol

$\Delta H°_{vh}$ = –86.39 kcal/mol

$\Delta S°_{vh}$ = –246.84 cal/mol·K

$T_{m\ vh}$ = 46.46°C

i(3/RNA) C_t = 20 μM

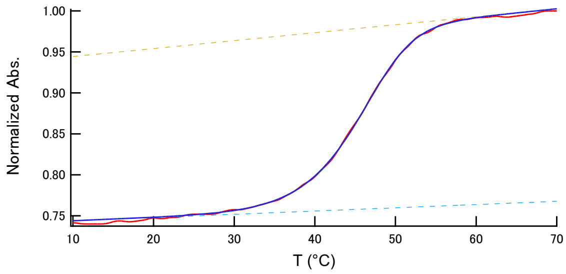

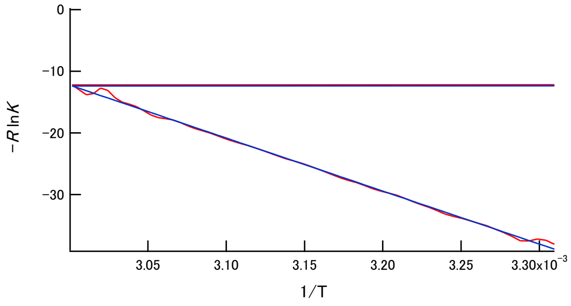

$\Delta G°_{37}$ = –9.79 kcal/mol

$\Delta H°$ = –85.80 kcal/mol

$\Delta S°$ = –245.08 cal/mol·K

T_m = 45.41°C

$\Delta G°_{37vh}$ = –9.77 kcal/mol

$\Delta H°_{vh}$ = –85.96 kcal/mol

$\Delta S°_{vh}$ = –245.63 cal/mol·K

$T_{m\ vh}$ = 45.34°C

i(3/RNA) C_t = 15 µM

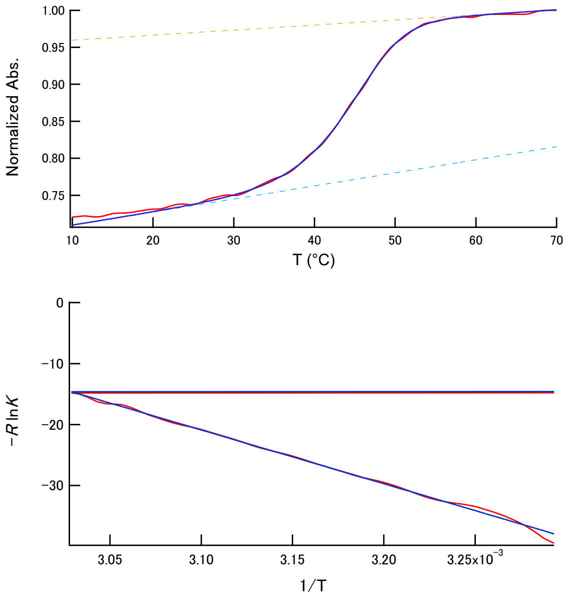

$\Delta G°_{37}$ = –9.84 kcal/mol

$\Delta H°$ = –87.23 kcal/mol

$\Delta S°$ = –249.53 cal/mol·K

T_m = 44.81°C

$\Delta G°_{37vh}$ = –9.87 kcal/mol

$\Delta H°_{vh}$ = –88.17 kcal/mol

$\Delta S°_{vh}$ = –252.45 cal/mol·K

$T_{m\ vh}$ = 44.84°C

i(3/RNA) C_t = 10 µM

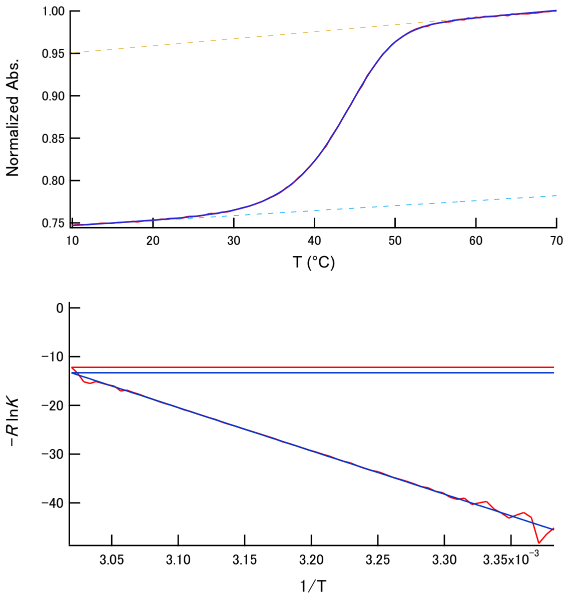

$\Delta G°_{37}$ = –9.75 kcal/mol

$\Delta H°$ = –88.11 kcal/mol

$\Delta S°$ = –252.66 cal/mol·K

T_m = 43.46°C

$\Delta G°_{37vh}$ = –9.76 kcal/mol

$\Delta H°_{vh}$ = –88.77 kcal/mol

$\Delta S°_{vh}$ = –254.73 cal/mol·K

$T_{m\ vh}$ = 43.47°C

i(3/RNA) C_t = 5 μM

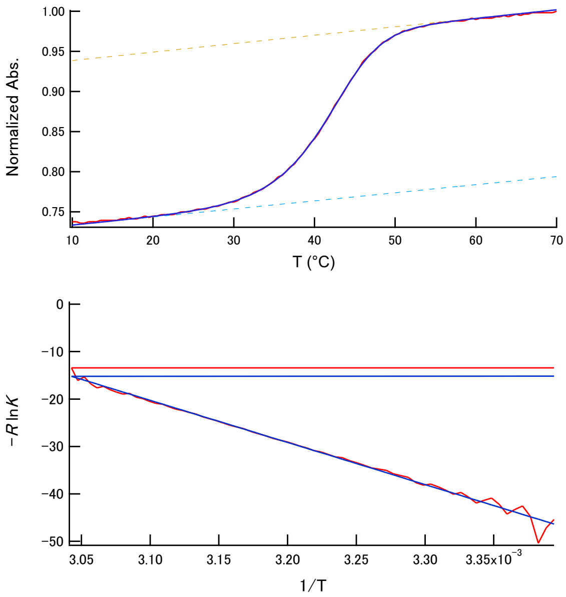

ΔG°_{37} = –9.69 kcal/mol

ΔH° = –87.00 kcal/mol

ΔS° = –249.28 cal/mol·K

T_m = 41.74°C

ΔG°_{37vh} = –9.71 kcal/mol

ΔH°_{vh} = –88.82 kcal/mol

ΔS°_{vh} = –255.07 cal/mol·K

$T_{m\ vh}$ = 41.73°C

i(3/RNA) C_t = 4.29 μM

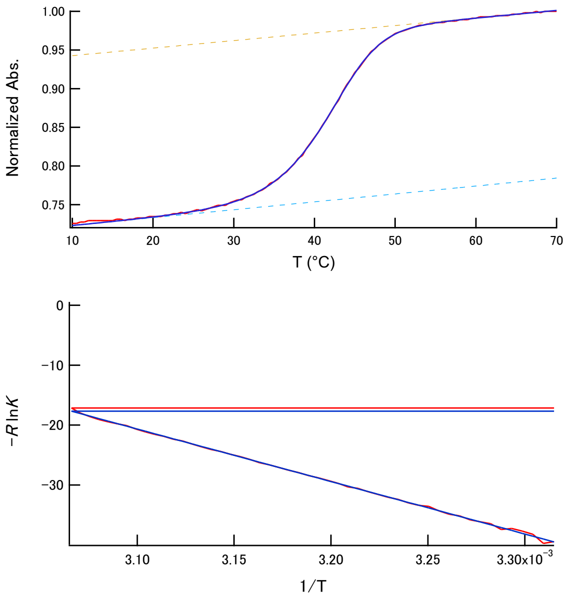

ΔG°_{37} = –9.78 kcal/mol

ΔH° = –87.32 kcal/mol

ΔS° = –250.00 cal/mol·K

T_m = 41.73°C

ΔG°_{37vh} = –9.78 kcal/mol

ΔH°_{vh} = –87.61 kcal/mol

ΔS°_{vh} = –250.93 cal/mol·K

$T_{m\ vh}$ = 41.71°C

i(4/RNA) C_t = 30 µM

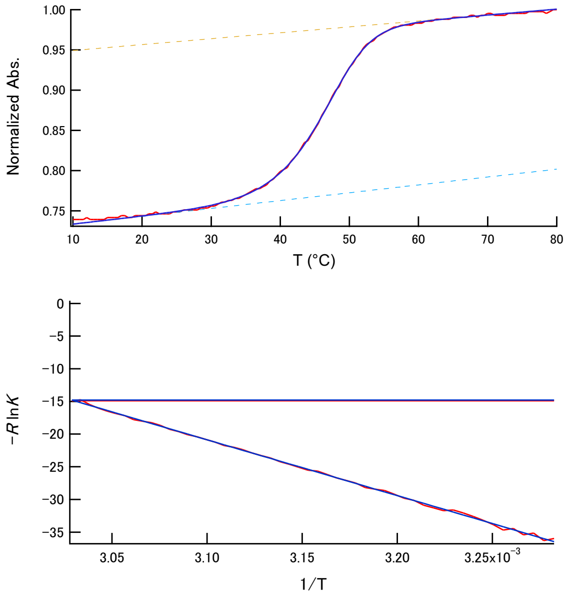

$\Delta G°_{37}$ = –9.77 kcal/mol

$\Delta H°$ = –85.39 kcal/mol

$\Delta S°$ = –243.85 cal/mol·K

T_m = 46.33°C

$\Delta G°_{37vh}$ = –9.77 kcal/mol

$\Delta H°_{vh}$ = –85.43 kcal/mol

$\Delta S°_{vh}$ = –243.96 cal/mol·K

$T_{m\ vh}$ = 46.32°C

i(4/RNA) C_t = 20 µM

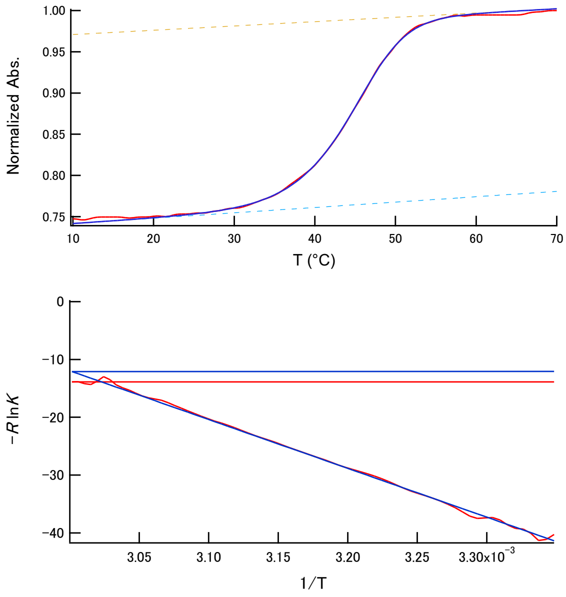

$\Delta G°_{37}$ = –9.57 kcal/mol

$\Delta H°$ = –85.35 kcal/mol

$\Delta S°$ = –244.33 cal/mol·K

T_m = 44.62°C

$\Delta G°_{37vh}$ = –9.58 kcal/mol

$\Delta H°_{vh}$ = –84.47 kcal/mol

$\Delta S°_{vh}$ = –241.46 cal/mol·K

$T_{m\ vh}$ = 44.74°C

i(4/RNA) C_t = 15 µM

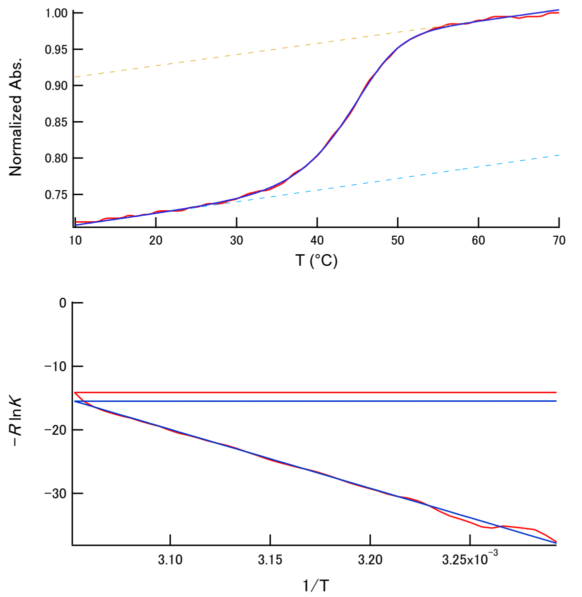

$\Delta G°_{37}$ = –9.77 kcal/mol

$\Delta H°$ = –92.71 kcal/mol

$\Delta S°$ = –267.42 cal/mol·K

T_m = 44.08°C

$\Delta G°_{37vh}$ = –9.75 kcal/mol

$\Delta H°_{vh}$ = –92.69 kcal/mol

$\Delta S°_{vh}$ = –267.40 cal/mol·K

$T_{m\ vh}$ = 44.03°C

i(4/RNA) C_t = 10 µM

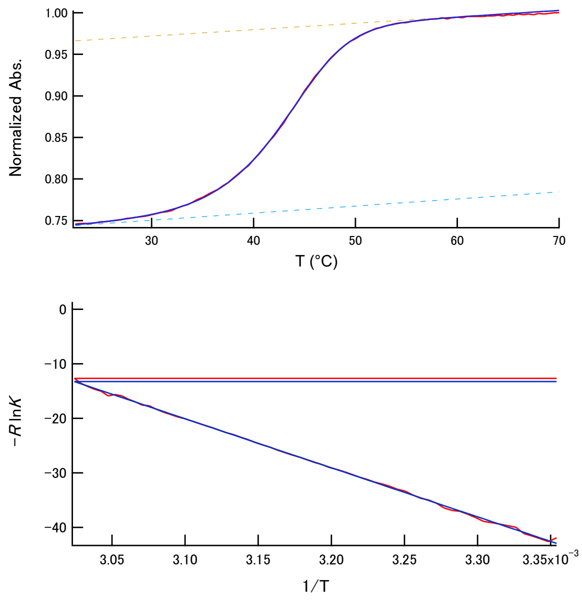

$\Delta G°_{37}$ = –9.69 kcal/mol

$\Delta H°$ = –89.82 kcal/mol

$\Delta S°$ = –258.35 cal/mol·K

T_m = 43.13°C

$\Delta G°_{37vh}$ = –9.69 kcal/mol

$\Delta H°_{vh}$ = –89.96 kcal/mol

$\Delta S°_{vh}$ = –258.80 cal/mol·K

$T_{m\ vh}$ = 43.13°C

i(4/RNA) C_t = 5 μM

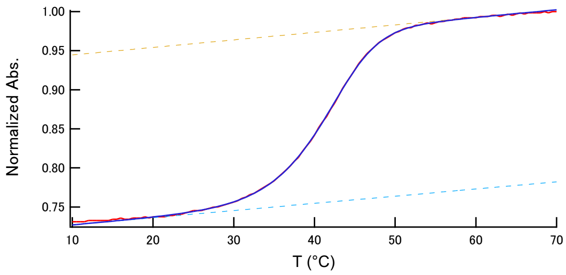

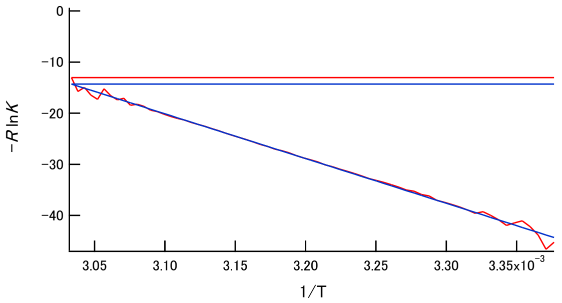

$\Delta G°_{37}$ = –9.59 kcal/mol

$\Delta H°$ = –86.43 kcal/mol

$\Delta S°$ = –247.77 cal/mol·K

T_m = 41.41°C

$\Delta G°_{37vh}$ = –9.61 kcal/mol

$\Delta H°_{vh}$ = –87.52 kcal/mol

$\Delta S°_{vh}$ = –251.19 cal/mol·K

$T_{m\ vh}$ = 41.43°C

i(4/RNA) C_t = 4.29 μM

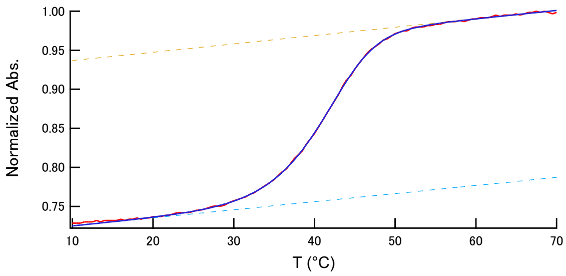

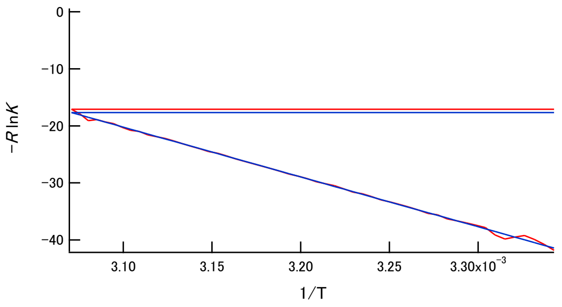

$\Delta G°_{37}$ = –9.64 kcal/mol

$\Delta H°$ = –87.16 kcal/mol

$\Delta S°$ = –249.95 cal/mol·K

T_m = 41.21°C

$\Delta G°_{37vh}$ = –9.64 kcal/mol

$\Delta H°_{vh}$ = –87.34 kcal/mol

$\Delta S°_{vh}$ = –250.54 cal/mol·K

$T_{m\ vh}$ = 41.20°C

i(5/RNA) C_t = 30 µM

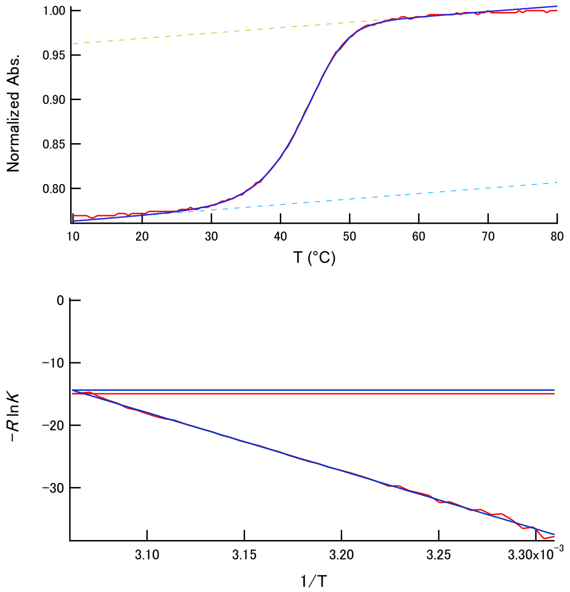

$\Delta G°_{37}$ = –9.16 kcal/mol

$\Delta H°$ = –92.59 kcal/mol

$\Delta S°$ = –269.01 cal/mol·K

T_m = 43.44°C

$\Delta G°_{37vh}$ = –9.17 kcal/mol

$\Delta H°_{vh}$ = –93.23 kcal/mol

$\Delta S°_{vh}$ = –271.03 cal/mol·K

$T_{m\ vh}$ = 43.44°C

i(5/RNA) C_t = 20 µM

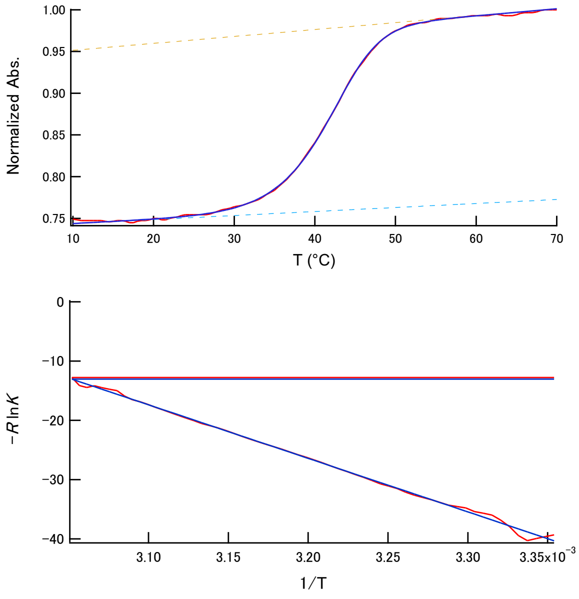

$\Delta G°_{37}$ = –8.88 kcal/mol

$\Delta H°$ = –90.15 kcal/mol

$\Delta S°$ = –262.04 cal/mol·K

T_m = 41.73°C

$\Delta G°_{37vh}$ = –8.87 kcal/mol

$\Delta H°_{vh}$ = –90.34 kcal/mol

$\Delta S°_{vh}$ = –262.68 cal/mol·K

$T_{m\ vh}$ = 41.71°C

i(5/RNA) C_t = 15 μM

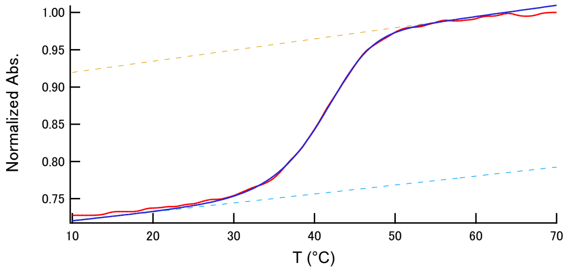

$\Delta G°_{37}$ = –8.92 kcal/mol

$\Delta H°$ = –93.49 kcal/mol

$\Delta S°$ = –272.67 cal/mol·K

T_m = 41.11°C

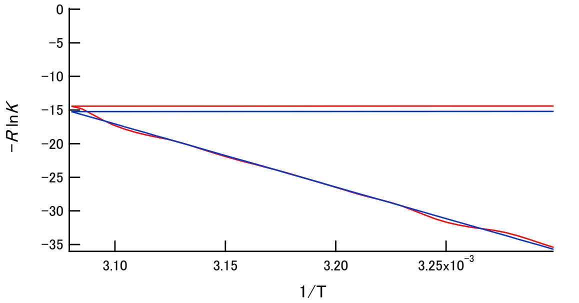

$\Delta G°_{37vh}$ = –8.91 kcal/mol

$\Delta H°_{vh}$ = –93.72 kcal/mol

$\Delta S°_{vh}$ = –273.46 cal/mol·K

$T_{m\ vh}$ = 41.05°C

i(5/RNA) C_t = 10 μM

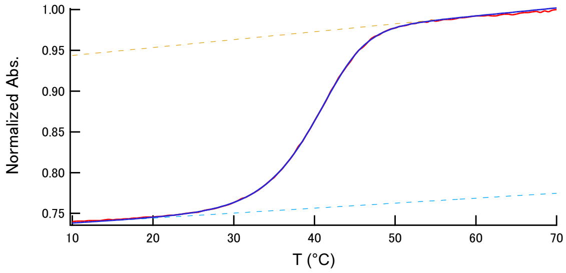

$\Delta G°_{37}$ = –8.84 kcal/mol

$\Delta H°$ = –90.25 kcal/mol

$\Delta S°$ = –262.47 cal/mol·K

T_m = 40.09°C

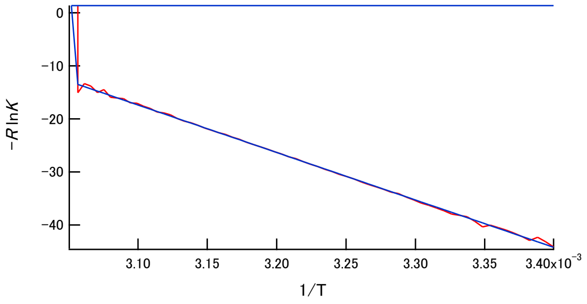

$\Delta G°_{37vh}$ = –8.84 kcal/mol

$\Delta H°_{vh}$ = –89.57 kcal/mol

$\Delta S°_{vh}$ = –260.30 cal/mol·K

$T_{m\ vh}$ = 40.10°C

i(5/RNA) C_t = 4.29 μM

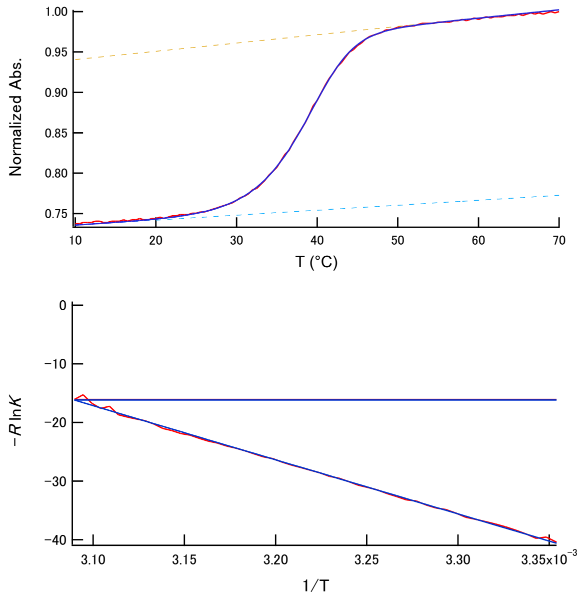

$\Delta G°_{37}$ = –8.89 kcal/mol

$\Delta H°$ = –91.73 kcal/mol

$\Delta S°$ = –267.10 cal/mol·K

T_m = 38.43°C

$\Delta G°_{37vh}$ = –8.87 kcal/mol

$\Delta H°_{vh}$ = –92.40 kcal/mol

$\Delta S°_{vh}$ = –269.31 cal/mol·K

$T_{m\ vh}$ = 38.36°C

i(6/RNA) C_t = 30 μM

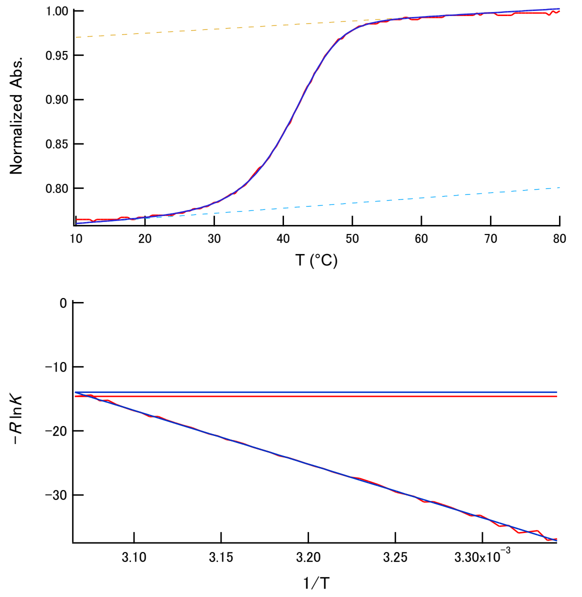

$\Delta G°_{37}$ = –8.44 kcal/mol

$\Delta H°$ = –83.74 kcal/mol

$\Delta S°$ = –242.79 cal/mol·K

T_m = 41.37°C

$\Delta G°_{37vh}$ = –8.44 kcal/mol

$\Delta H°_{vh}$ = –83.77 kcal/mol

$\Delta S°_{vh}$ = –242.88 cal/mol·K

$T_{m\ vh}$ = 41.38°C

i(6/RNA) C_t = 20 μM

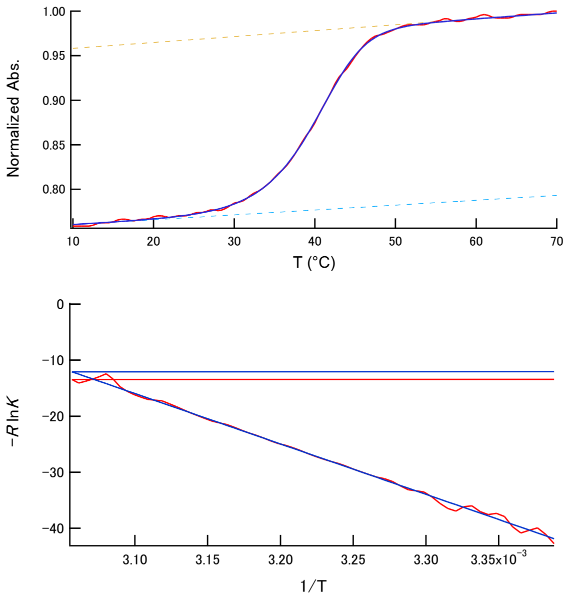

$\Delta G°_{37}$ = –8.41 kcal/mol

$\Delta H°$ = –90.19 kcal/mol

$\Delta S°$ = –263.66 cal/mol·K

T_m = 40.09°C

$\Delta G°_{37vh}$ = –8.42 kcal/mol

$\Delta H°_{vh}$ = –89.93 kcal/mol

$\Delta S°_{vh}$ = –262.83 cal/mol·K

$T_{m\ vh}$ = 40.11°C

i(6/RNA) C_t = 15 μM

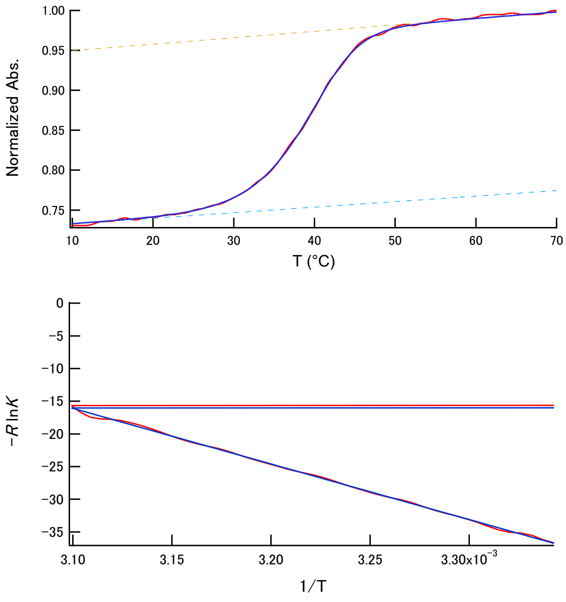

$\Delta G°_{37}$ = –8.27 kcal/mol

$\Delta H°$ = –85.21 kcal/mol

$\Delta S°$ = –248.08 cal/mol·K

T_m = 39.08°C

$\Delta G°_{37vh}$ = –8.27 kcal/mol

$\Delta H°_{vh}$ = –85.07 kcal/mol

$\Delta S°_{vh}$ = –247.64 cal/mol·K

$T_{m\ vh}$ = 39.09°C

i(6/RNA) C_t = 10 μM

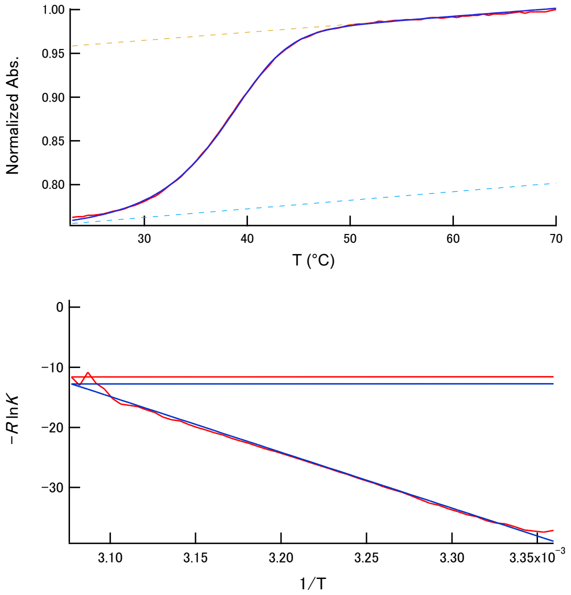

$\Delta G°_{37}$ = –8.24 kcal/mol

$\Delta H°$ = –90.89 kcal/mol

$\Delta S°$ = –266.48 cal/mol·K

T_m = 37.99°C

$\Delta G°_{37vh}$ = –8.19 kcal/mol

$\Delta H°_{vh}$ = –92.79 kcal/mol

$\Delta S°_{vh}$ = –272.79 cal/mol·K

$T_{m\ vh}$ = 37.79°C

i(6/RNA) C_t = 5 μM

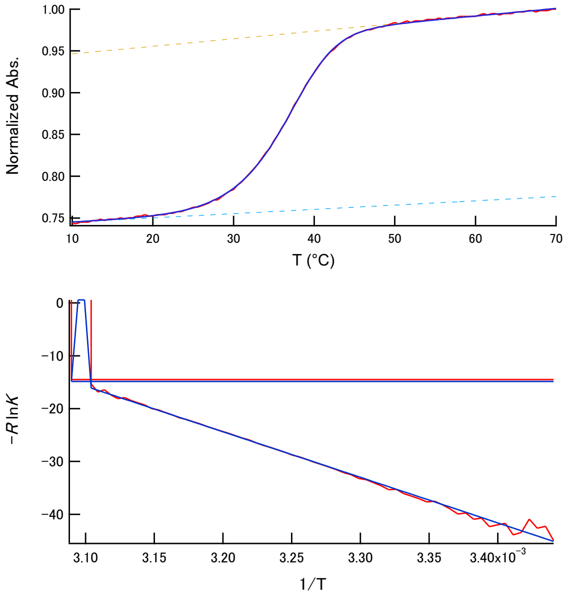

$\Delta G°_{37}$ = –8.21 kcal/mol

$\Delta H°$ = –87.72 kcal/mol

$\Delta S°$ = –256.35 cal/mol·K

T_m = 36.40°C

$\Delta G°_{37vh}$ = –8.21 kcal/mol

$\Delta H°_{vh}$ = –86.22 kcal/mol

$\Delta S°_{vh}$ = –251.54 cal/mol·K

$T_{m\ vh}$ = 36.39°C

i(6/RNA) C_t = 4.29 μM

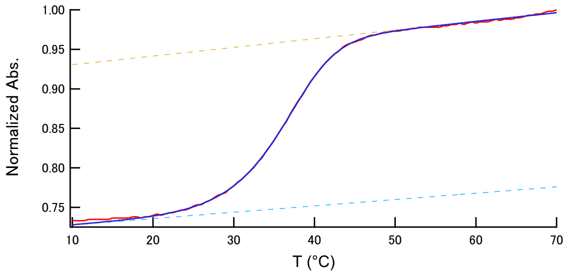

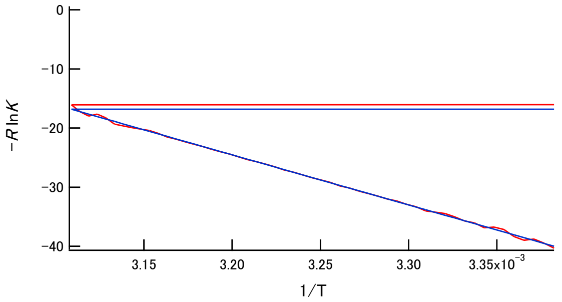

$\Delta G°_{37}$ = –8.25 kcal/mol

$\Delta H°$ = –84.22 kcal/mol

$\Delta S°$ = –244.94 cal/mol·K

T_m = 36.19°C

$\Delta G°_{37vh}$ = –8.25 kcal/mol

$\Delta H°_{vh}$ = –84.73 kcal/mol

$\Delta S°_{vh}$ = –246.61 cal/mol·K

$T_{m\ vh}$ = 36.18°C

ii(DNA/RNA) C_t = 30 μM

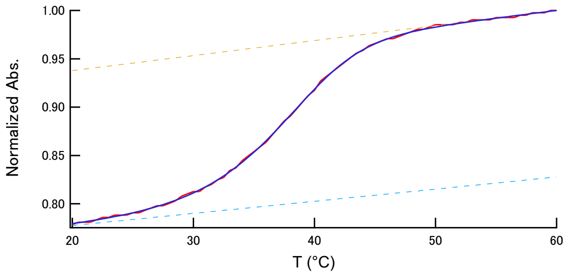

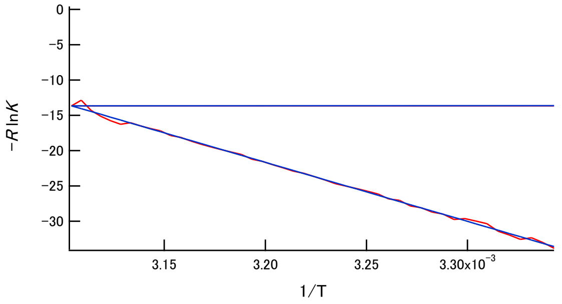

$\Delta G°_{37}$ = –7.35 kcal/mol

$\Delta H°$ = –83.31 kcal/mol

$\Delta S°$ = –244.93 cal/mol·K

T_m = 37.29°C

$\Delta G°_{37vh}$ = –7.35 kcal/mol

$\Delta H°_{vh}$ = –83.57 kcal/mol

$\Delta S°_{vh}$ = –245.75 cal/mol·K

$T_{m\ vh}$ = 37.28°C

ii(DNA/RNA) C_t = 24 μM

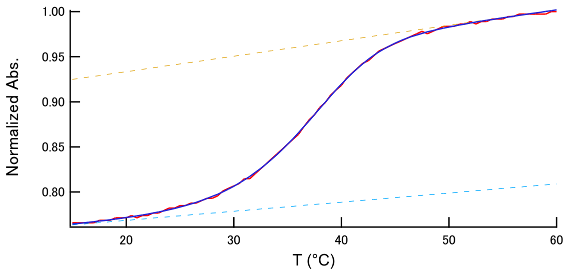

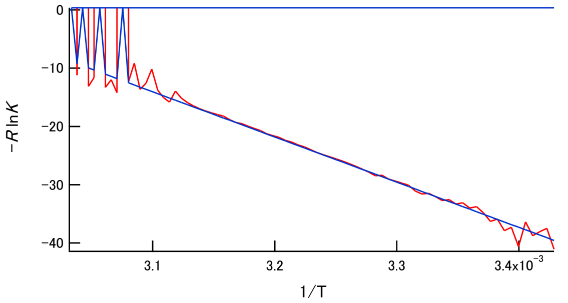

$\Delta G°_{37}$ = –7.31 kcal/mol

$\Delta H°$ = –78.38 kcal/mol

$\Delta S°$ = –229.14 cal/mol·K

T_m = 36.61°C

$\Delta G°_{37vh}$ = –7.35 kcal/mol

$\Delta H°_{vh}$ = –77.60 kcal/mol

$\Delta S°_{vh}$ = –226.52 cal/mol·K

$T_{m\ vh}$ = 36.75°C

ii(DNA/RNA) C_t = 15 μM

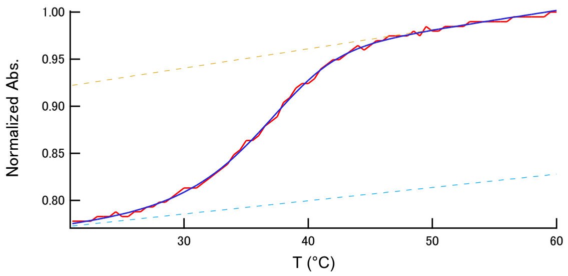

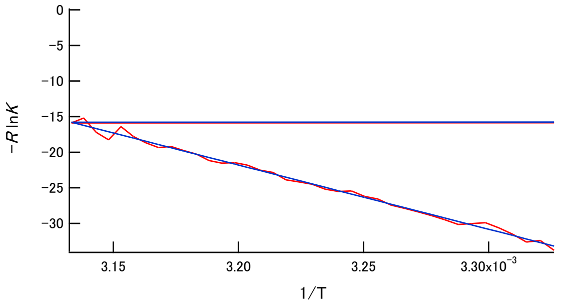

$\Delta G°_{37}$ = –7.44 kcal/mol

$\Delta H°$ = –89.54 kcal/mol

$\Delta S°$ = –264.70 cal/mol·K

T_m = 36.12°C

$\Delta G°_{37vh}$ = –7.44 kcal/mol

$\Delta H°_{vh}$ = –90.23 kcal/mol

$\Delta S°_{vh}$ = –266.95 cal/mol·K

$T_{m\ vh}$ = 36.11°C

ii(DNA/RNA) C_t = 12 μM

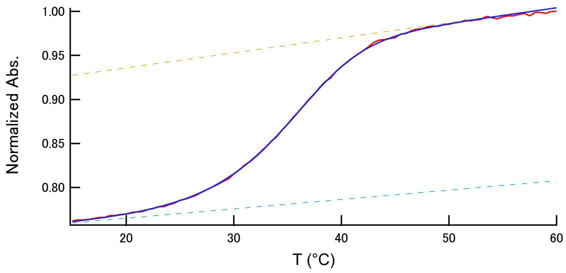

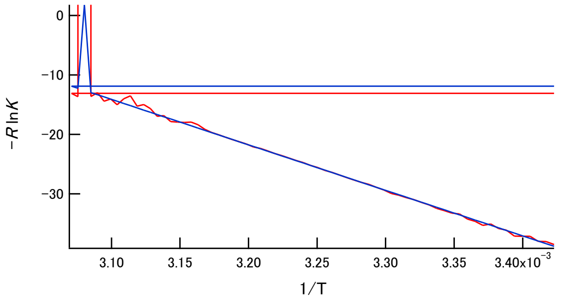

$\Delta G°_{37}$ = –7.33 kcal/mol

$\Delta H°$ = –76.46 kcal/mol

$\Delta S°$ = –222.90 cal/mol·K

T_m = 34.94°C

$\Delta G°_{37vh}$ = –7.33 kcal/mol

$\Delta H°_{vh}$ = –76.51 kcal/mol

$\Delta S°_{vh}$ = –223.06 cal/mol·K

$T_{m\ vh}$ = 34.96°C

ii(DNA/RNA) C_t = 7.5 μM

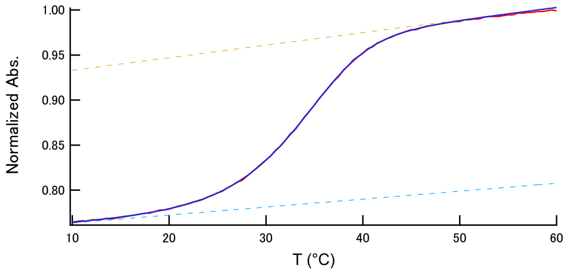

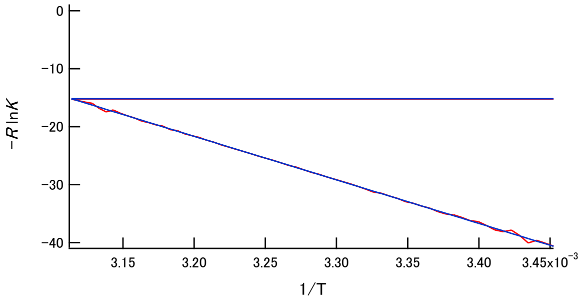

$\Delta G°_{37}$ = –7.28 kcal/mol

$\Delta H°$ = –75.09 kcal/mol

$\Delta S°$ = –218.62 cal/mol·K

T_m = 33.55°C

$\Delta G°_{37vh}$ = –7.28 kcal/mol

$\Delta H°_{vh}$ = –75.17 kcal/mol

$\Delta S°_{vh}$ = –218.89 cal/mol·K

$T_{m\ vh}$ = 33.55°C

ii(DNA/RNA) C_t = 6 μM

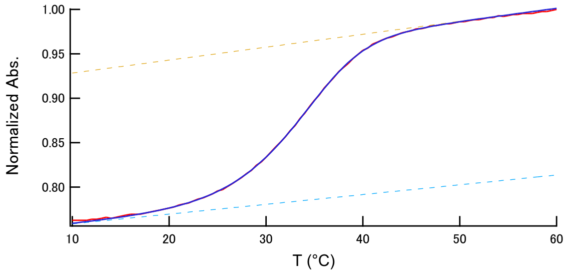

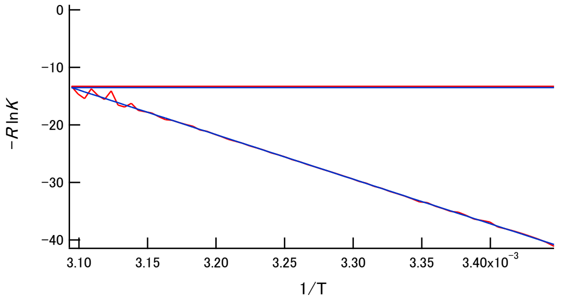

$\Delta G°_{37}$ = –7.32 kcal/mol

$\Delta H°$ = –77.35 kcal/mol

$\Delta S°$ = –225.81 cal/mol·K

T_m = 33.25°C

$\Delta G°_{37vh}$ = –7.31 kcal/mol

$\Delta H°_{vh}$ = –77.52 kcal/mol

$\Delta S°_{vh}$ = –226.37 cal/mol·K

$T_{m\ vh}$ = 33.23°C

ii(DNA/RNA) C_t = 3.75 μM

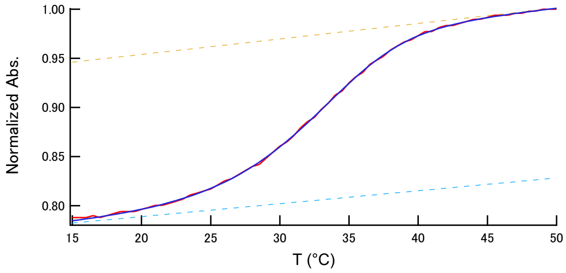

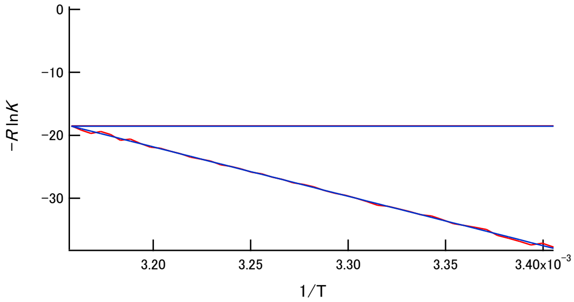

$\Delta G°_{37}$ = –7.37 kcal/mol

$\Delta H°$ = –78.42 kcal/mol

$\Delta S°$ = –229.10 cal/mol·K

T_m = 32.38°C

$\Delta G°_{37vh}$ = –7.37 kcal/mol

$\Delta H°_{vh}$ = –78.46 kcal/mol

$\Delta S°_{vh}$ = –229.24 cal/mol·K

$T_{m\ vh}$ = 32.38°C

ii(1/RNA) C_t = 30 μM

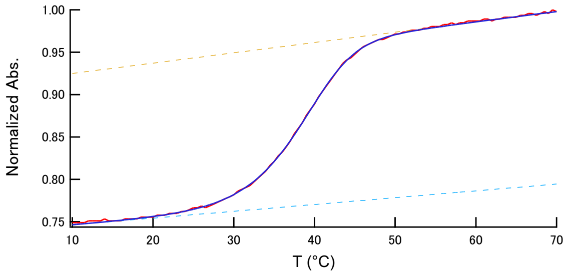

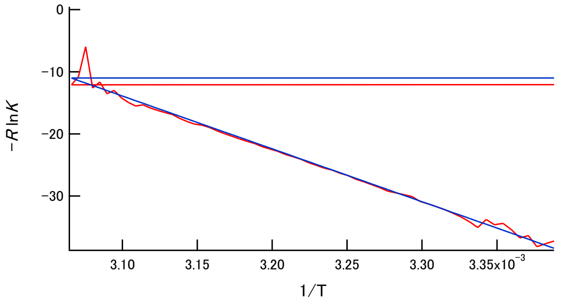

$\Delta G°_{37}$ = –7.63 kcal/mol

$\Delta H°$ = –83.30 kcal/mol

$\Delta S°$ = –243.99 cal/mol·K

T_m = 38.32°C

$\Delta G°_{37vh}$ = –7.59 kcal/mol

$\Delta H°_{vh}$ = –85.05 kcal/mol

$\Delta S°_{vh}$ = –249.75 cal/mol·K

$T_{m\ vh}$ = 38.15°C

ii(1/RNA) C_t = 24 μM

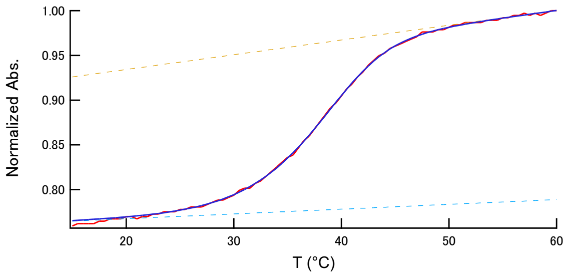

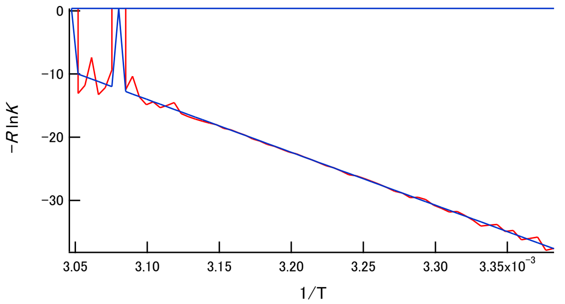

$\Delta G°_{37}$ = –7.58 kcal/mol

$\Delta H°$ = –83.17 kcal/mol

$\Delta S°$ = –243.72 cal/mol·K

T_m = 37.65°C

$\Delta G°_{37vh}$ = –7.58 kcal/mol

$\Delta H°_{vh}$ = –83.63 kcal/mol

$\Delta S°_{vh}$ = –245.23 cal/mol·K

$T_{m\ vh}$ = 37.62°C

ii(1/RNA) C_t = 12 μM

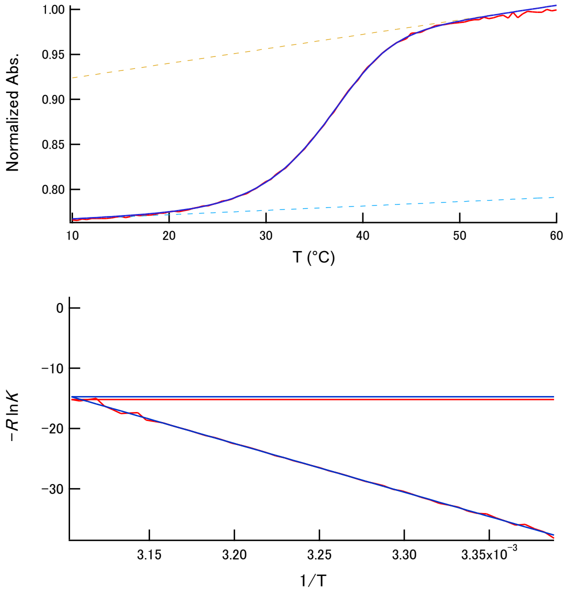

$\Delta G°_{37}$ = –7.58 kcal/mol

$\Delta H°$ = –80.65 kcal/mol

$\Delta S°$ = –235.58 cal/mol·K

T_m = 36.03°C

$\Delta G°_{37vh}$ = –7.58 kcal/mol

$\Delta H°_{vh}$ = –80.79 kcal/mol

$\Delta S°_{vh}$ = –236.05 cal/mol·K

$T_{m\ vh}$ = 36.02°C

ii(1/RNA) C_t = 7.5 μM

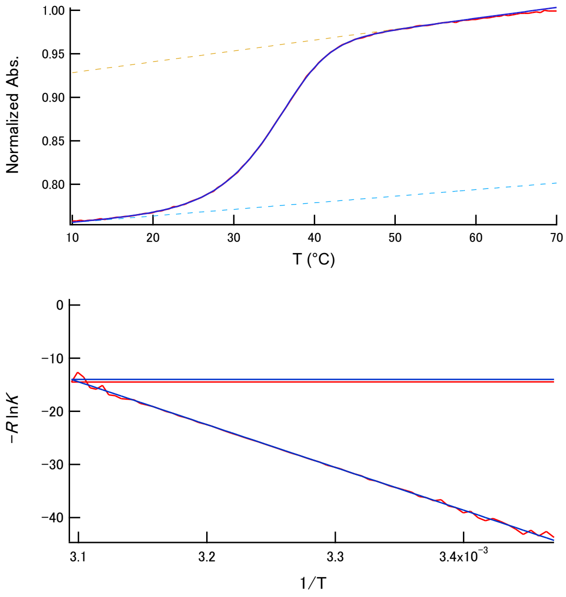

$\Delta G°_{37}$ = –7.60 kcal/mol

$\Delta H°$ = –80.17 kcal/mol

$\Delta S°$ = –233.98 cal/mol·K

T_m = 34.97°C

$\Delta G°_{37vh}$ = –7.58 kcal/mol

$\Delta H°_{vh}$ = –80.53 kcal/mol

$\Delta S°_{vh}$ = –235.21 cal/mol·K

$T_{m\ vh}$ = 34.92°C

ii(1/RNA) C_t = 6 μM

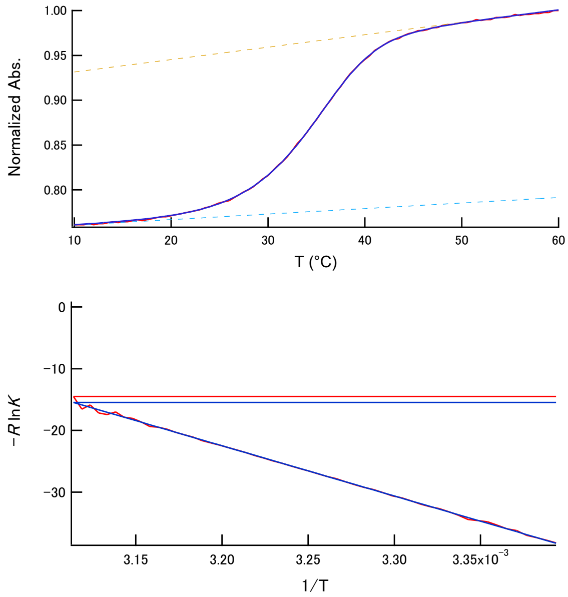

$\Delta G°_{37}$ = –7.59 kcal/mol
$\Delta H°$ = –81.32 kcal/mol
$\Delta S°$ = –237.72 cal/mol·K
T_m = 34.45°C

$\Delta G°_{37vh}$ = –7.59 kcal/mol
$\Delta H°_{vh}$ = –81.50 kcal/mol
$\Delta S°_{vh}$ = –238.32 cal/mol·K
$T_{m\ vh}$ = 34.44°C

ii(2/RNA) C_t = 30 μM

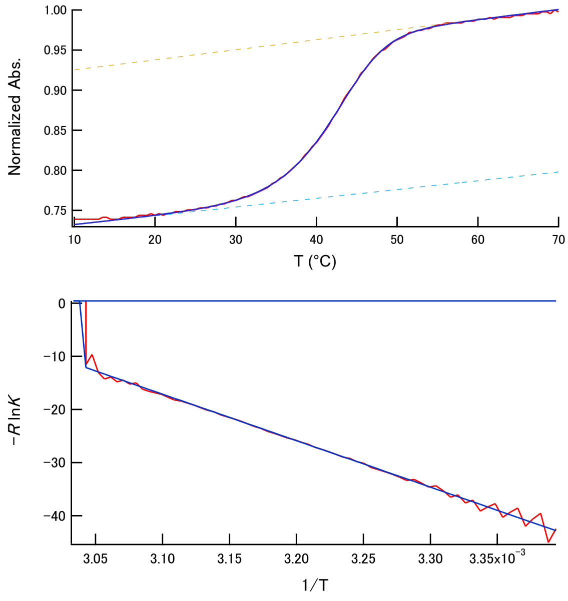

$\Delta G°_{37}$ = –8.68 kcal/mol
$\Delta H°$ = –86.76 kcal/mol
$\Delta S°$ = –251.76 cal/mol·K
T_m = 42.11°C

$\Delta G°_{37vh}$ = –8.68 kcal/mol
$\Delta H°_{vh}$ = –87.39 kcal/mol
$\Delta S°_{vh}$ = –253.78 cal/mol·K
$T_{m\ vh}$ = 42.06°C

ii(2/RNA) C_t = 24 µM

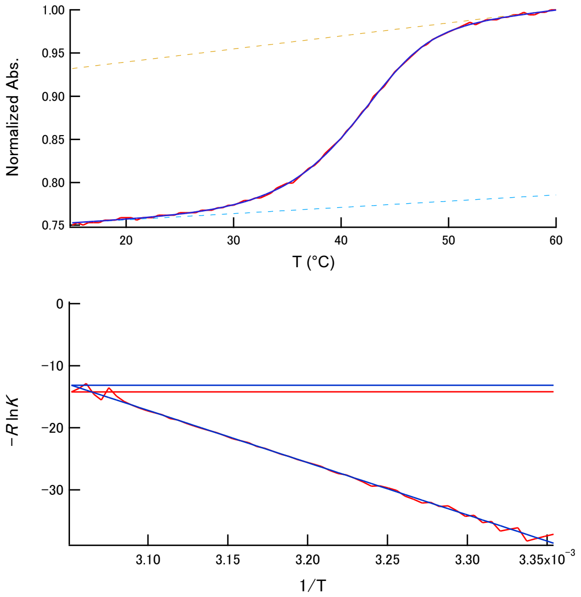

$\Delta G°_{37}$ = –8.59 kcal/mol

$\Delta H°$ = –84.54 kcal/mol

$\Delta S°$ = –244.87 cal/mol·K

T_m = 41.40°C

$\Delta G°_{37vh}$ = –8.58 kcal/mol

$\Delta H°_{vh}$ = –84.33 kcal/mol

$\Delta S°_{vh}$ = –244.24 cal/mol·K

$T_{m\ vh}$ = 41.37°C

ii(2/RNA) C_t = 12 µM

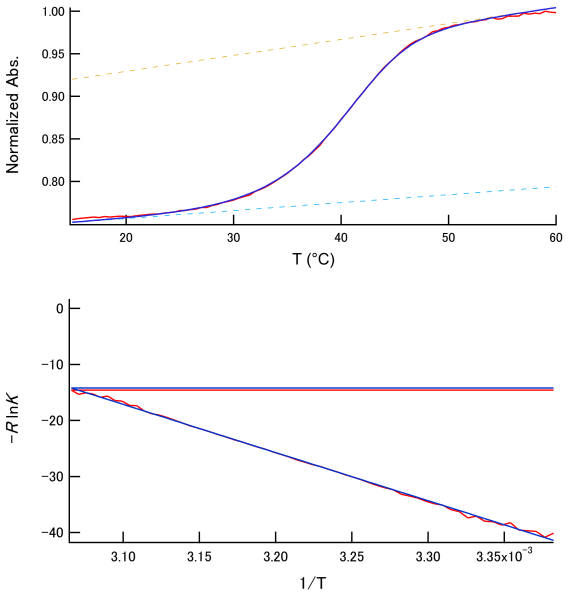

$\Delta G°_{37}$ = –8.62 kcal/mol

$\Delta H°$ = –85.33 kcal/mol

$\Delta S°$ = –247.32 cal/mol·K

T_m = 39.89°C

$\Delta G°_{37vh}$ = –8.63 kcal/mol

$\Delta H°_{vh}$ = –85.98 kcal/mol

$\Delta S°_{vh}$ = –249.41 cal/mol·K

$T_{m\ vh}$ = 39.87°C

ii(2/RNA) C_t = 7.5 μM

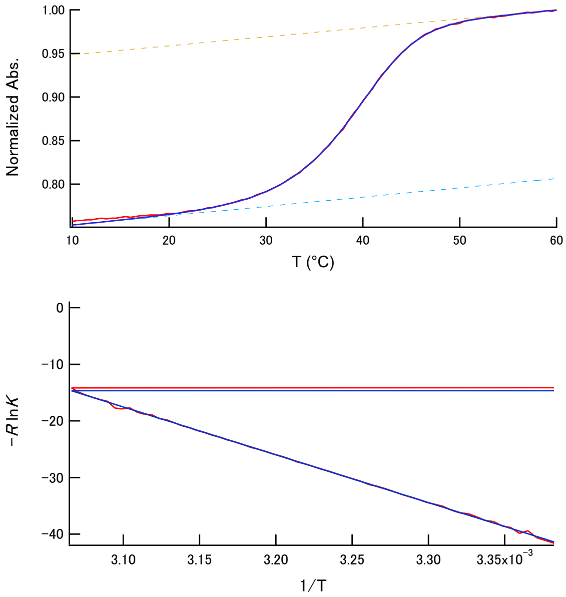

$\Delta G°_{37}$ = –8.69 kcal/mol

$\Delta H°$ = –84.21 kcal/mol

$\Delta S°$ = –243.48 cal/mol·K

T_m = 39.10°C

$\Delta G°_{37vh}$ = –8.69 kcal/mol

$\Delta H°_{vh}$ = –84.46 kcal/mol

$\Delta S°_{vh}$ = –244.29 cal/mol·K

$T_{m\ vh}$ = 39.09°C

ii(2/RNA) C_t = 6 μM

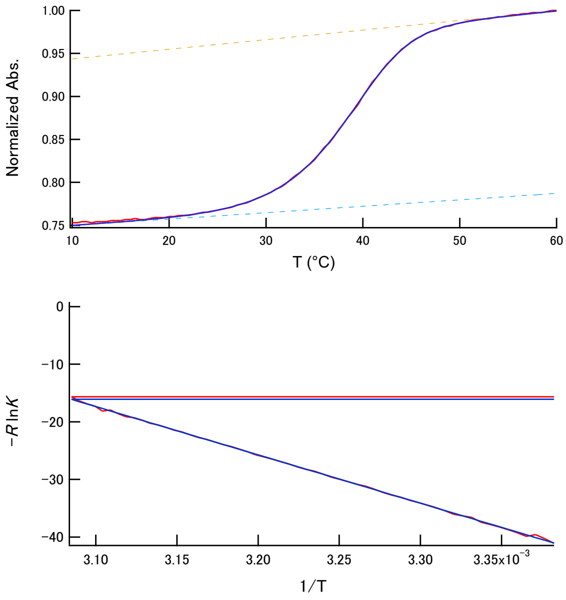

$\Delta G°_{37}$ = –8.62 kcal/mol

$\Delta H°$ = –83.76 kcal/mol

$\Delta S°$ = –242.27 cal/mol·K

T_m = 38.31°C

$\Delta G°_{37vh}$ = –8.61 kcal/mol

$\Delta H°_{vh}$ = –83.91 kcal/mol

$\Delta S°_{vh}$ = –242.77 cal/mol·K

$T_{m\ vh}$ = 38.30°C

ii(3/RNA) C_t = 30 μM

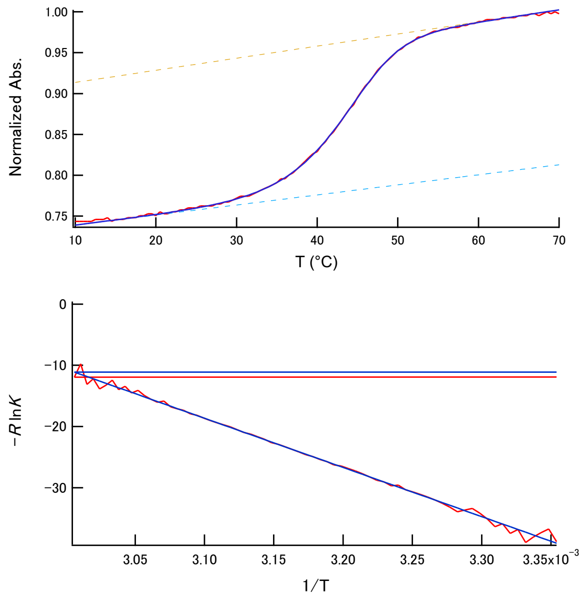

$\Delta G°_{37}$ = –8.88 kcal/mol

$\Delta H°$ = –80.48 kcal/mol

$\Delta S°$ = –230.86 cal/mol·K

T_m = 43.32°C

$\Delta G°_{37vh}$ = –8.88 kcal/mol

$\Delta H°_{vh}$ = –80.38 kcal/mol

$\Delta S°_{vh}$ = –230.54 cal/mol·K

$T_{m\ vh}$ = 43.33°C

ii(3/RNA) C_t = 24 μM

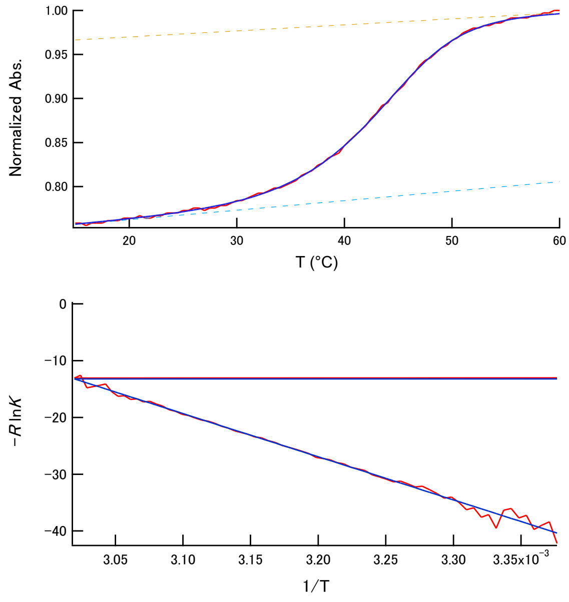

$\Delta G°_{37}$ = –8.91 kcal/mol

$\Delta H°$ = –75.45 kcal/mol

$\Delta S°$ = –214.55 cal/mol·K

T_m = 43.28°C

$\Delta G°_{37vh}$ = –8.92 kcal/mol

$\Delta H°_{vh}$ = –76.05 kcal/mol

$\Delta S°_{vh}$ = –216.45 cal/mol·K

$T_{m\ vh}$ = 43.29°C

ii(3/RNA) C_t = 12 μM

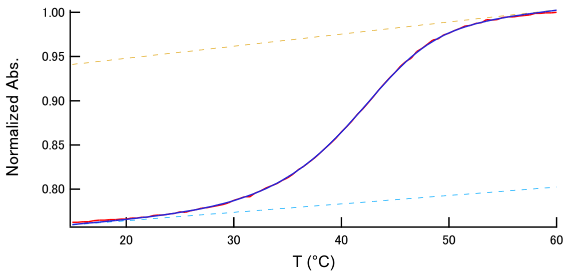

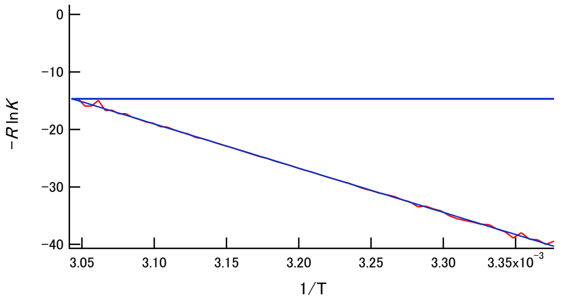

$\Delta G°_{37}$ = –8.87 kcal/mol

$\Delta H°$ = –76.54 kcal/mol

$\Delta S°$ = –218.18 cal/mol·K

T_m = 41.24°C

$\Delta G°_{37vh}$ = –8.87 kcal/mol

$\Delta H°_{vh}$ = –76.95 kcal/mol

$\Delta S°_{vh}$ = –219.49 cal/mol·K

$T_{m\ vh}$ = 41.22°C

ii(3/RNA) C_t = 7.5 μM

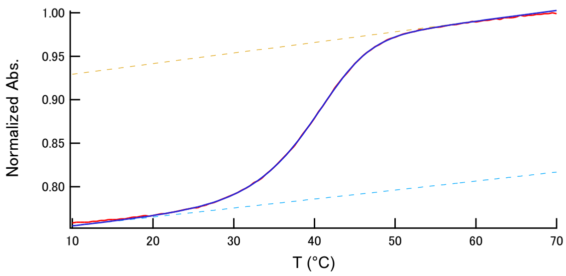

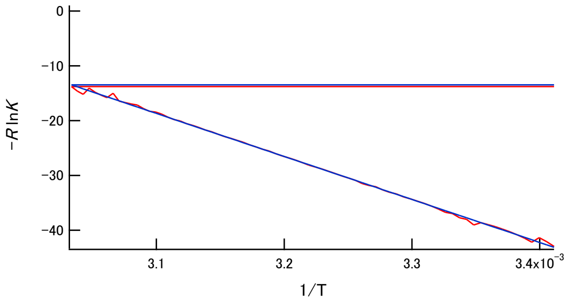

$\Delta G°_{37}$ = –8.82 kcal/mol

$\Delta H°$ = –78.98 kcal/mol

$\Delta S°$ = –226.22 cal/mol·K

T_m = 39.75°C

$\Delta G°_{37vh}$ = –8.82 kcal/mol

$\Delta H°_{vh}$ = –78.48 kcal/mol

$\Delta S°_{vh}$ = –224.62 cal/mol·K

$T_{m\ vh}$ = 39.76°C

ii(3/RNA) C_t = 6 μM

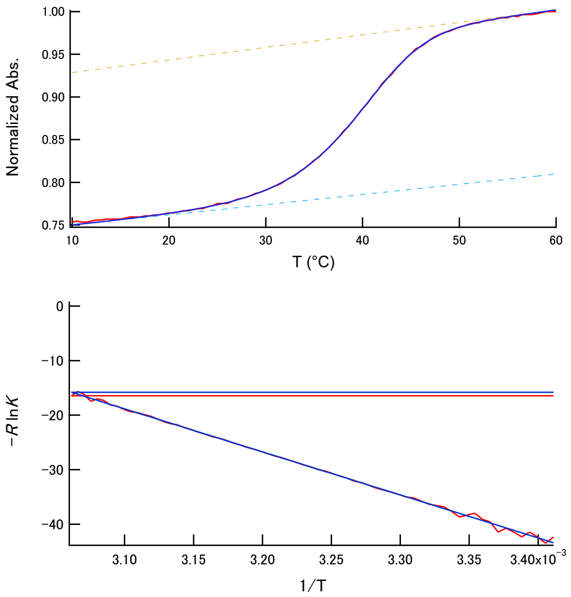

$\Delta G°_{37}$ = –8.89 kcal/mol

$\Delta H°$ = –78.62 kcal/mol

$\Delta S°$ = –224.85 cal/mol·K

T_m = 39.47°C

$\Delta G°_{37vh}$ = –8.89 kcal/mol

$\Delta H°_{vh}$ = –78.86 kcal/mol

$\Delta S°_{vh}$ = –225.62 cal/mol·K

$T_{m\ vh}$ = 39.46°C

ii(4/RNA) C_t = 30 μM

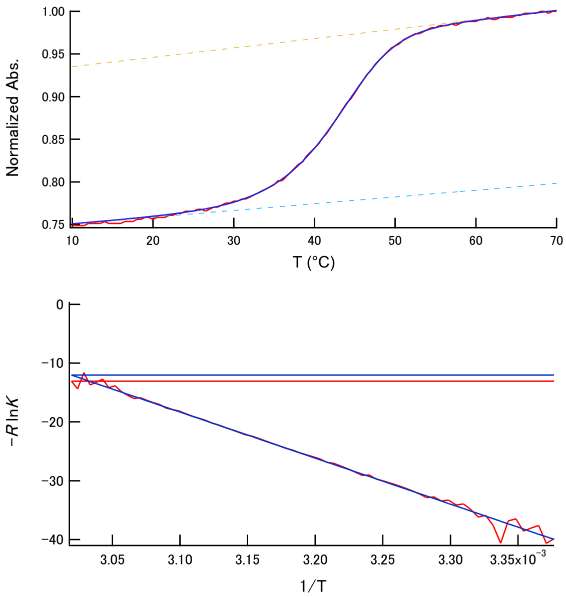

$\Delta G°_{37}$ = –8.68 kcal/mol

$\Delta H°$ = –77.94 kcal/mol

$\Delta S°$ = –223.31 cal/mol·K

T_m = 42.72°C

$\Delta G°_{37vh}$ = –8.70 kcal/mol

$\Delta H°_{vh}$ = –78.32 kcal/mol

$\Delta S°_{vh}$ = –224.47 cal/mol·K

$T_{m\ vh}$ = 42.76°C

ii(4/RNA) C_t = 24 μM

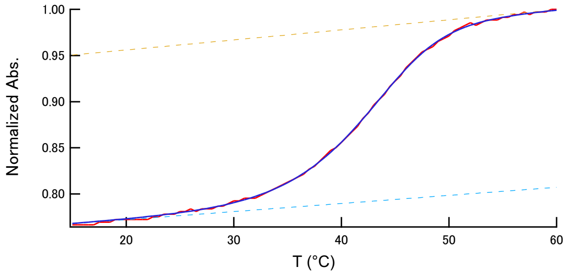

$\Delta G°_{37}$ = –8.76 kcal/mol

$\Delta H°$ = –79.60 kcal/mol

$\Delta S°$ = –228.42 cal/mol·K

T_m = 42.35°C

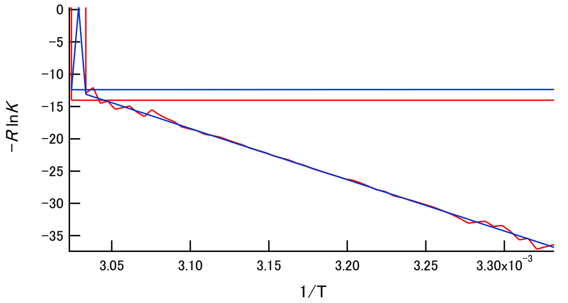

$\Delta G°_{37vh}$ = –8.77 kcal/mol

$\Delta H°_{vh}$ = –79.43 kcal/mol

$\Delta S°_{vh}$ = –227.83 cal/mol·K

$T_{m\ vh}$ = 42.39°C

ii(4/RNA) C_t = 12 μM

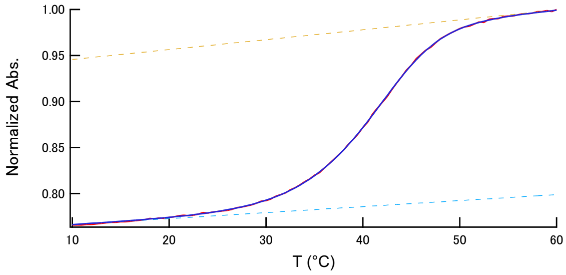

$\Delta G°_{37}$ = –8.79 kcal/mol

$\Delta H°$ = –78.72 kcal/mol

$\Delta S°$ = –225.48 cal/mol·K

T_m = 40.79°C

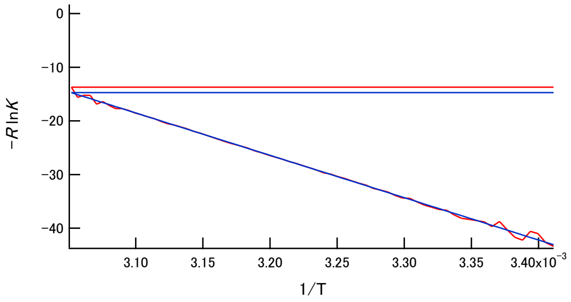

$\Delta G°_{37vh}$ = –8.79 kcal/mol

$\Delta H°_{vh}$ = –78.89 kcal/mol

$\Delta S°_{vh}$ = –226.04 cal/mol·K

$T_{m\ vh}$ = 40.77°C

ii(4/RNA) C_t = 7.5 μM

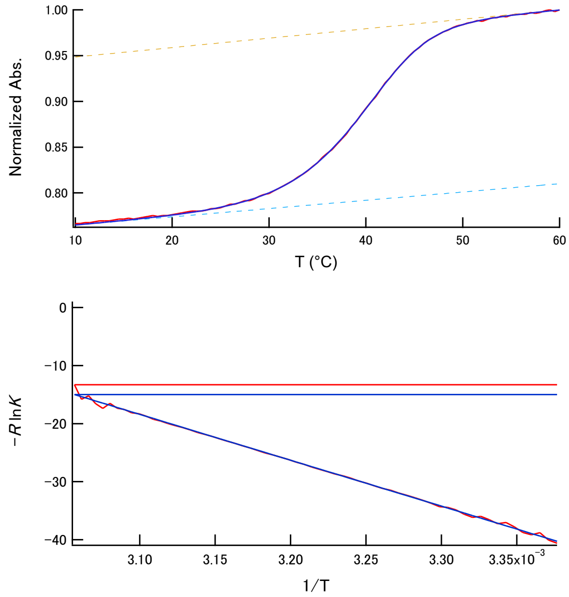

ΔG°_{37} = –8.76 kcal/mol

ΔH° = –78.62 kcal/mol

ΔS° = –225.27 cal/mol·K

T_m = 39.51°C

ΔG°_{37vh} = –8.75 kcal/mol

ΔH°_{vh} = –79.05 kcal/mol

ΔS°_{vh} = –226.64 cal/mol·K

$T_{m\ vh}$ = 39.48°C

ii(4/RNA) C_t = 6 μM

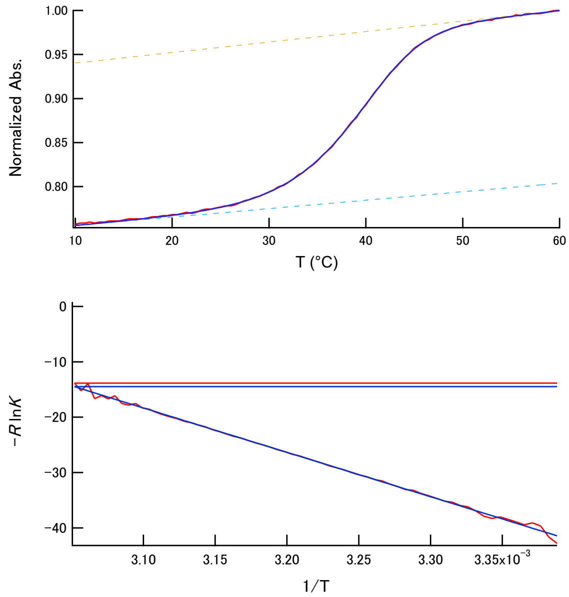

ΔG°_{37} = –8.78 kcal/mol

ΔH° = –79.69 kcal/mol

ΔS° = –228.65 cal/mol·K

T_m = 39.01°C

ΔG°_{37vh} = –8.78 kcal/mol

ΔH°_{vh} = –80.17 kcal/mol

ΔS°_{vh} = –230.20 cal/mol·K

$T_{m\ vh}$ = 39.00°C

ii(5/RNA) C_t = 30 μM

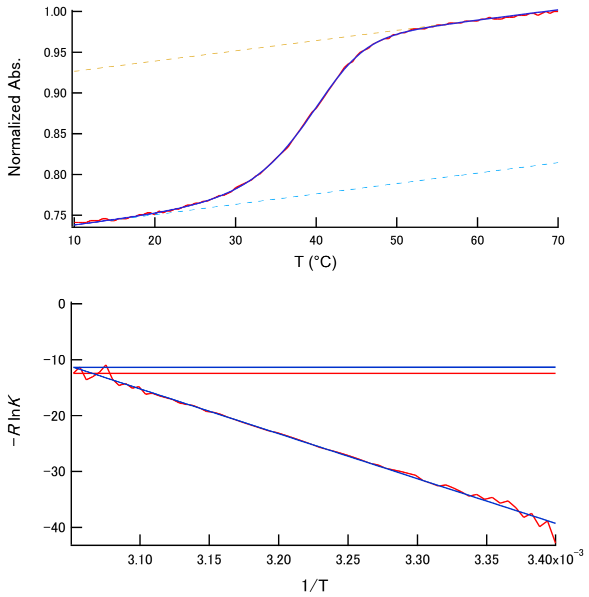

$\Delta G°_{37}$ = –7.79 kcal/mol

$\Delta H°$ = –78.94 kcal/mol

$\Delta S°$ = –229.39 cal/mol·K

T_m = 39.07°C

$\Delta G°_{37vh}$ = –7.81 kcal/mol

$\Delta H°_{vh}$ = –80.39 kcal/mol

$\Delta S°_{vh}$ = –234.01 cal/mol·K

$T_{m\ vh}$ = 39.10°C

ii(5/RNA) C_t = 24 μM

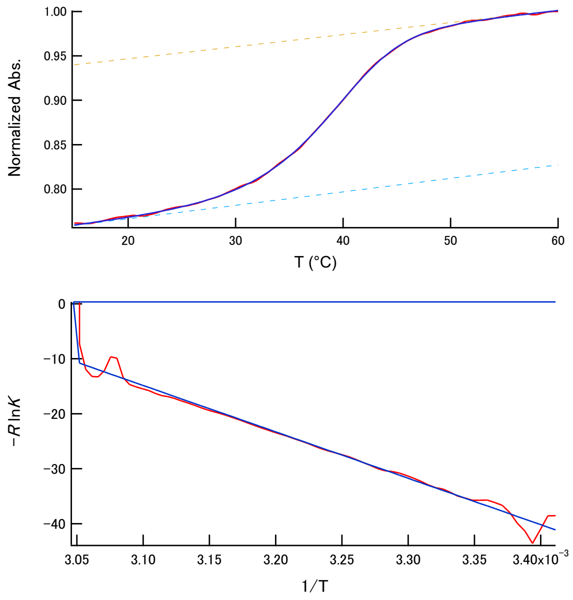

$\Delta G°_{37}$ = –7.88 kcal/mol

$\Delta H°$ = –81.79 kcal/mol

$\Delta S°$ = –238.31 cal/mol·K

T_m = 38.79°C

$\Delta G°_{37vh}$ = –7.86 kcal/mol

$\Delta H°_{vh}$ = –84.43 kcal/mol

$\Delta S°_{vh}$ = –246.88 cal/mol·K

$T_{m\ vh}$ = 38.66°C

ii(5/RNA) C_t = 12 μM

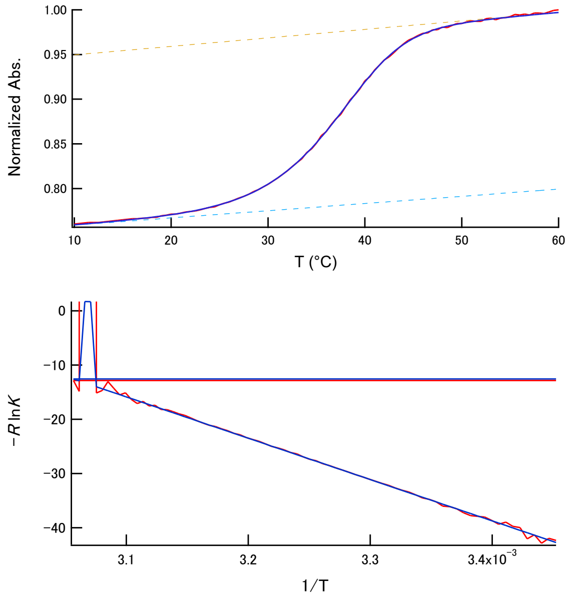

$\Delta G°_{37}$ = –7.85 kcal/mol

$\Delta H°$ = –76.63 kcal/mol

$\Delta S°$ = –221.76 cal/mol·K

T_m = 37.04°C

$\Delta G°_{37vh}$ = –7.86 kcal/mol

$\Delta H°_{vh}$ = –76.22 kcal/mol

$\Delta S°_{vh}$ = –220.40 cal/mol·K

$T_{m\ vh}$ = 37.10°C

ii(5/RNA) C_t = 7.5 μM

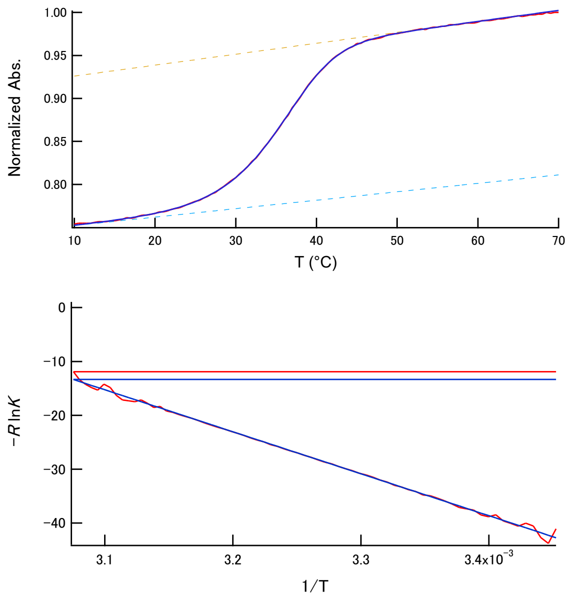

$\Delta G°_{37}$ = –7.75 kcal/mol

$\Delta H°$ = –77.46 kcal/mol

$\Delta S°$ = –224.77 cal/mol·K

T_m = 35.49°C

$\Delta G°_{37vh}$ = –7.73 kcal/mol

$\Delta H°_{vh}$ = –77.94 kcal/mol

$\Delta S°_{vh}$ = –226.34 cal/mol·K

$T_{m\ vh}$ = 35.45°C

ii(5/RNA) C_t = 6 µM

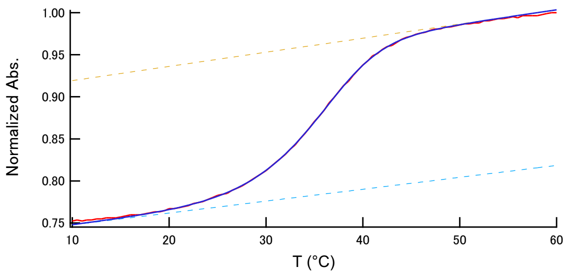

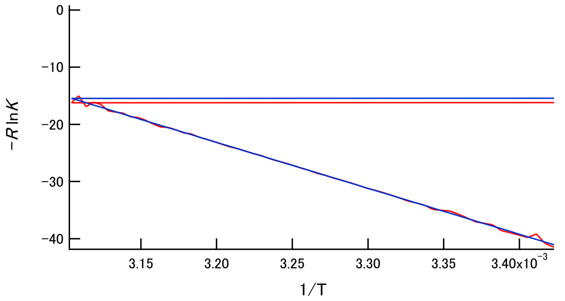

$\Delta G°_{37}$ = –7.79 kcal/mol

$\Delta H°$ = –80.18 kcal/mol

$\Delta S°$ = –233.41 cal/mol·K

T_m = 35.17°C

$\Delta G°_{37vh}$ = –7.79 kcal/mol

$\Delta H°_{vh}$ = –80.37 kcal/mol

$\Delta S°_{vh}$ = –234.03 cal/mol·K

$T_{m\ vh}$ = 35.16°C

ii(6/RNA) C_t = 30 µM

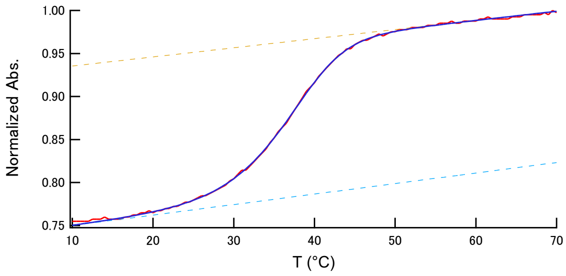

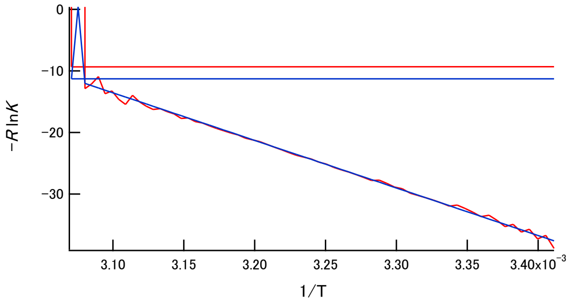

$\Delta G°_{37}$ = –7.20 kcal/mol

$\Delta H°$ = –76.17 kcal/mol

$\Delta S°$ = –222.38 cal/mol·K

T_m = 36.69°C

$\Delta G°_{37vh}$ = –7.18 kcal/mol

$\Delta H°_{vh}$ = –77.35 kcal/mol

$\Delta S°_{vh}$ = –226.23 cal/mol·K

$T_{m\ vh}$ = 36.64°C

ii(6/RNA) C_t = 24 μM

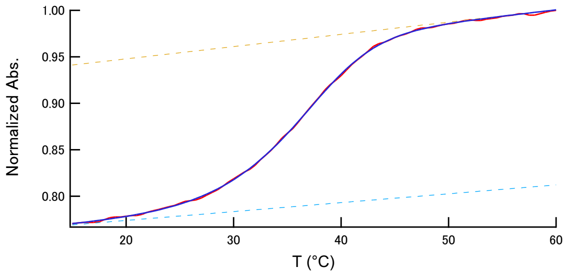

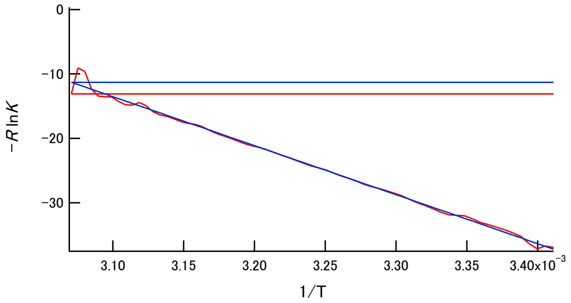

$\Delta G°_{37}$ = –7.14 kcal/mol

$\Delta H°$ = –75.10 kcal/mol

$\Delta S°$ = –219.09 cal/mol·K

T_m = 35.91°C

$\Delta G°_{37vh}$ = –7.13 kcal/mol

$\Delta H°_{vh}$ = –76.09 kcal/mol

$\Delta S°_{vh}$ = –222.37 cal/mol·K

$T_{m\ vh}$ = 35.85°C

ii(6/RNA) C_t = 12 μM

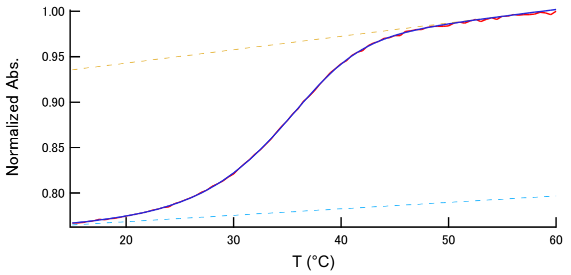

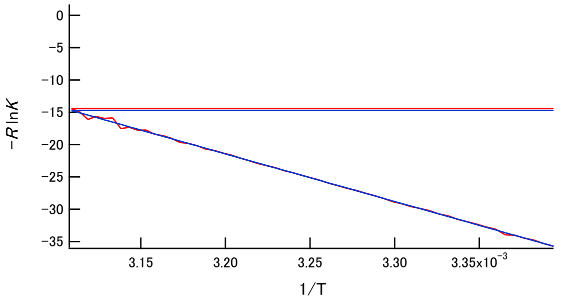

$\Delta G°_{37}$ = –7.21 kcal/mol

$\Delta H°$ = –73.61 kcal/mol

$\Delta S°$ = –214.11 cal/mol·K

T_m = 34.37°C

$\Delta G°_{37vh}$ = –7.20 kcal/mol

$\Delta H°_{vh}$ = –73.76 kcal/mol

$\Delta S°_{vh}$ = –214.61 cal/mol·K

$T_{m\ vh}$ = 34.36°C

ii(6/RNA) C_t = 7.5 μM

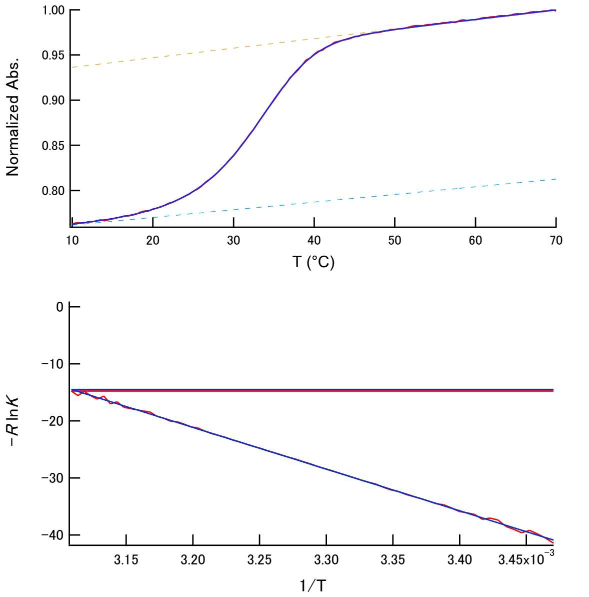

ΔG°_{37} = –7.10 kcal/mol

ΔH° = –73.14 kcal/mol

ΔS° = –212.92 cal/mol·K

T_m = 32.71°C

ΔG°_{37vh} = –7.11 kcal/mol

ΔH°_{vh} = –73.06 kcal/mol

ΔS°_{vh} = –212.64 cal/mol·K

$T_{m\ vh}$ = 32.72°C

ii(6/RNA) C_t = 6 μM

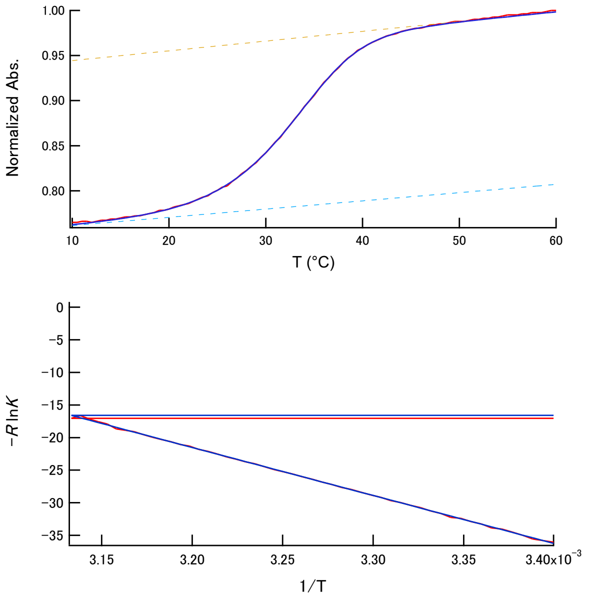

ΔG°_{37} = –7.23 kcal/mol

ΔH° = –73.69 kcal/mol

ΔS° = –214.30 cal/mol·K

T_m = 32.70°C

ΔG°_{37vh} = –7.23 kcal/mol

ΔH°_{vh} = –73.73 kcal/mol

ΔS°_{vh} = –214.41 cal/mol·K

$T_{m\ vh}$ = 32.70°C

iii(DNA/RNA) C_t = 30 μM

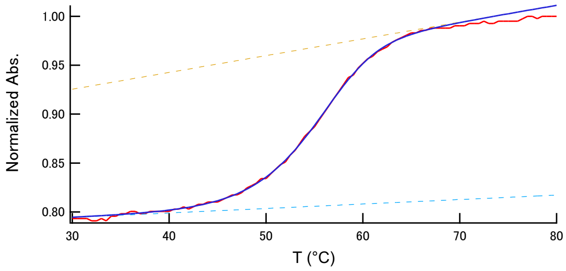

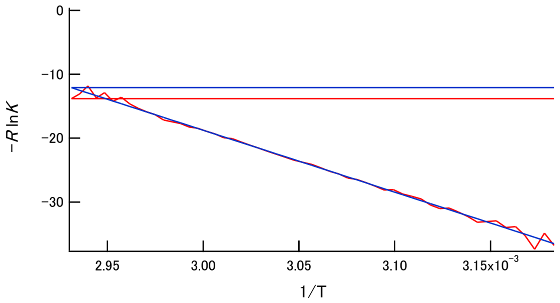

$\Delta G°_{37}$ = –12.53 kcal/mol

$\Delta H°$ = –96.46 kcal/mol

$\Delta S°$ = –270.61 cal/mol·K

T_m = 54.89°C

$\Delta G°_{37vh}$ = –12.56 kcal/mol

$\Delta H°_{vh}$ = –96.93 kcal/mol

$\Delta S°_{vh}$ = –272.05 cal/mol·K

$T_{m\ vh}$ = 54.89°C

iii(DNA/RNA) C_t = 24 μM

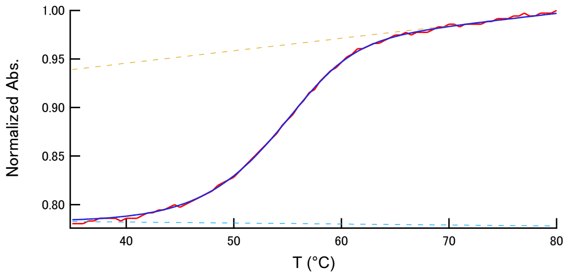

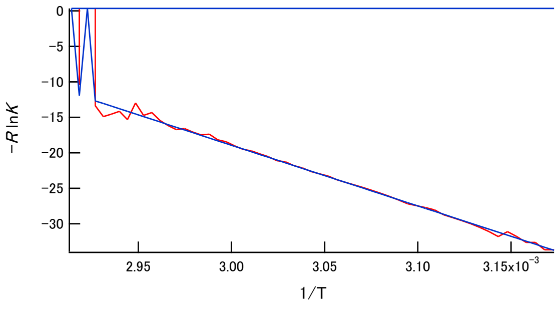

$\Delta G°_{37}$ = –11.86 kcal/mol

$\Delta H°$ = –86.47 kcal/mol

$\Delta S°$ = –240.56 cal/mol·K

T_m = 53.82°C

$\Delta G°_{37vh}$ = –11.82 kcal/mol

$\Delta H°_{vh}$ = –85.55 kcal/mol

$\Delta S°_{vh}$ = –237.71 cal/mol·K

$T_{m\ vh}$ = 53.86°C

iii(DNA/RNA) C_t = 15 μM

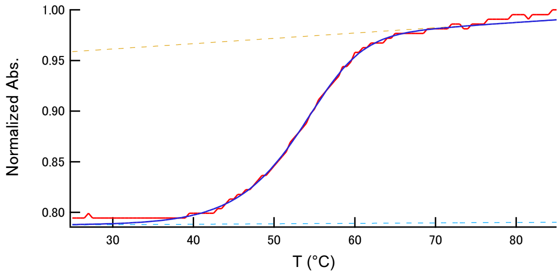

ΔG°_{37} = –11.68 kcal/mol

ΔH° = –80.05 kcal/mol

ΔS° = –220.45 cal/mol·K

T_m = 53.23°C

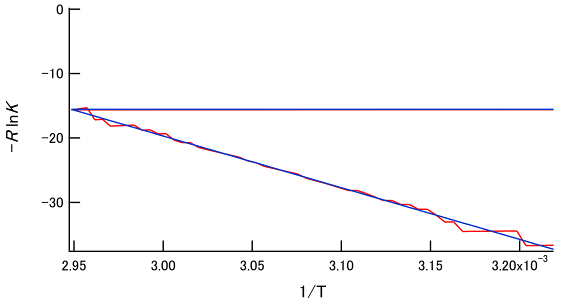

ΔG°_{37vh} = –11.69 kcal/mol

ΔH°_{vh} = –80.30 kcal/mol

ΔS°_{vh} = –221.21 cal/mol·K

$T_{m\ vh}$ = 53.23°C

iii(DNA/RNA) C_t = 12 μM

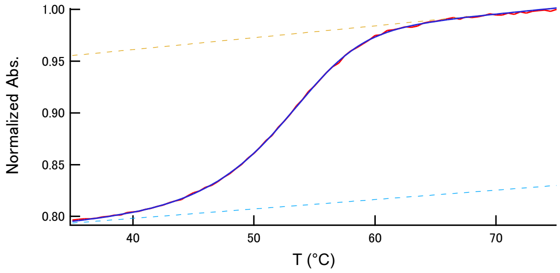

ΔG°_{37} = –12.38 kcal/mol

ΔH° = –95.17 kcal/mol

ΔS° = –266.93 cal/mol·K

T_m = 52.53°C

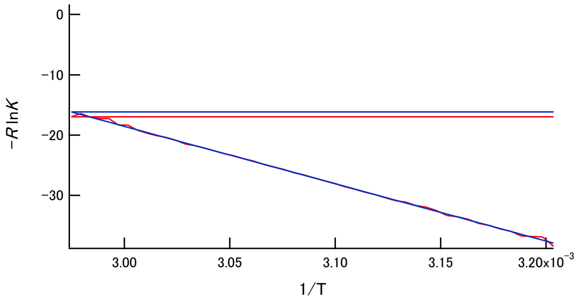

ΔG°_{37vh} = –12.38 kcal/mol

ΔH°_{vh} = –95.21 kcal/mol

ΔS°_{vh} = –267.06 cal/mol·K

$T_{m\ vh}$ = 52.53°C

iii(DNA/RNA) C_t = 7.5 μM

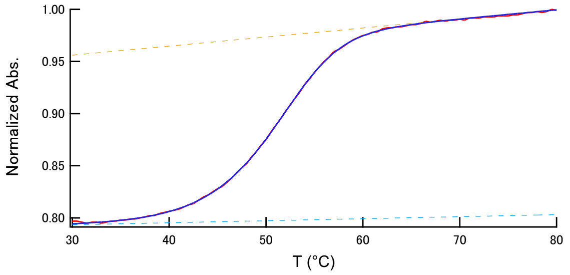

$\Delta G°_{37}$ = –11.48 kcal/mol

$\Delta H°$ = –88.65 kcal/mol

$\Delta S°$ = –248.84 cal/mol·K

T_m = 50.81°C

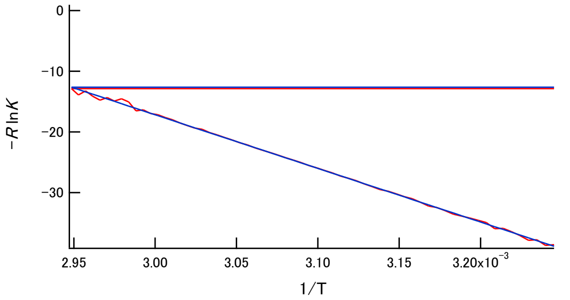

$\Delta G°_{37vh}$ = –11.47 kcal/mol

$\Delta H°_{vh}$ = –88.36 kcal/mol

$\Delta S°_{vh}$ = –247.91 cal/mol·K

$T_{m\ vh}$ = 50.83°C

iii(DNA/RNA) C_t = 6 μM

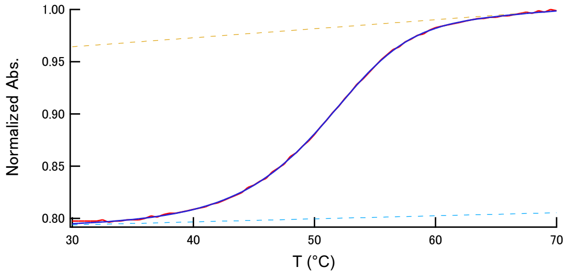

$\Delta G°_{37}$ = –11.95 kcal/mol

$\Delta H°$ = –86.70 kcal/mol

$\Delta S°$ = –241.02 cal/mol·K

T_m = 50.77°C

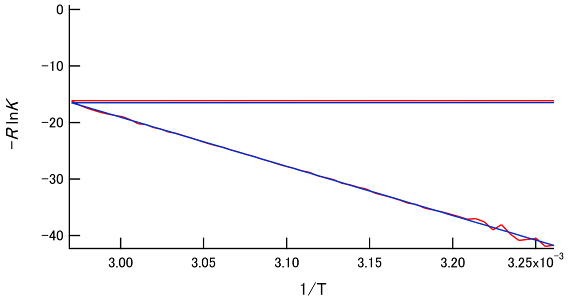

$\Delta G°_{37vh}$ = –11.96 kcal/mol

$\Delta H°_{vh}$ = –86.99 kcal/mol

$\Delta S°_{vh}$ = –241.89 cal/mol·K

$T_{m\ vh}$ = 50.77°C

iii(DNA/RNA) C_t = 3.75 μM

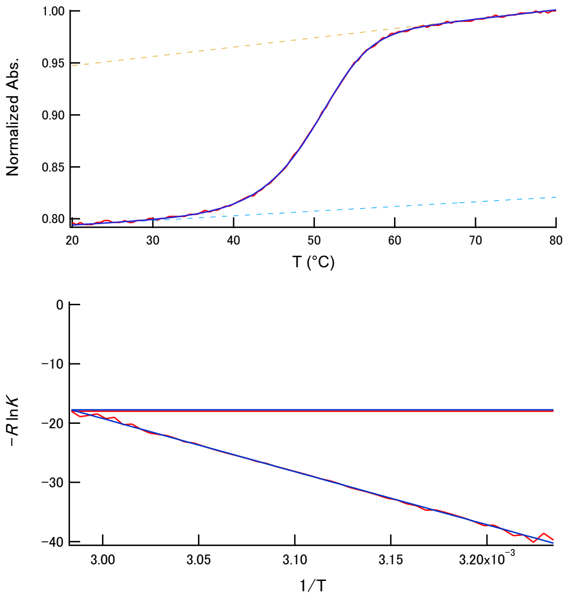

$\Delta G°_{37}$ = −12.21 kcal/mol

$\Delta H°$ = −90.00 kcal/mol

$\Delta S°$ = −250.80 cal/mol·K

T_m = 50.15°C

$\Delta G°_{37vh}$ = −12.20 kcal/mol

$\Delta H°_{vh}$ = −89.65 kcal/mol

$\Delta S°_{vh}$ = −249.73 cal/mol·K

$T_{m\ vh}$ = 50.14°C

iii(1/RNA) C_t = 30 μM

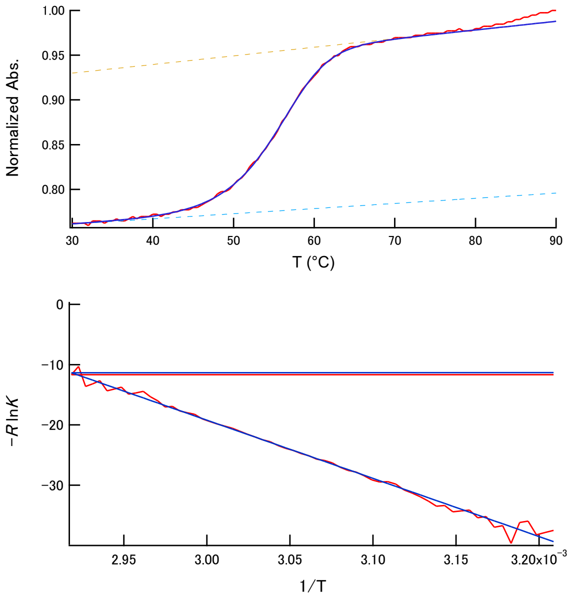

$\Delta G°_{37}$ = −12.73 kcal/mol

$\Delta H°$ = −97.65 kcal/mol

$\Delta S°$ = −273.80 cal/mol·K

T_m = 55.37°C

$\Delta G°_{37vh}$ = −12.67 kcal/mol

$\Delta H°_{vh}$ = −96.46 kcal/mol

$\Delta S°_{vh}$ = −270.18 cal/mol·K

$T_{m\ vh}$ = 55.38°C

iii(1/RNA) C_t = 24 μM

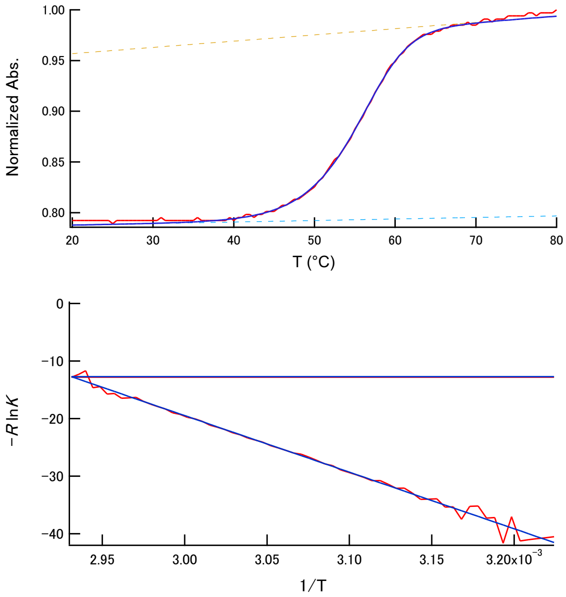

$\Delta G°_{37}$ = −12.80 kcal/mol

$\Delta H°$ = −96.81 kcal/mol

$\Delta S°$ = −270.87 cal/mol·K

T_m = 55.29°C

$\Delta G°_{37vh}$ = −12.88 kcal/mol

$\Delta H°_{vh}$ = −98.27 kcal/mol

$\Delta S°_{vh}$ = −275.33 cal/mol·K

$T_{m\ vh}$ = 55.27°C

iii(1/RNA) C_t = 12 μM

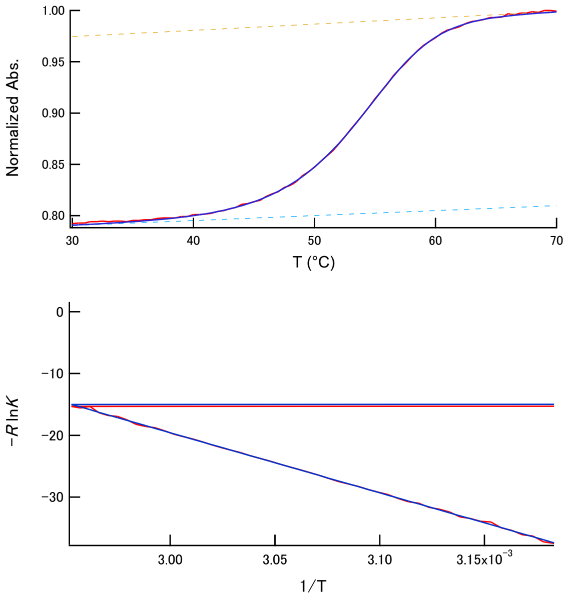

$\Delta G°_{37}$ = −12.83 kcal/mol

$\Delta H°$ = −97.11 kcal/mol

$\Delta S°$ = −271.75 cal/mol·K

T_m = 53.80°C

$\Delta G°_{37vh}$ = −12.83 kcal/mol

$\Delta H°_{vh}$ = −97.21 kcal/mol

$\Delta S°_{vh}$ = −272.05 cal/mol·K

$T_{m\ vh}$ = 53.81°C

iii(1/RNA) C_t = 7.5 μM

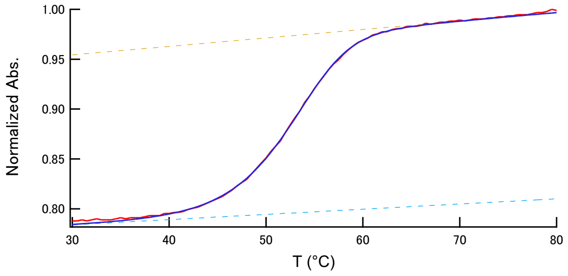

$\Delta G°_{37}$ = –12.85 kcal/mol

$\Delta H°$ = –98.87 kcal/mol

$\Delta S°$ = –277.33 cal/mol·K

T_m = 52.57°C

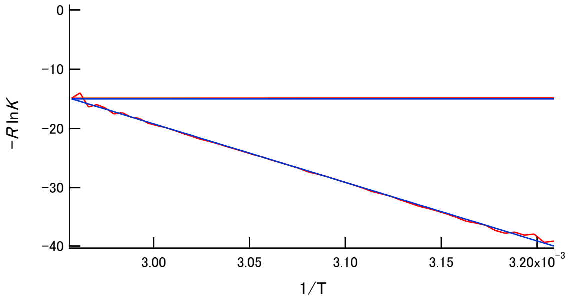

$\Delta G°_{37vh}$ = –12.84 kcal/mol

$\Delta H°_{vh}$ = –98.87 kcal/mol

$\Delta S°_{vh}$ = –277.39 cal/mol·K

$T_{m\ vh}$ = 52.51°C

iii(1/RNA) C_t = 6 μM

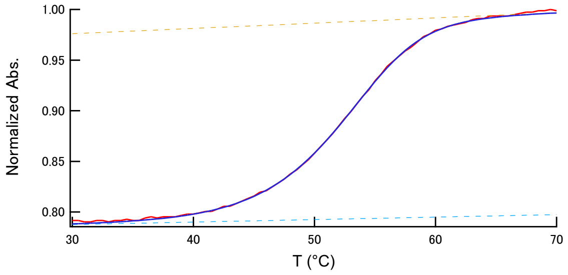

$\Delta G°_{37}$ = –12.63 kcal/mol

$\Delta H°$ = –92.40 kcal/mol

$\Delta S°$ = –257.17 cal/mol·K

T_m = 52.40°C

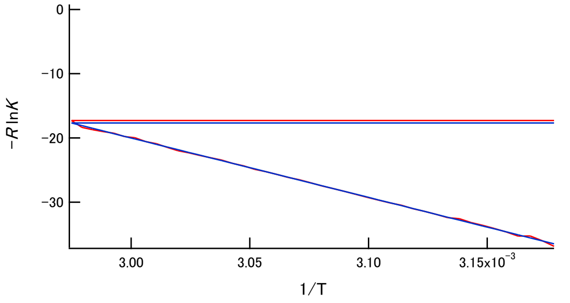

$\Delta G°_{37vh}$ = –12.64 kcal/mol

$\Delta H°_{vh}$ = –92.52 kcal/mol

$\Delta S°_{vh}$ = –257.55 cal/mol·K

$T_{m\ vh}$ = 52.40°C

iii(2/RNA) C_t = 30 μM

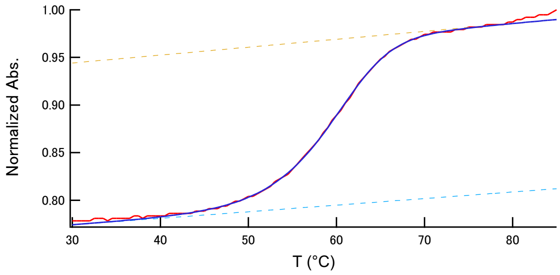

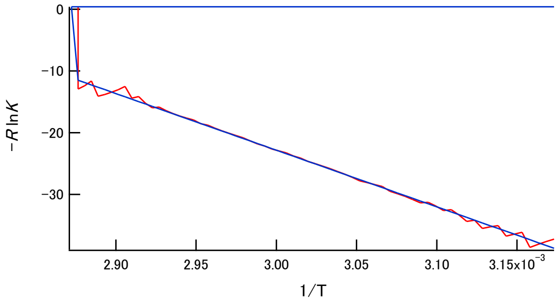

$\Delta G°_{37}$ = –13.52 kcal/mol

$\Delta H°$ = –92.65 kcal/mol

$\Delta S°$ = –255.14 cal/mol·K

T_m = 59.42°C

$\Delta G°_{37vh}$ = –13.47 kcal/mol

$\Delta H°_{vh}$ = –91.77 kcal/mol

$\Delta S°_{vh}$ = –252.46 cal/mol·K

$T_{m\ vh}$ = 59.45°C

iii(2/RNA) C_t = 24 μM

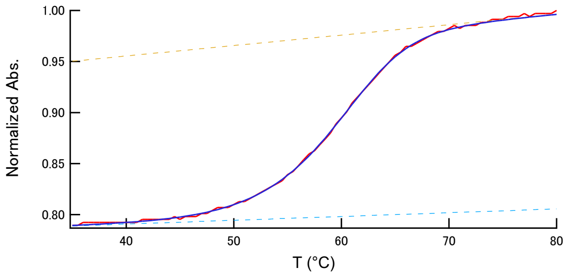

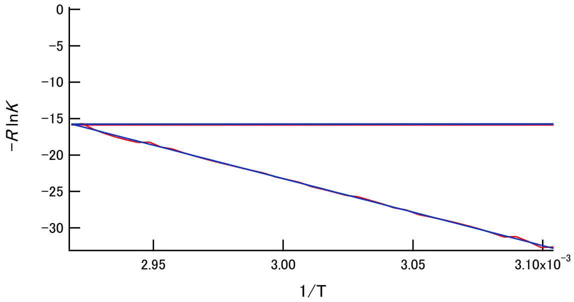

$\Delta G°_{37}$ = –13.60 kcal/mol

$\Delta H°$ = –91.85 kcal/mol

$\Delta S°$ = –252.30 cal/mol·K

T_m = 59.40°C

$\Delta G°_{37vh}$ = –13.60 kcal/mol

$\Delta H°_{vh}$ = –91.95 kcal/mol

$\Delta S°_{vh}$ = –252.61 cal/mol·K

$T_{m\ vh}$ = 59.40°C

iii(2/RNA) C_t = 12 μM

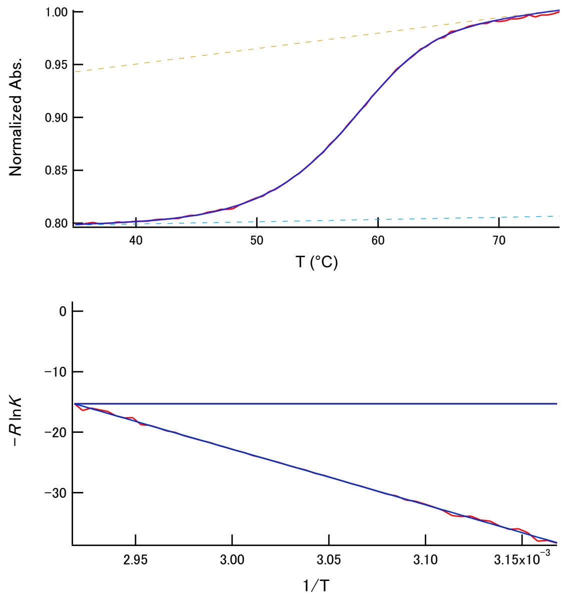

$\Delta G°_{37}$ = –13.47 kcal/mol

$\Delta H°$ = –91.90 kcal/mol

$\Delta S°$ = –252.87 cal/mol·K

T_m = 57.25°C

$\Delta G°_{37vh}$ = –13.48 kcal/mol

$\Delta H°_{vh}$ = –92.06 kcal/mol

$\Delta S°_{vh}$ = –253.37 cal/mol·K

$T_{m\ vh}$ = 57.25°C

iii(2/RNA) C_t = 7.5 μM

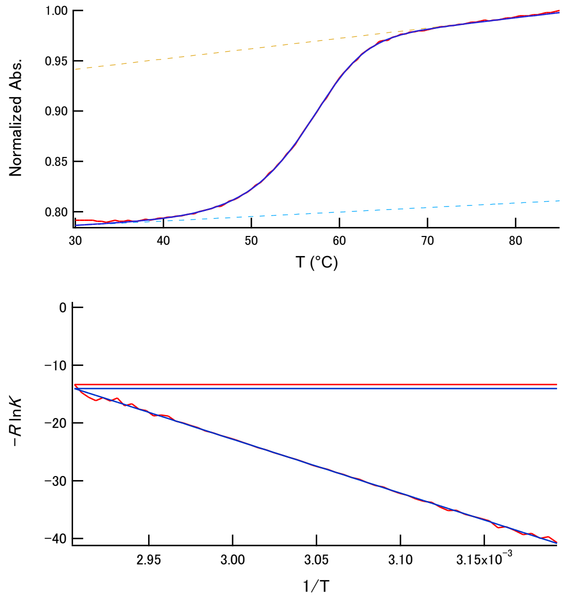

$\Delta G°_{37}$ = –13.58 kcal/mol

$\Delta H°$ = –93.54 kcal/mol

$\Delta S°$ = –257.81 cal/mol·K

T_m = 56.20°C

$\Delta G°_{37vh}$ = –13.56 kcal/mol

$\Delta H°_{vh}$ = –93.18 kcal/mol

$\Delta S°_{vh}$ = –256.70 cal/mol·K

$T_{m\ vh}$ = 56.21°C

iii(2/RNA) C_t = 6 μM

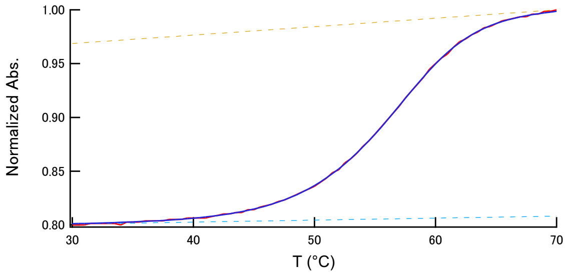

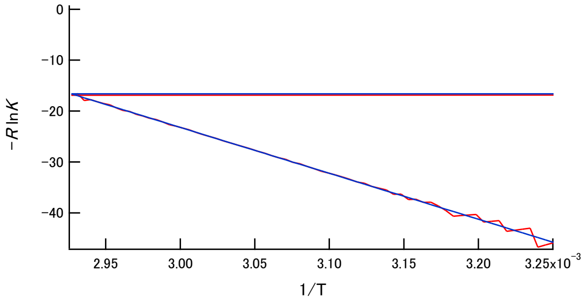

$\Delta G°_{37}$ = –13.45 kcal/mol

$\Delta H°$ = –89.90 kcal/mol

$\Delta S°$ = –246.47 cal/mol·K

T_m = 56.00°C

$\Delta G°_{37vh}$ = –13.47 kcal/mol

$\Delta H°_{vh}$ = –90.25 kcal/mol

$\Delta S°_{vh}$ = –247.53 cal/mol·K

$T_{m\ vh}$ = 56.01°C

iii(3/RNA) C_t = 30 μM

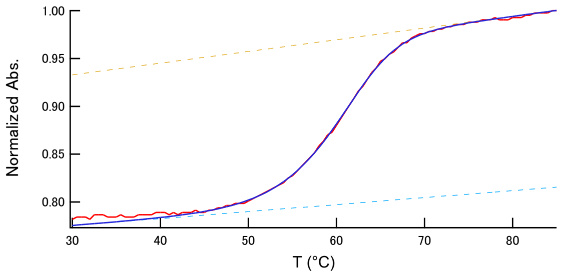

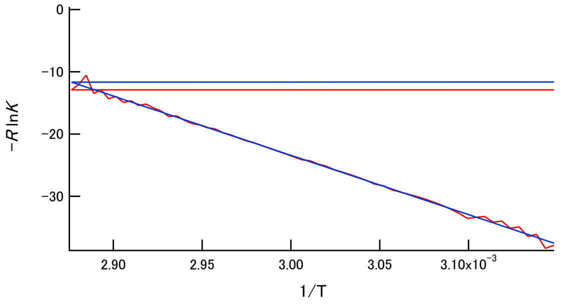

$\Delta G°_{37}$ = –13.86 kcal/mol

$\Delta H°$ = –94.78 kcal/mol

$\Delta S°$ = –260.93 cal/mol·K

T_m = 60.15°C

$\Delta G°_{37vh}$ = –13.89 kcal/mol

$\Delta H°_{vh}$ = –95.27 kcal/mol

$\Delta S°_{vh}$ = –262.37 cal/mol·K

$T_{m\ vh}$ = 60.16°C

iii(3/RNA) C_t = 24 μM

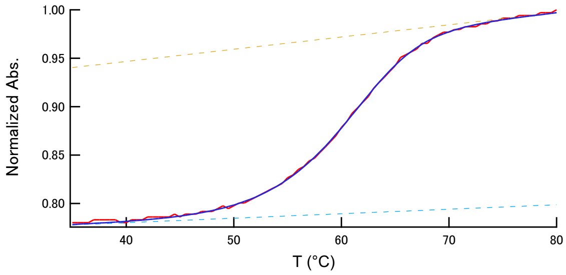

$\Delta G°_{37}$ = –13.72 kcal/mol

$\Delta H°$ = –90.55 kcal/mol

$\Delta S°$ = –247.73 cal/mol·K

T_m = 60.22°C

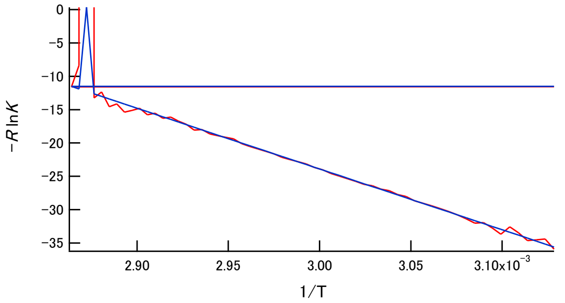

$\Delta G°_{37vh}$ = –13.75 kcal/mol

$\Delta H°_{vh}$ = –91.13 kcal/mol

$\Delta S°_{vh}$ = –249.48 cal/mol·K

$T_{m\ vh}$ = 60.20°C

iii(3/RNA) C_t = 12 μM

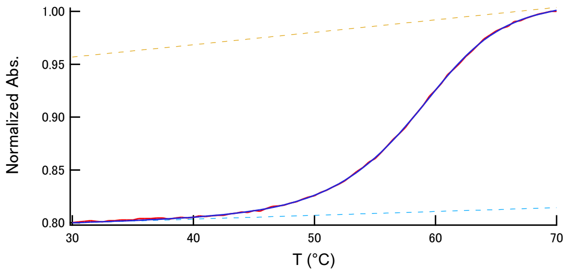

$\Delta G°_{37}$ = –13.84 kcal/mol

$\Delta H°$ = –93.96 kcal/mol

$\Delta S°$ = –258.33 cal/mol·K

T_m = 58.17°C

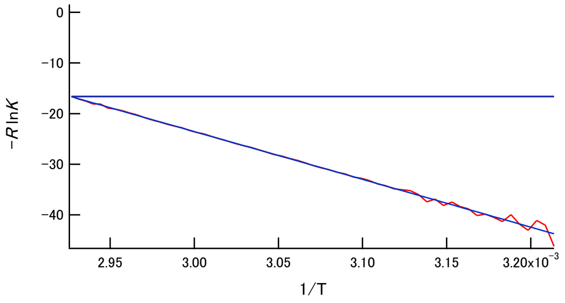

$\Delta G°_{37vh}$ = –13.87 kcal/mol

$\Delta H°_{vh}$ = –94.37 kcal/mol

$\Delta S°_{vh}$ = –259.54 cal/mol·K

$T_{m\ vh}$ = 58.18°C

iii(3/RNA) C_t = 7.5 μM

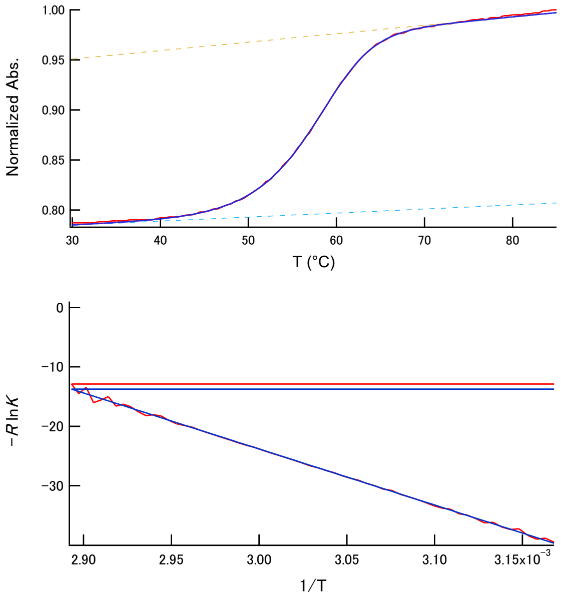

$\Delta G°_{37}$ = –13.96 kcal/mol

$\Delta H°$ = –94.26 kcal/mol

$\Delta S°$ = –258.91 cal/mol·K

T_m = 57.45°C

$\Delta G°_{37vh}$ = –13.96 kcal/mol

$\Delta H°_{vh}$ = –94.46 kcal/mol

$\Delta S°_{vh}$ = –259.55 cal/mol·K

$T_{m\ vh}$ = 57.42°C

iii(3/RNA) C_t = 6 μM

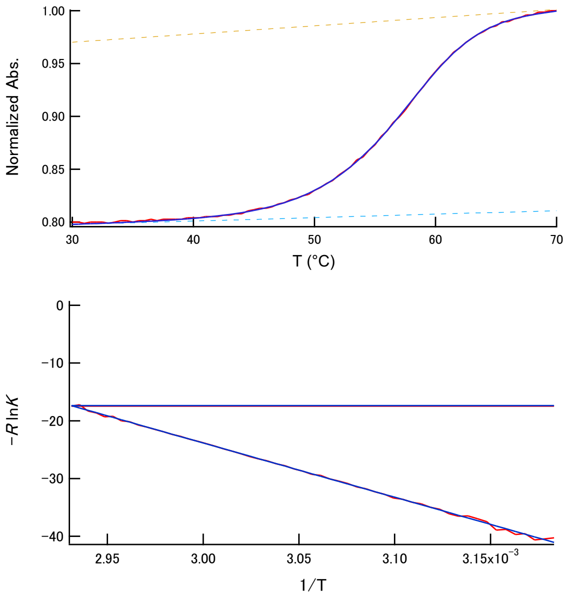

$\Delta G°_{37}$ = –13.93 kcal/mol

$\Delta H°$ = –93.95 kcal/mol

$\Delta S°$ = –258.01 cal/mol·K

T_m = 56.90°C

$\Delta G°_{37vh}$ = –13.93 kcal/mol

$\Delta H°_{vh}$ = –94.00 kcal/mol

$\Delta S°_{vh}$ = –258.16 cal/mol·K

$T_{m\ vh}$ = 56.90°C

iii(4/RNA) C_t = 30 μM

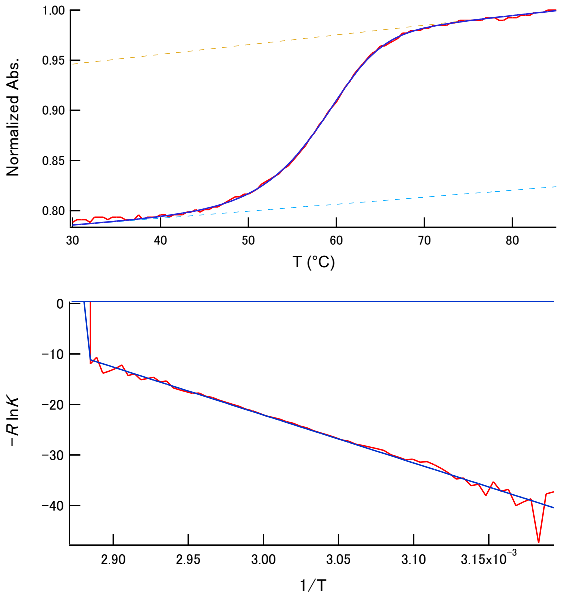

$\Delta G°_{37}$ = –13.30 kcal/mol

$\Delta H°$ = –93.26 kcal/mol

$\Delta S°$ = –257.82 cal/mol·K

T_m = 58.43°C

$\Delta G°_{37vh}$ = –13.45 kcal/mol

$\Delta H°_{vh}$ = –94.92 kcal/mol

$\Delta S°_{vh}$ = –262.69 cal/mol·K

$T_{m\ vh}$ = 58.59°C

iii(4/RNA) C_t = 24 μM

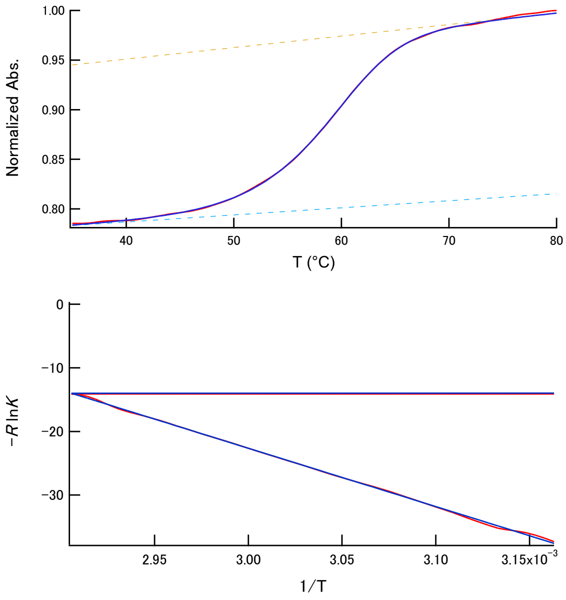

$\Delta G°_{37}$ = –13.41 kcal/mol

$\Delta H°$ = –91.78 kcal/mol

$\Delta S°$ = –252.70 cal/mol·K

T_m = 58.68°C

$\Delta G°_{37vh}$ = –13.40 kcal/mol

$\Delta H°_{vh}$ = –91.71 kcal/mol

$\Delta S°_{vh}$ = –252.49 cal/mol·K

$T_{m\ vh}$ = 58.67°C

iii(4/RNA) C_t = 12 μM

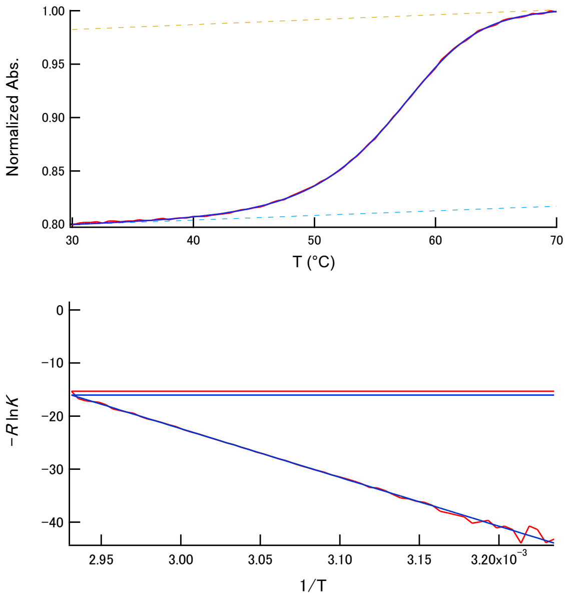

$\Delta G°_{37}$ = –13.34 kcal/mol

$\Delta H°$ = –92.06 kcal/mol

$\Delta S°$ = –253.81 cal/mol·K

T_m = 56.72°C

$\Delta G°_{37vh}$ = –13.34 kcal/mol

$\Delta H°_{vh}$ = –91.98 kcal/mol

$\Delta S°_{vh}$ = –253.57 cal/mol·K

$T_{m\ vh}$ = 56.72°C

iii(4/RNA) C_t = 7.5 μM

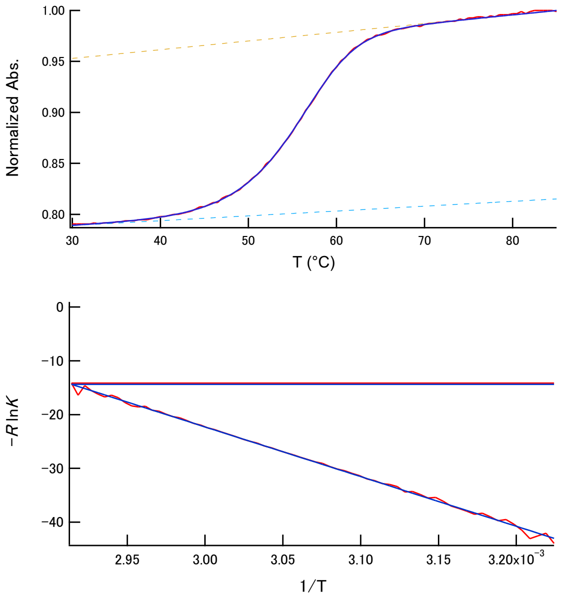

$\Delta G°_{37}$ = –13.29 kcal/mol

$\Delta H°$ = –91.84 kcal/mol

$\Delta S°$ = –253.24 cal/mol·K

T_m = 55.49°C

$\Delta G°_{37vh}$ = –13.34 kcal/mol

$\Delta H°_{vh}$ = –92.35 kcal/mol

$\Delta S°_{vh}$ = –254.78 cal/mol·K

$T_{m\ vh}$ = 55.54°C

iii(4/RNA) C_t = 6 μM

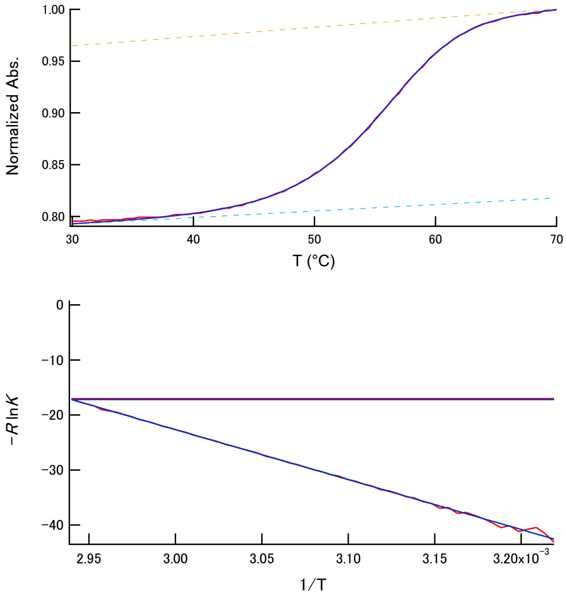

$\Delta G°_{37}$ = –13.34 kcal/mol

$\Delta H°$ = –90.75 kcal/mol

$\Delta S°$ = –249.60 cal/mol·K

T_m = 55.37°C

$\Delta G°_{37vh}$ = –13.34 kcal/mol

$\Delta H°_{vh}$ = –90.77 kcal/mol

$\Delta S°_{vh}$ = –249.64 cal/mol·K

$T_{m\ vh}$ = 55.37°C

iii(5/RNA) C_t = 30 μM

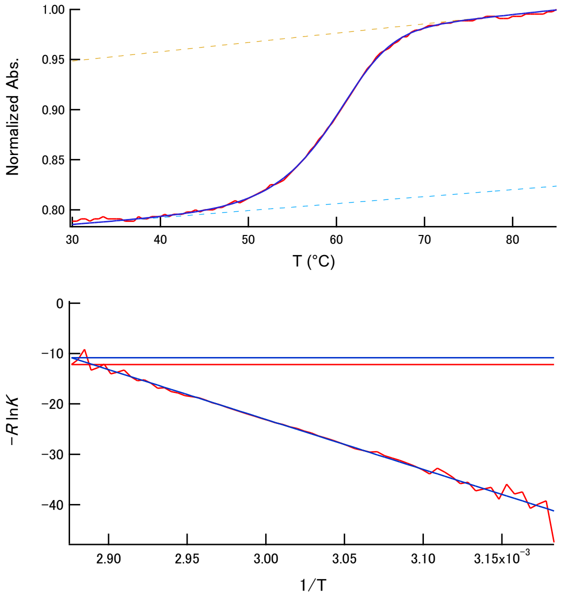

$\Delta G°_{37}$ = –13.90 kcal/mol

$\Delta H°$ = –96.81 kcal/mol

$\Delta S°$ = –267.33 cal/mol·K

T_m = 59.78°C

$\Delta G°_{37vh}$ = –14.05 kcal/mol

$\Delta H°_{vh}$ = –99.11 kcal/mol

$\Delta S°_{vh}$ = –274.24 cal/mol·K

$T_{m\ vh}$ = 59.78°C

iii(5/RNA) C_t = 24 μM

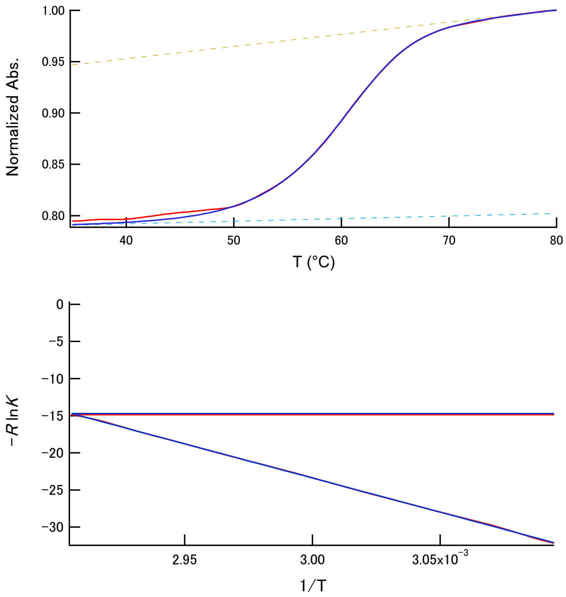

$\Delta G°_{37}$ = –13.66 kcal/mol

$\Delta H°$ = –92.18 kcal/mol

$\Delta S°$ = –253.14 cal/mol·K

T_m = 59.57°C

$\Delta G°_{37vh}$ = –13.65 kcal/mol

$\Delta H°_{vh}$ = –92.03 kcal/mol

$\Delta S°_{vh}$ = –252.70 cal/mol·K

$T_{m\ vh}$ = 59.57°C

iii(5/RNA) C_t = 12 μM

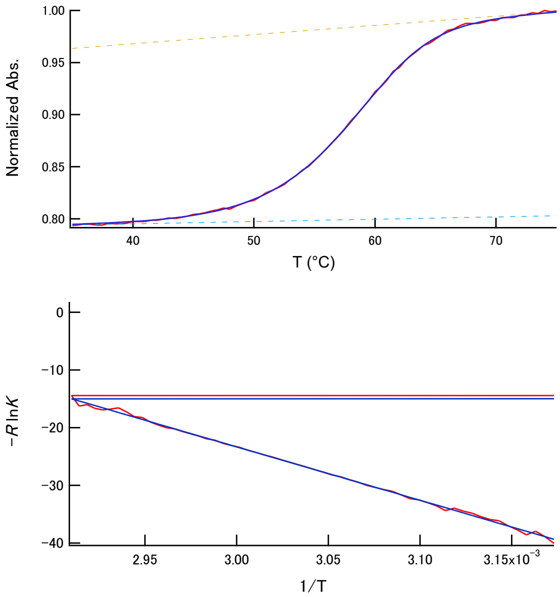

$\Delta G°_{37}$ = –13.66 kcal/mol

$\Delta H°$ = –92.33 kcal/mol

$\Delta S°$ = –253.65 cal/mol·K

T_m = 57.89°C

$\Delta G°_{37vh}$ = –13.68 kcal/mol

$\Delta H°_{vh}$ = –92.54 kcal/mol

$\Delta S°_{vh}$ = –254.27 cal/mol·K

$T_{m\ vh}$ = 57.89°C

iii(5/RNA) C_t = 7.5 μM

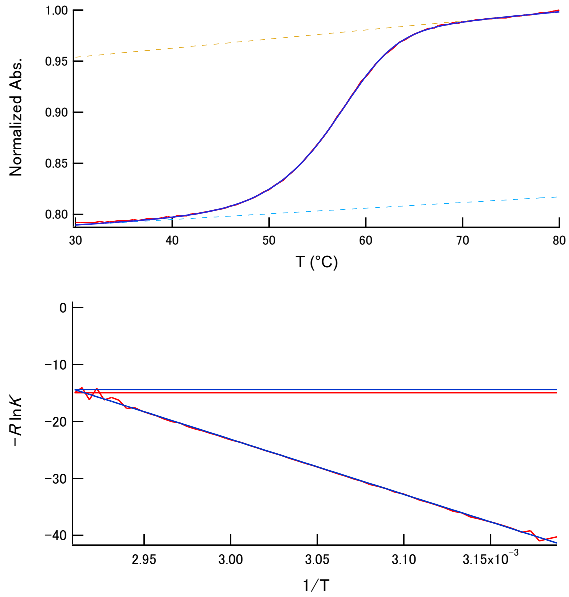

$\Delta G°_{37}$ = –13.34 kcal/mol

$\Delta H°$ = –93.89 kcal/mol

$\Delta S°$ = –259.71 cal/mol·K

T_m = 55.23°C

$\Delta G°_{37vh}$ = –13.35 kcal/mol

$\Delta H°_{vh}$ = –94.47 kcal/mol

$\Delta S°_{vh}$ = –261.54 cal/mol·K

$T_{m\ vh}$ = 55.17°C

iii(5/RNA) C_t = 6 μM

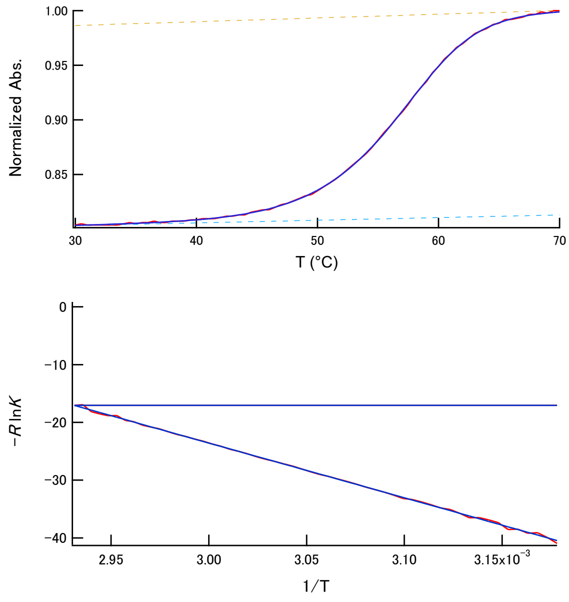

$\Delta G°_{37}$ = –13.89 kcal/mol

$\Delta H°$ = –94.60 kcal/mol

$\Delta S°$ = –260.20 cal/mol·K

T_m = 56.62°C

$\Delta G°_{37vh}$ = –13.90 kcal/mol

$\Delta H°_{vh}$ = –94.77 kcal/mol

$\Delta S°_{vh}$ = –260.74 cal/mol·K

$T_{m\ vh}$ = 56.62°C

iii(6/RNA) C_t = 30 μM

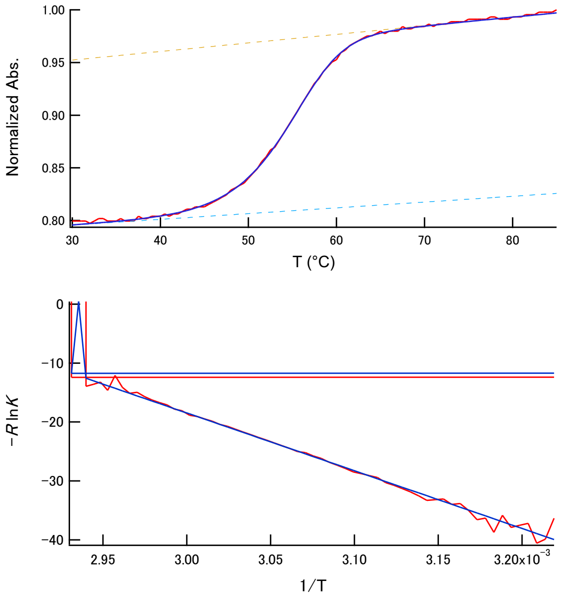

ΔG°_{37} = –12.60 kcal/mol

ΔH° = –99.27 kcal/mol

ΔS° = –279.43 cal/mol·K

T_m = 54.60°C

ΔG°_{37vh} = –12.55 kcal/mol

ΔH°_{vh} = –98.08 kcal/mol

ΔS°_{vh} = –275.76 cal/mol·K

$T_{m\ vh}$ = 54.64°C

iii(6/RNA) C_t = 24 μM

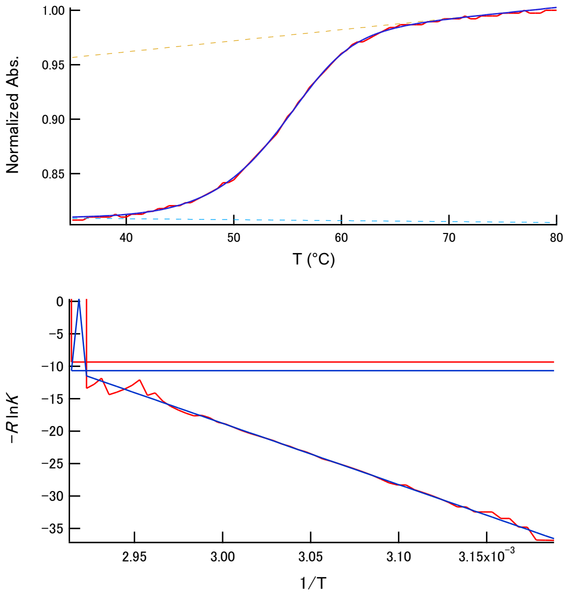

ΔG°_{37} = –12.37 kcal/mol

ΔH° = –94.01 kcal/mol

ΔS° = –263.21 cal/mol·K

T_m = 54.29°C

ΔG°_{37vh} = –12.40 kcal/mol

ΔH°_{vh} = –94.46 kcal/mol

ΔS°_{vh} = –264.58 cal/mol·K

$T_{m\ vh}$ = 54.29°C

iii(6/RNA) C_t = 12 µM

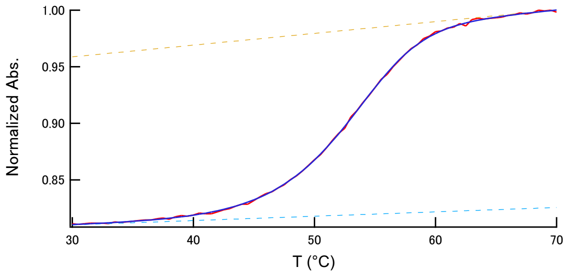

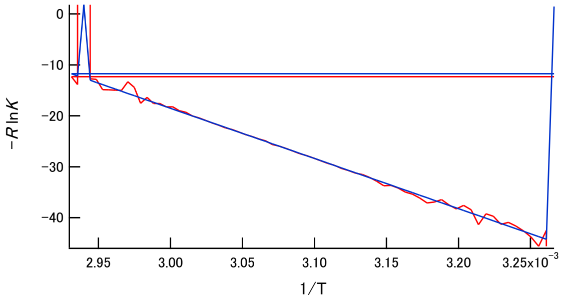

$\Delta G°_{37}$ = –12.62 kcal/mol

$\Delta H°$ = –99.09 kcal/mol

$\Delta S°$ = –278.78 cal/mol·K

T_m = 52.75°C

$\Delta G°_{37vh}$ = –12.60 kcal/mol

$\Delta H°_{vh}$ = –98.58 kcal/mol

$\Delta S°_{vh}$ = –277.22 cal/mol·K

$T_{m\ vh}$ = 52.75°C

iii(6/RNA) C_t = 7.5 µM

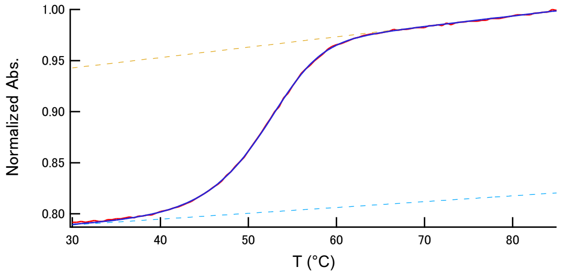

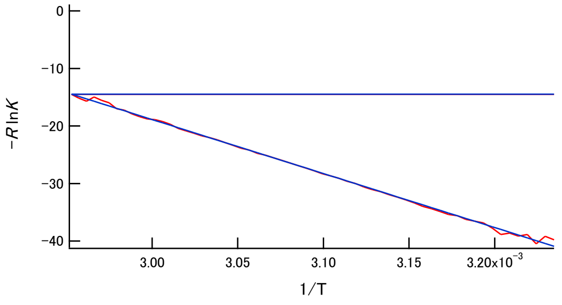

$\Delta G°_{37}$ = –12.41 kcal/mol

$\Delta H°$ = –94.25 kcal/mol

$\Delta S°$ = –263.85 cal/mol·K

T_m = 51.77°C

$\Delta G°_{37vh}$ = –12.39 kcal/mol

$\Delta H°_{vh}$ = –93.89 kcal/mol

$\Delta S°_{vh}$ = –262.79 cal/mol·K

$T_{m\ vh}$ = 51.74°C

iii(6/RNA) C_t = 6 μM

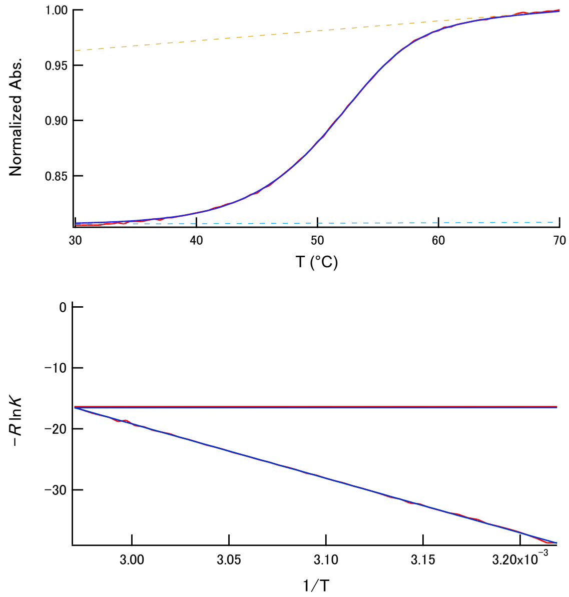

$\Delta G°_{37}$ = –12.16 kcal/mol

$\Delta H°$ = –89.42 kcal/mol

$\Delta S°$ = –249.08 cal/mol·K

T_m = 51.14°C

$\Delta G°_{37vh}$ = –12.17 kcal/mol

$\Delta H°_{vh}$ = –89.51 kcal/mol

$\Delta S°_{vh}$ = –249.36 cal/mol·K

$T_{m\,vh}$ = 51.14°C

iv(DNA/RNA) C_t = 30 μM

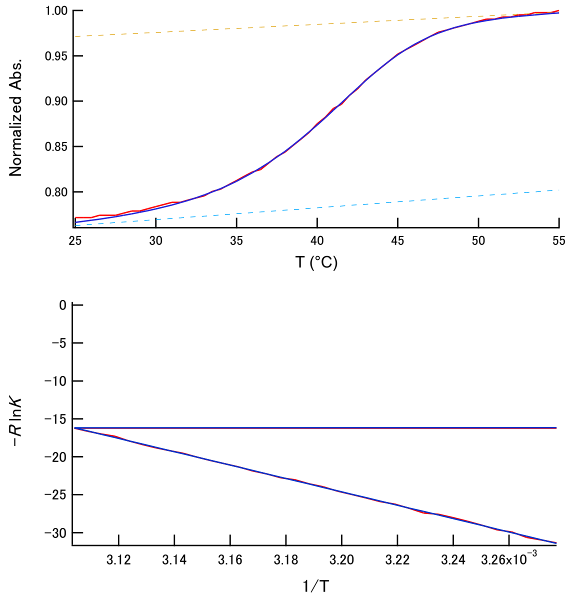

$\Delta G°_{37}$ = –8.30 kcal/mol

$\Delta H°$ = –88.15 kcal/mol

$\Delta S°$ = –257.47 cal/mol·K

T_m = 40.66°C

$\Delta G°_{37vh}$ = –8.30 kcal/mol

$\Delta H°_{vh}$ = –88.14 kcal/mol

$\Delta S°_{vh}$ = –257.41 cal/mol·K

$T_{m\,vh}$ = 40.66°C

iv(DNA/RNA) C_t = 24 µM

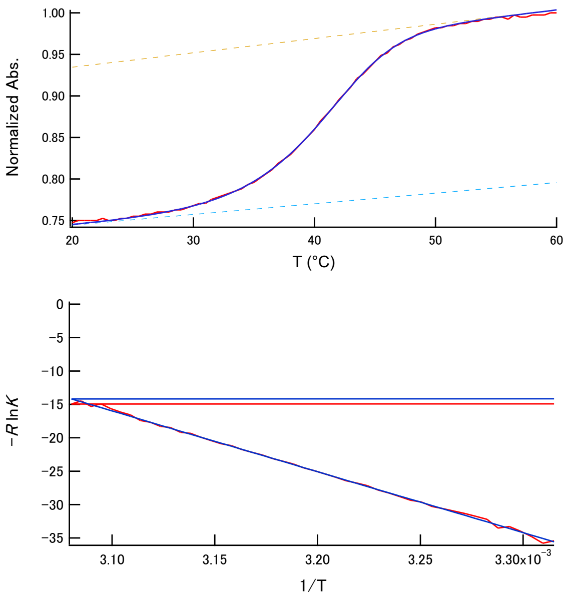

ΔG°_{37} = –8.46 kcal/mol

ΔH° = –90.87 kcal/mol

ΔS° = –265.72 cal/mol·K

T_m = 40.62°C

ΔG°_{37vh} = –8.46 kcal/mol

ΔH°_{vh} = –91.03 kcal/mol

ΔS°_{vh} = –266.22 cal/mol·K

$T_{m\ vh}$ = 40.63°C

iv(DNA/RNA) C_t = 15 µM

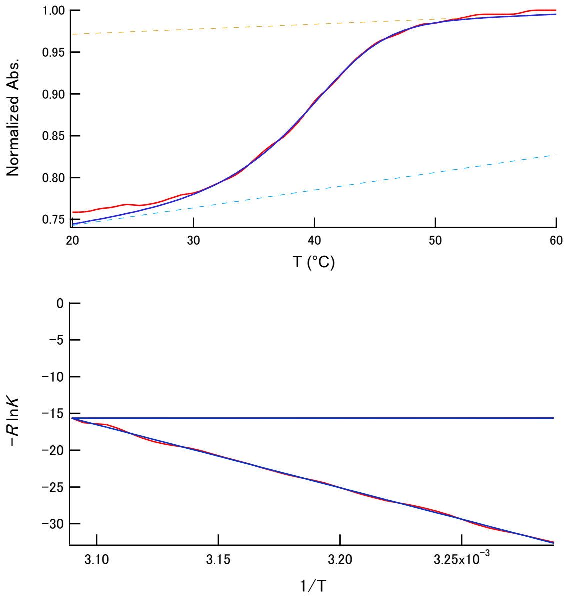

ΔG°_{37} = –8.43 kcal/mol

ΔH° = –85.69 kcal/mol

ΔS° = –249.11 cal/mol·K

T_m = 39.67°C

ΔG°_{37vh} = –8.43 kcal/mol

ΔH°_{vh} = –85.80 kcal/mol

ΔS°_{vh} = –249.47 cal/mol·K

$T_{m\ vh}$ = 39.66°C

iv(DNA/RNA) C_t = 12 µM

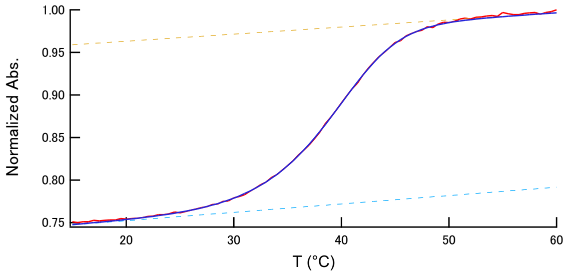

$\Delta G°_{37}$ = –8.43 kcal/mol

$\Delta H°$ = –88.44 kcal/mol

$\Delta S°$ = –257.97 cal/mol·K

T_m = 39.11°C

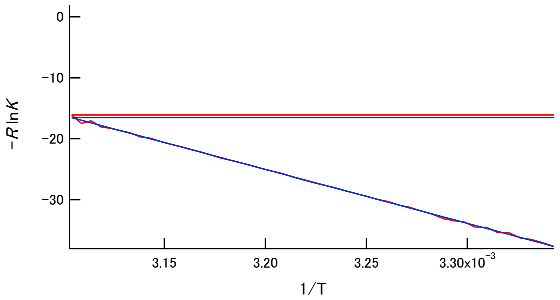

$\Delta G°_{37vh}$ = –8.43 kcal/mol

$\Delta H°_{vh}$ = –88.51 kcal/mol

$\Delta S°_{vh}$ = –258.18 cal/mol·K

$T_{m\ vh}$ = 39.10°C

iv(DNA/RNA) C_t = 7.5 µM

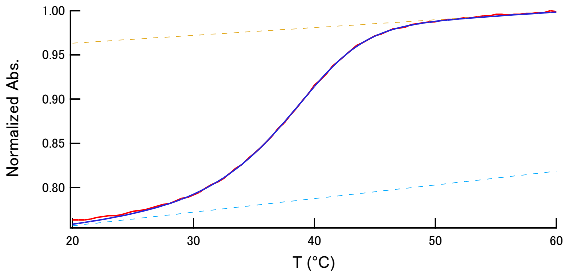

$\Delta G°_{37}$ = –8.41 kcal/mol

$\Delta H°$ = –88.44 kcal/mol

$\Delta S°$ = –258.04 cal/mol·K

T_m = 37.99°C

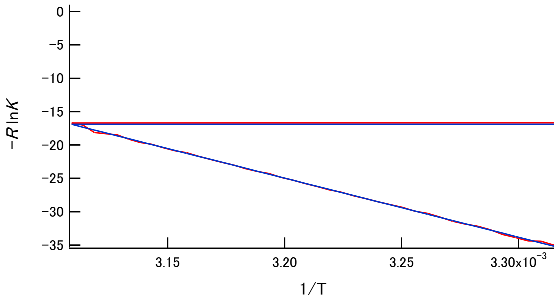

$\Delta G°_{37vh}$ = –8.41 kcal/mol

$\Delta H°_{vh}$ = –88.54 kcal/mol

$\Delta S°_{vh}$ = –258.37 cal/mol·K

$T_{m\ vh}$ = 37.99°C

iv(DNA/RNA) C_t = 6 µM

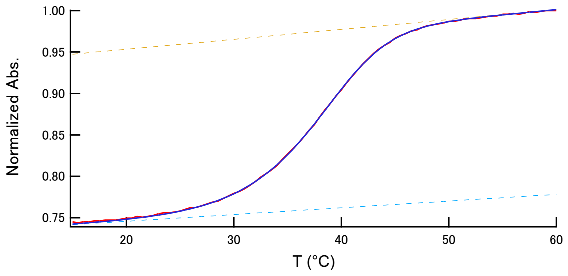

ΔG°_{37} = –8.46 kcal/mol

ΔH° = –83.23 kcal/mol

ΔS° = –241.05 cal/mol·K

T_m = 37.75°C

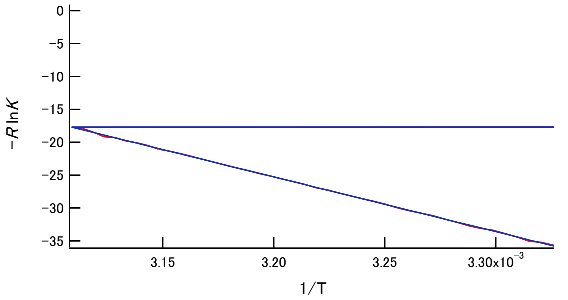

ΔG°_{37vh} = –8.46 kcal/mol

ΔH°_{vh} = –83.25 kcal/mol

ΔS°_{vh} = –241.12 cal/mol·K

$T_{m\,vh}$ = 37.75°C

iv(DNA/RNA) C_t = 3.75 µM

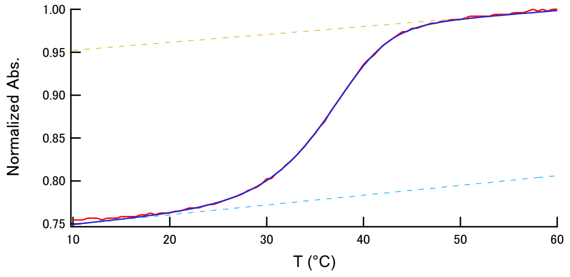

ΔG°_{37} = –8.40 kcal/mol

ΔH° = –87.74 kcal/mol

ΔS° = –255.84 cal/mol·K

T_m = 36.45°C

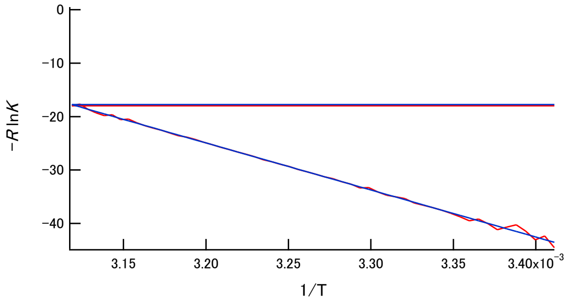

ΔG°_{37vh} = –8.40 kcal/mol

ΔH°_{vh} = –88.09 kcal/mol

ΔS°_{vh} = –256.96 cal/mol·K

$T_{m\,vh}$ = 36.44°C

iv(1/RNA) C_t = 30 µM

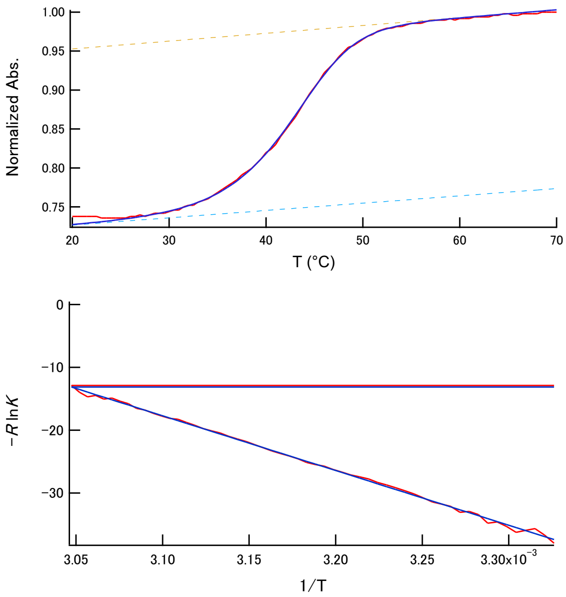

$\Delta G°_{37}$ = –8.84 kcal/mol

$\Delta H°$ = –87.00 kcal/mol

$\Delta S°$ = –252.01 cal/mol·K

T_m = 42.69°C

$\Delta G°_{37vh}$ = –8.85 kcal/mol

$\Delta H°_{vh}$ = –87.18 kcal/mol

$\Delta S°_{vh}$ = –252.55 cal/mol·K

$T_{m\ vh}$ = 42.73°C

iv(1/RNA) C_t = 24 µM

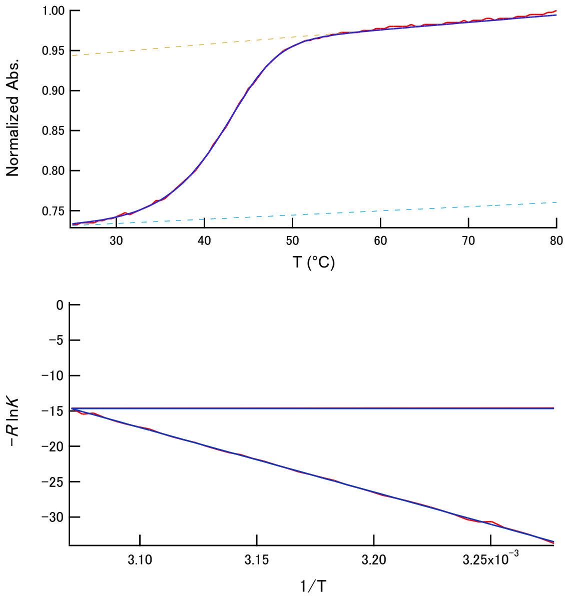

$\Delta G°_{37}$ = –8.89 kcal/mol

$\Delta H°$ = –91.14 kcal/mol

$\Delta S°$ = –265.19 cal/mol·K

T_m = 42.12°C

$\Delta G°_{37vh}$ = –8.89 kcal/mol

$\Delta H°_{vh}$ = –91.18 kcal/mol

$\Delta S°_{vh}$ = –265.32 cal/mol·K

$T_{m\ vh}$ = 42.12°C

iv(1/RNA) C_t = 12 µM

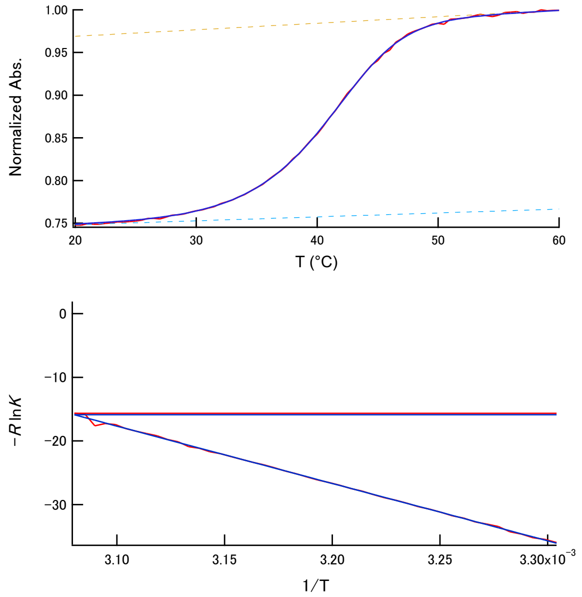

$\Delta G°_{37}$ = –8.96 kcal/mol

$\Delta H°$ = –90.01 kcal/mol

$\Delta S°$ = –261.33 cal/mol·K

T_m = 40.91°C

$\Delta G°_{37vh}$ = –8.96 kcal/mol

$\Delta H°_{vh}$ = –90.16 kcal/mol

$\Delta S°_{vh}$ = –261.82 cal/mol·K

$T_{m\,vh}$ = 40.90°C

iv(1/RNA) C_t = 7.5 µM

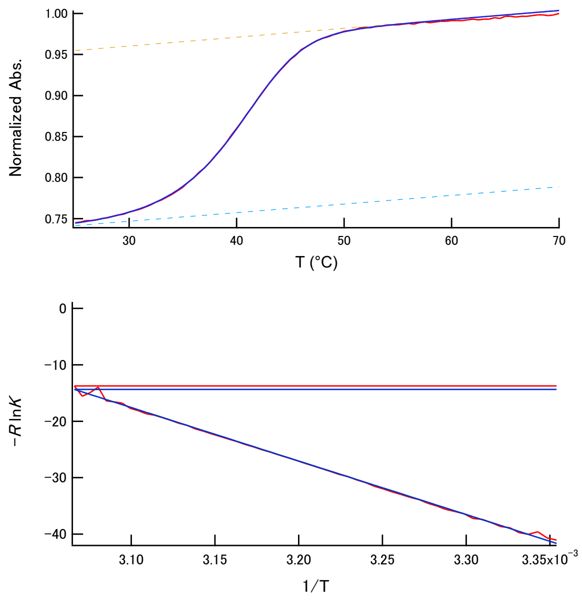

$\Delta G°_{37}$ = –9.12 kcal/mol

$\Delta H°$ = –94.78 kcal/mol

$\Delta S°$ = –276.19 cal/mol·K

T_m = 40.29°C

$\Delta G°_{37vh}$ = –9.10 kcal/mol

$\Delta H°_{vh}$ = –94.81 kcal/mol

$\Delta S°_{vh}$ = –276.35 cal/mol·K

$T_{m\,vh}$ = 40.23°C

iv(1/RNA) C_t = 6 μM

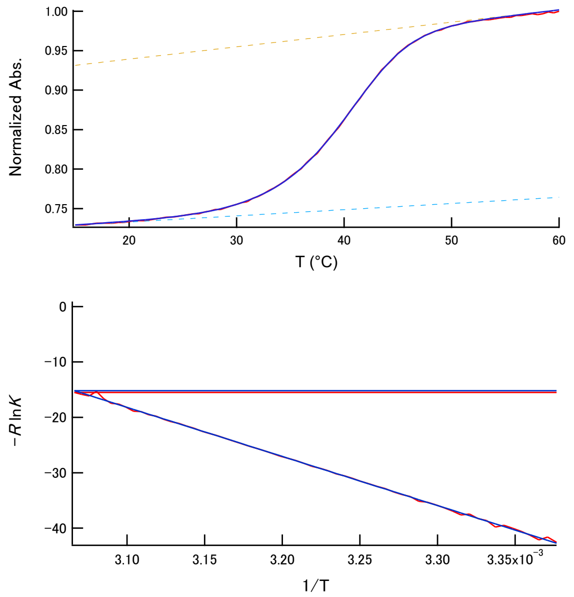

$\Delta G°_{37}$ = −9.06 kcal/mol

$\Delta H°$ = −88.62 kcal/mol

$\Delta S°$ = −256.53 cal/mol·K

T_m = 39.81°C

$\Delta G°_{37vh}$ = −9.06 kcal/mol

$\Delta H°_{vh}$ = −88.66 kcal/mol

$\Delta S°_{vh}$ = −256.64 cal/mol·K

$T_{m\ vh}$ = 39.80°C

iv(2/RNA) C_t = 30 μM

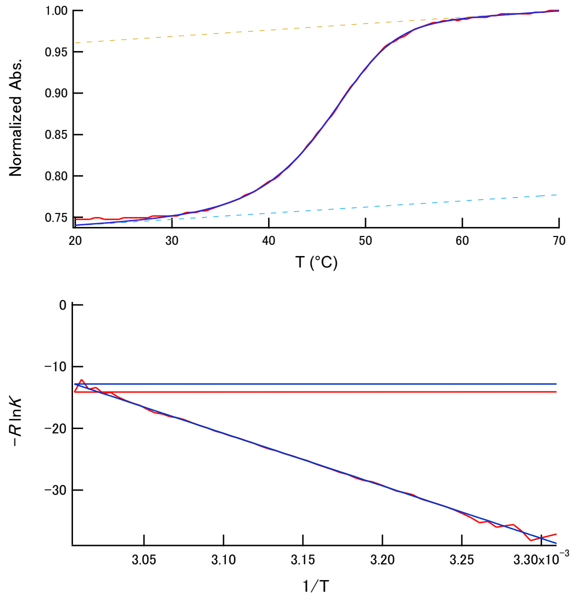

$\Delta G°_{37}$ = −9.74 kcal/mol

$\Delta H°$ = −85.15 kcal/mol

$\Delta S°$ = −243.14 cal/mol·K

T_m = 46.27°C

$\Delta G°_{37vh}$ = −9.73 kcal/mol

$\Delta H°_{vh}$ = −85.10 kcal/mol

$\Delta S°_{vh}$ = −243.01 cal/mol·K

$T_{m\ vh}$ = 46.24°C

iv(2/RNA) C_t = 24 μM

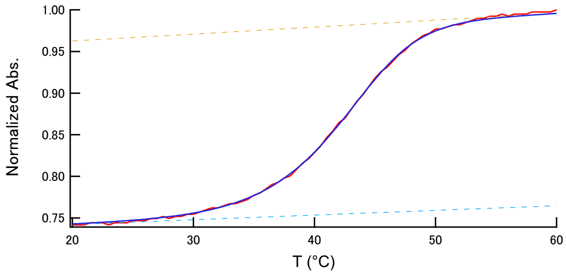

$\Delta G°_{37}$ = –9.76 kcal/mol
$\Delta H°$ = –85.95 kcal/mol
$\Delta S°$ = –245.66 cal/mol·K
T_m = 45.70°C

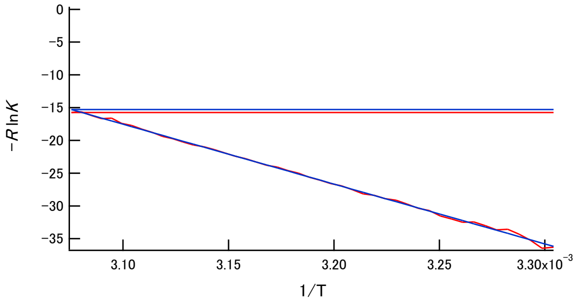

$\Delta G°_{37vh}$ = –9.76 kcal/mol
$\Delta H°_{vh}$ = –85.97 kcal/mol
$\Delta S°_{vh}$ = –245.73 cal/mol·K
$T_{m\ vh}$ = 45.70°C

iv(2/RNA) C_t = 12 μM

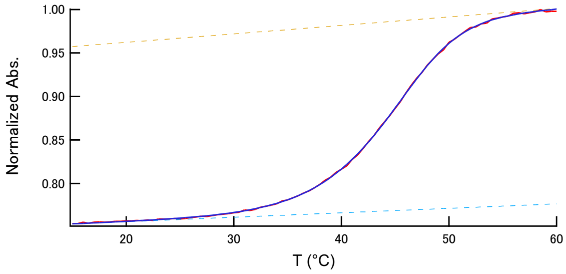

$\Delta G°_{37}$ = –9.87 kcal/mol
$\Delta H°$ = –87.08 kcal/mol
$\Delta S°$ = –248.94 cal/mol·K
T_m = 44.43°C

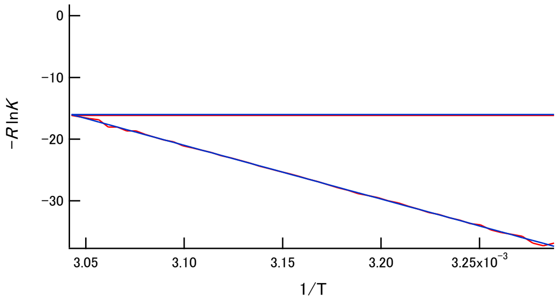

$\Delta G°_{37vh}$ = –9.88 kcal/mol
$\Delta H°_{vh}$ = –87.16 kcal/mol
$\Delta S°_{vh}$ = –249.18 cal/mol·K
$T_{m\ vh}$ = 44.43°C

iv(2/RNA) C_t = 7.5 μM

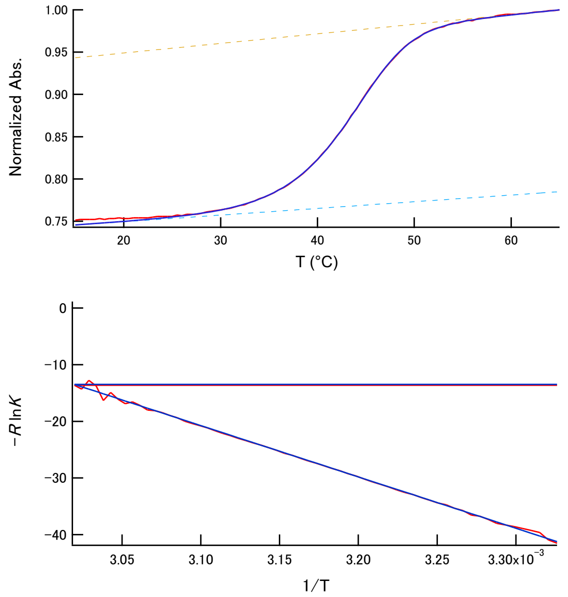

$\Delta G°_{37}$ = –9.93 kcal/mol

$\Delta H°$ = –90.13 kcal/mol

$\Delta S°$ = –258.60 cal/mol·K

T_m = 43.33°C

$\Delta G°_{37vh}$ = –9.93 kcal/mol

$\Delta H°_{vh}$ = –90.55 kcal/mol

$\Delta S°_{vh}$ = –259.96 cal/mol·K

$T_{m\ vh}$ = 43.28°C

iv(2/RNA) C_t = 6 μM

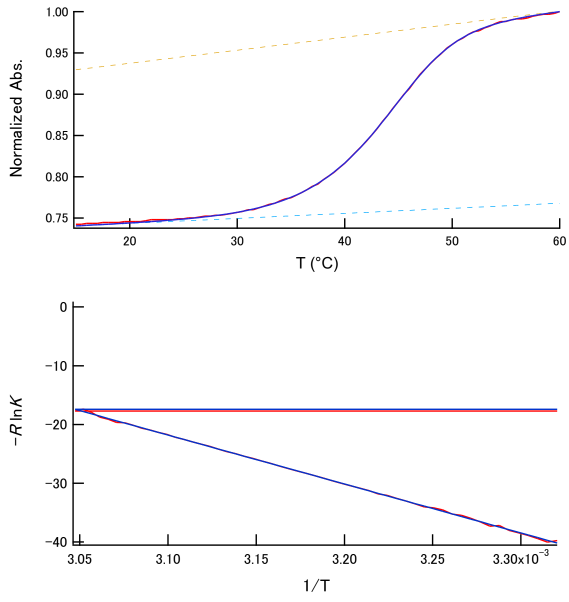

$\Delta G°_{37}$ = –9.97 kcal/mol

$\Delta H°$ = –83.27 kcal/mol

$\Delta S°$ = –236.32 cal/mol·K

T_m = 43.49°C

$\Delta G°_{37vh}$ = –9.97 kcal/mol

$\Delta H°_{vh}$ = –83.30 kcal/mol

$\Delta S°_{vh}$ = –236.43 cal/mol·K

$T_{m\ vh}$ = 43.50°C

iv(3/RNA) C_t = 30 μM

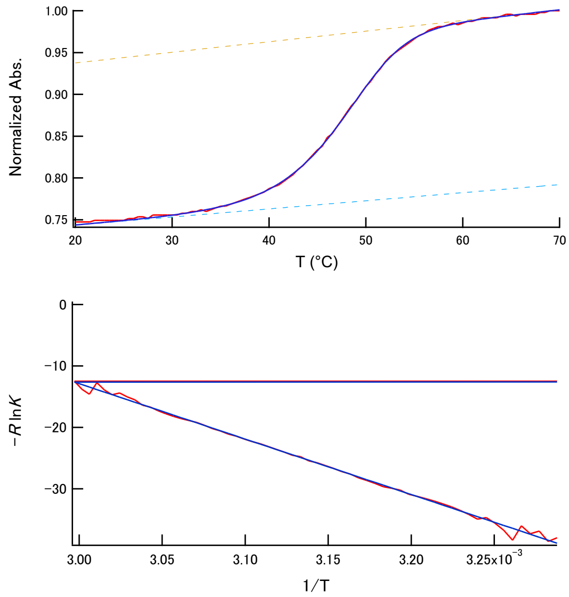

$\Delta G°_{37}$ = –10.27 kcal/mol

$\Delta H°$ = –90.10 kcal/mol

$\Delta S°$ = –257.38 cal/mol·K

T_m = 47.67°C

$\Delta G°_{37vh}$ = –10.27 kcal/mol

$\Delta H°_{vh}$ = –90.19 kcal/mol

$\Delta S°_{vh}$ = –257.70 cal/mol·K

$T_{m\ vh}$ = 47.66°C

iv(3/RNA) C_t = 24 μM

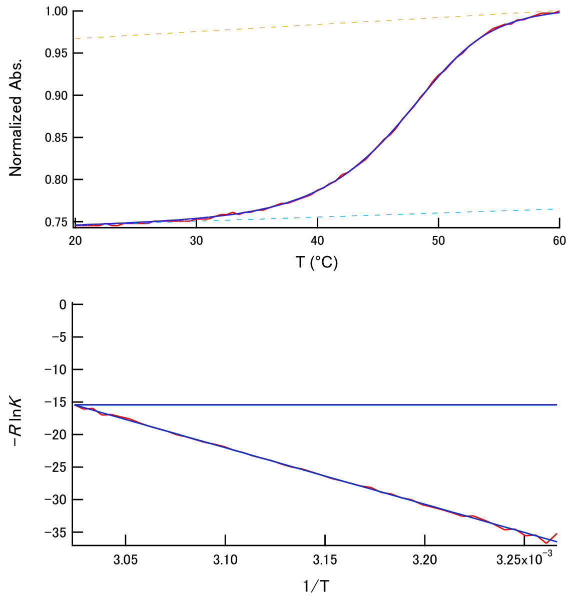

$\Delta G°_{37}$ = –10.17 kcal/mol

$\Delta H°$ = –86.63 kcal/mol

$\Delta S°$ = –246.51 cal/mol·K

T_m = 47.21°C

$\Delta G°_{37vh}$ = –10.18 kcal/mol

$\Delta H°_{vh}$ = –86.91 kcal/mol

$\Delta S°_{vh}$ = –247.40 cal/mol·K

$T_{m\ vh}$ = 47.21°C

iv(3/RNA) C_t = 12 µM

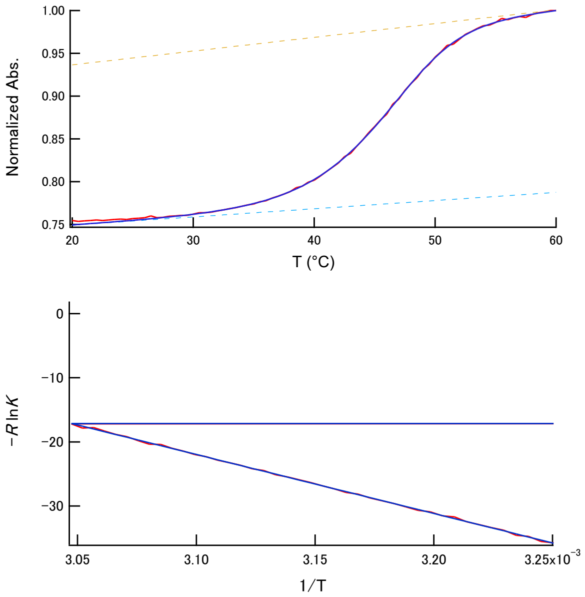

$\Delta G°_{37}$ = –10.35 kcal/mol

$\Delta H°$ = –91.87 kcal/mol

$\Delta S°$ = –262.84 cal/mol·K

T_m = 45.73°C

$\Delta G°_{37vh}$ = –10.35 kcal/mol

$\Delta H°_{vh}$ = –91.90 kcal/mol

$\Delta S°_{vh}$ = –262.93 cal/mol·K

$T_{m\ vh}$ = 45.73°C

iv(3/RNA) C_t = 7.5 µM

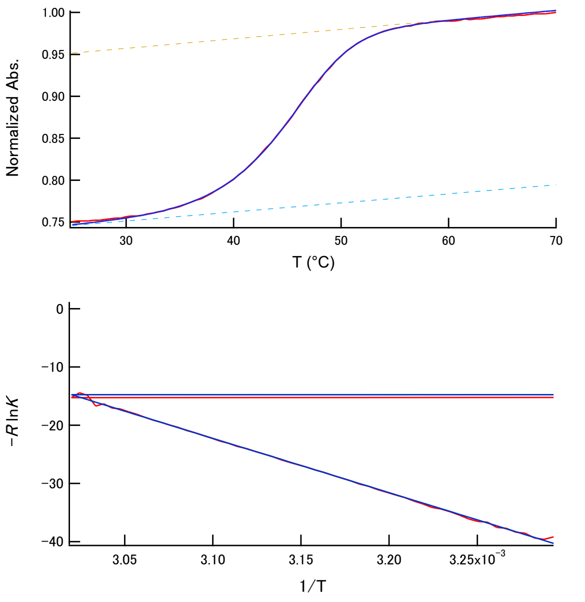

$\Delta G°_{37}$ = –10.51 kcal/mol

$\Delta H°$ = –93.59 kcal/mol

$\Delta S°$ = –267.87 cal/mol·K

T_m = 45.11°C

$\Delta G°_{37vh}$ = –10.50 kcal/mol

$\Delta H°_{vh}$ = –93.34 kcal/mol

$\Delta S°_{vh}$ = –267.12 cal/mol·K

$T_{m\ vh}$ = 45.08°C

iv(3/RNA) C_t = 6 µM

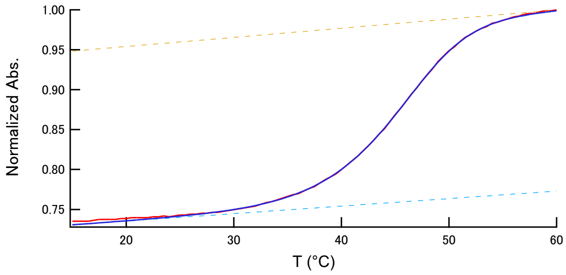

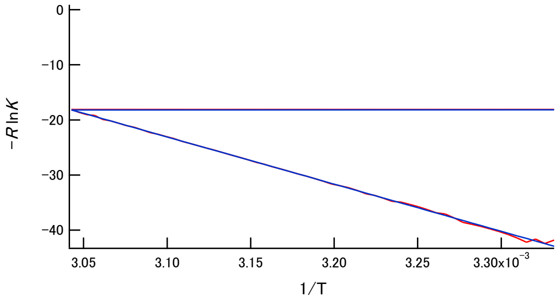

ΔG°_{37} = –10.46 kcal/mol

ΔH° = –85.65 kcal/mol

ΔS° = –242.43 cal/mol·K

T_m = 45.17°C

ΔG°_{37vh} = –10.46 kcal/mol

ΔH°_{vh} = –85.67 kcal/mol

ΔS°_{vh} = –242.48 cal/mol·K

$T_{m\ vh}$ = 45.17°C

iv(4/RNA) C_t = 30 µM

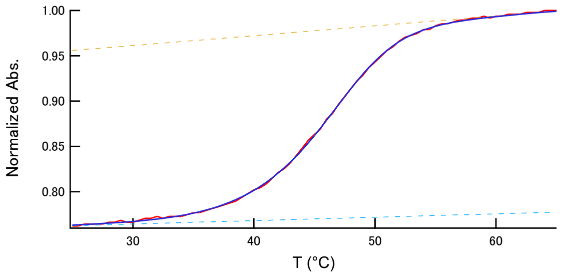

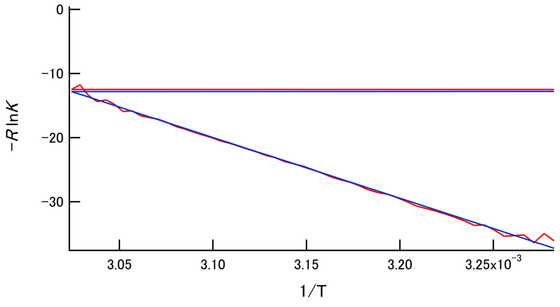

ΔG°_{37} = –9.88 kcal/mol

ΔH° = –94.97 kcal/mol

ΔS° = –274.35 cal/mol·K

T_m = 45.74°C

ΔG°_{37vh} = –9.85 kcal/mol

ΔH°_{vh} = –94.75 kcal/mol

ΔS°_{vh} = –273.76 cal/mol·K

$T_{m\ vh}$ = 45.66°C

iv(4/RNA) C_t = 24 μM

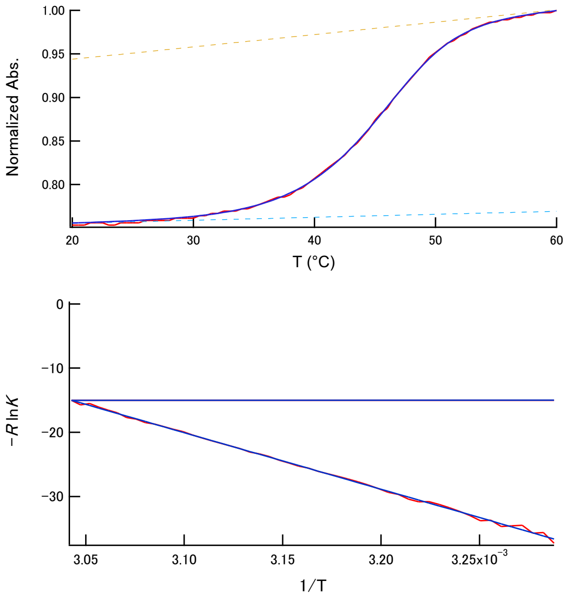

ΔG°_{37} = –9.61 kcal/mol

ΔH° = –88.07 kcal/mol

ΔS° = –252.95 cal/mol·K

T_m = 44.96°C

ΔG°_{37vh} = –9.62 kcal/mol

ΔH°_{vh} = –88.16 kcal/mol

ΔS°_{vh} = –253.23 cal/mol·K

$T_{m\ vh}$ = 44.96°C

iv(4/RNA) C_t = 12 μM

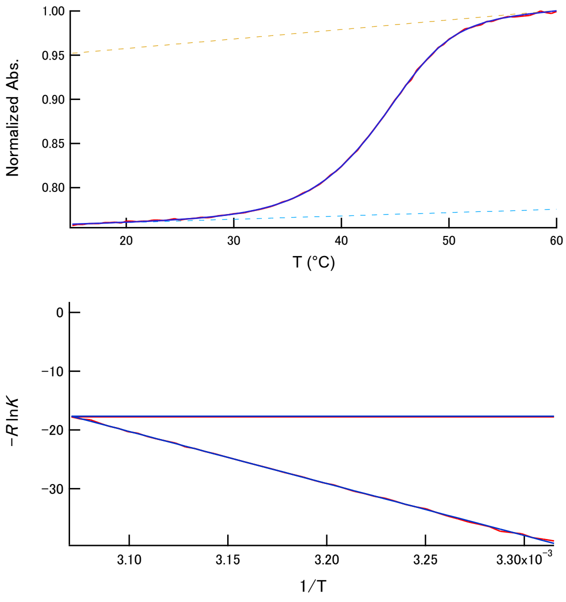

ΔG°_{37} = –9.70 kcal/mol

ΔH° = –88.75 kcal/mol

ΔS° = –254.89 cal/mol·K

T_m = 43.65°C

ΔG°_{37vh} = –9.70 kcal/mol

ΔH°_{vh} = –88.61 kcal/mol

ΔS°_{vh} = –254.45 cal/mol·K

$T_{m\ vh}$ = 43.65°C

iv(4/RNA) C_t = 7.5 μM

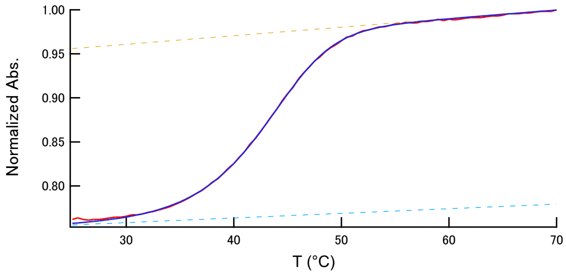

$\Delta G°_{37}$ = –9.85 kcal/mol

$\Delta H°$ = –91.49 kcal/mol

$\Delta S°$ = –263.25 cal/mol·K

T_m = 42.94°C

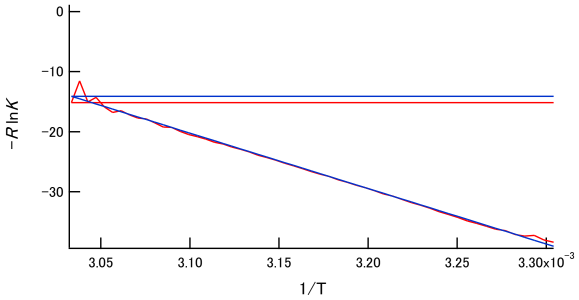

$\Delta G°_{37vh}$ = –9.83 kcal/mol

$\Delta H°_{vh}$ = –92.30 kcal/mol

$\Delta S°_{vh}$ = –265.89 cal/mol·K

$T_{m\ vh}$ = 42.85°C

iv(4/RNA) C_t = 6 μM

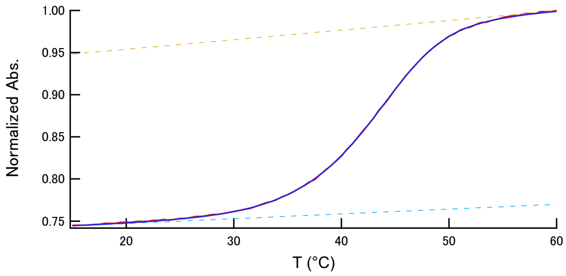

$\Delta G°_{37}$ = –9.83 kcal/mol

$\Delta H°$ = –84.60 kcal/mol

$\Delta S°$ = –241.09 cal/mol·K

T_m = 42.84°C

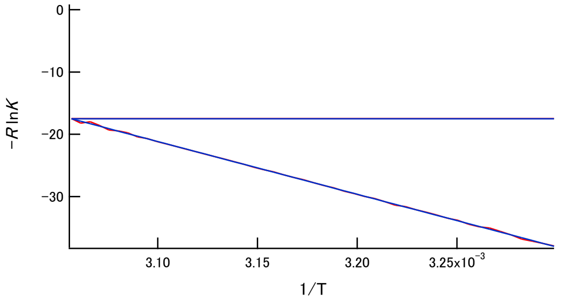

$\Delta G°_{37vh}$ = –9.83 kcal/mol

$\Delta H°_{vh}$ = –84.64 kcal/mol

$\Delta S°_{vh}$ = –241.20 cal/mol·K

$T_{m\ vh}$ = 42.84°C

iv(5/RNA) C_t = 30 μM

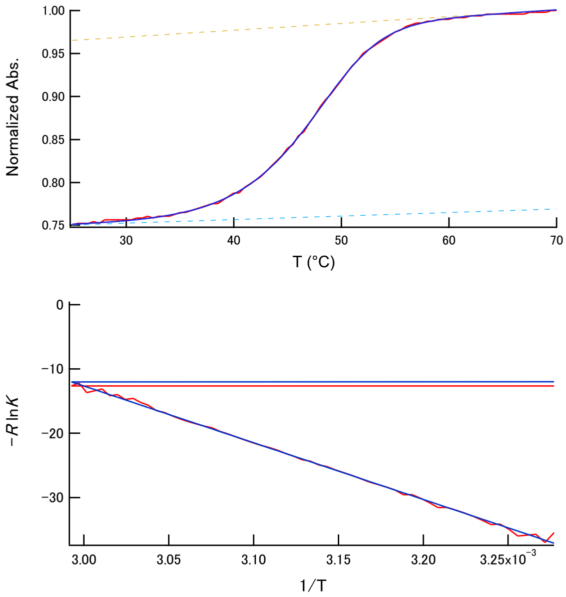

ΔG°_{37} = –10.08 kcal/mol

ΔH° = –88.70 kcal/mol

ΔS° = –253.51 cal/mol·K

T_m = 47.13°C

ΔG°_{37vh} = –10.06 kcal/mol

ΔH°_{vh} = –88.23 kcal/mol

ΔS°_{vh} = –252.04 cal/mol·K

$T_{m\ vh}$ = 47.13°C

iv(5/RNA) C_t = 24 μM

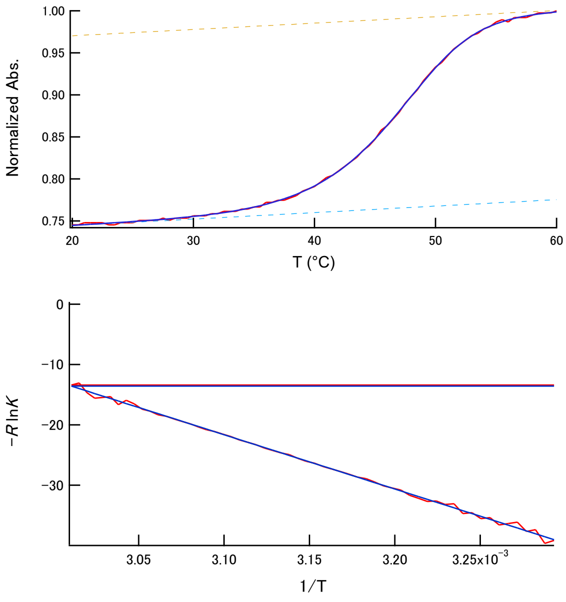

ΔG°_{37} = –10.17 kcal/mol

ΔH° = –89.63 kcal/mol

ΔS° = –256.20 cal/mol·K

T_m = 46.84°C

ΔG°_{37vh} = –10.17 kcal/mol

ΔH°_{vh} = –90.04 kcal/mol

ΔS°_{vh} = –257.49 cal/mol·K

$T_{m\ vh}$ = 46.83°C

iv(5/RNA) C_t = 12 μM

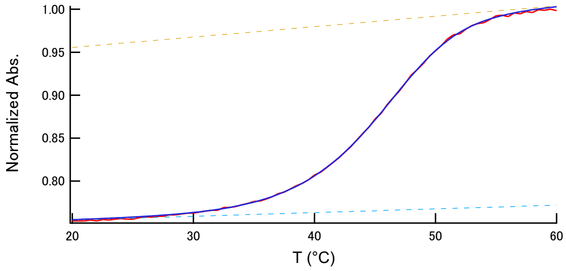

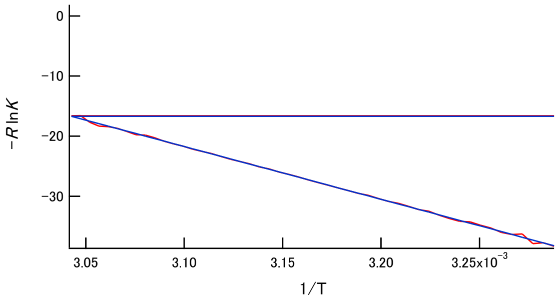

$\Delta G°_{37}$ = –10.12 kcal/mol
$\Delta H°$ = –87.70 kcal/mol
$\Delta S°$ = –250.14 cal/mol·K
T_m = 45.28°C

$\Delta G°_{37vh}$ = –10.12 kcal/mol
$\Delta H°_{vh}$ = –87.88 kcal/mol
$\Delta S°_{vh}$ = –250.70 cal/mol·K
$T_{m\ vh}$ = 45.28°C

iv(5/RNA) C_t = 7.5 μM

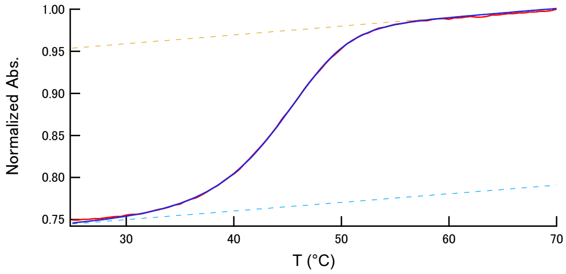

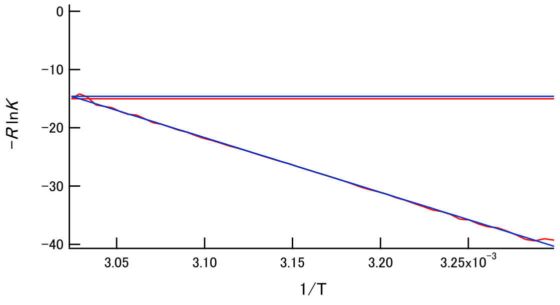

$\Delta G°_{37}$ = –10.36 kcal/mol
$\Delta H°$ = –93.83 kcal/mol
$\Delta S°$ = –269.13 cal/mol·K
T_m = 44.55°C

$\Delta G°_{37vh}$ = –10.34 kcal/mol
$\Delta H°_{vh}$ = –93.85 kcal/mol
$\Delta S°_{vh}$ = –269.24 cal/mol·K
$T_{m\ vh}$ = 44.51°C

iv(5/RNA) C_t = 6 μM

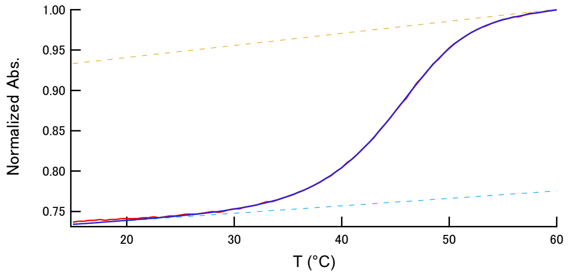

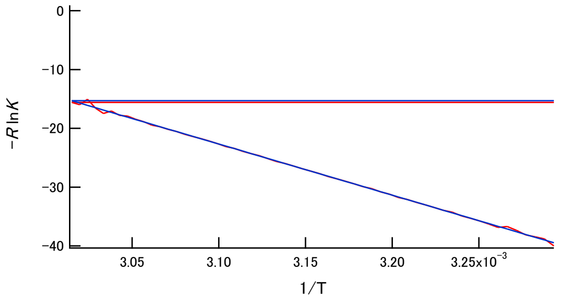

$\Delta G°_{37}$ = –10.37 kcal/mol

$\Delta H°$ = –86.65 kcal/mol

$\Delta S°$ = –245.93 cal/mol·K

T_m = 44.73°C

$\Delta G°_{37vh}$ = –10.37 kcal/mol

$\Delta H°_{vh}$ = –86.79 kcal/mol

$\Delta S°_{vh}$ = –246.39 cal/mol·K

$T_{m\ vh}$ = 44.72°C

iv(6/RNA) C_t = 30 μM

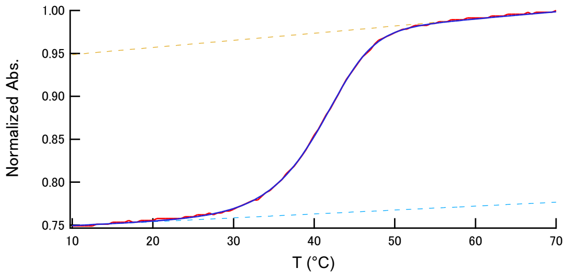

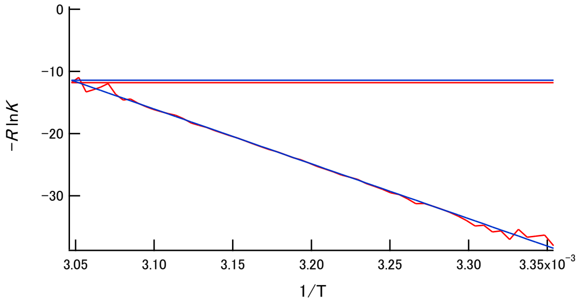

$\Delta G°_{37}$ = –8.39 kcal/mol

$\Delta H°$ = –88.72 kcal/mol

$\Delta S°$ = –258.98 cal/mol·K

T_m = 40.97°C

$\Delta G°_{37vh}$ = –8.37 kcal/mol

$\Delta H°_{vh}$ = –88.20 kcal/mol

$\Delta S°_{vh}$ = –257.38 cal/mol·K

$T_{m\ vh}$ = 40.93°C

iv(6/RNA) C_t = 24 μM

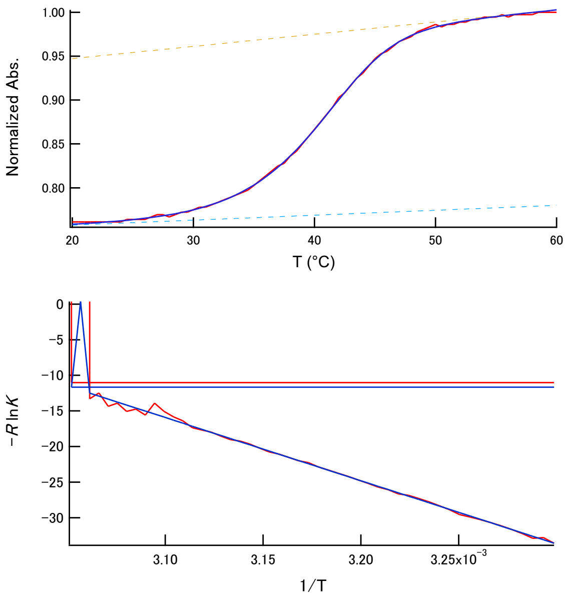

$\Delta G°_{37}$ = –8.36 kcal/mol

$\Delta H°$ = –89.03 kcal/mol

$\Delta S°$ = –260.11 cal/mol·K

T_m = 40.36°C

$\Delta G°_{37vh}$ = –8.36 kcal/mol

$\Delta H°_{vh}$ = –88.82 kcal/mol

$\Delta S°_{vh}$ = –259.40 cal/mol·K

$T_{m\ vh}$ = 40.36°C

iv(6/RNA) C_t = 12 μM

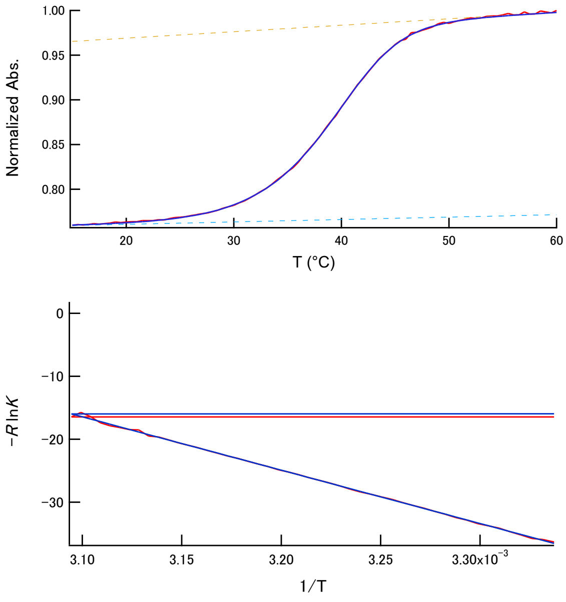

$\Delta G°_{37}$ = –8.37 kcal/mol

$\Delta H°$ = –84.66 kcal/mol

$\Delta S°$ = –246.01 cal/mol·K

T_m = 38.95°C

$\Delta G°_{37vh}$ = –8.36 kcal/mol

$\Delta H°_{vh}$ = –84.74 kcal/mol

$\Delta S°_{vh}$ = –246.26 cal/mol·K

$T_{m\ vh}$ = 38.94°C

iv(6/RNA) C_t = 7.5 μM

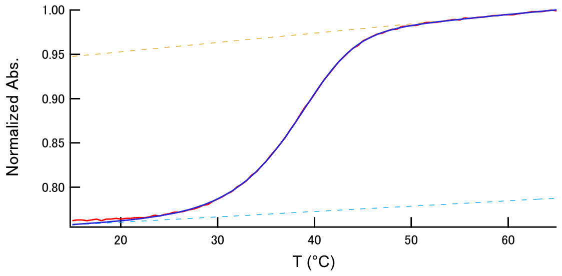

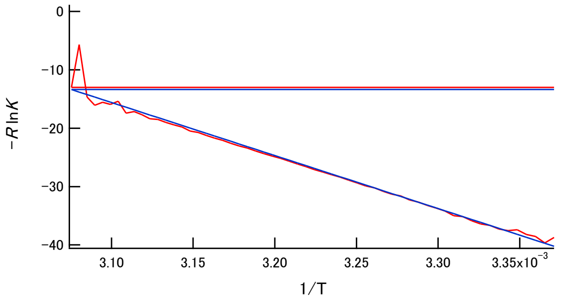

$\Delta G°_{37}$ = –8.39 kcal/mol

$\Delta H°$ = –88.39 kcal/mol

$\Delta S°$ = –257.92 cal/mol·K

T_m = 37.94°C

$\Delta G°_{37vh}$ = –8.34 kcal/mol

$\Delta H°_{vh}$ = –90.90 kcal/mol

$\Delta S°_{vh}$ = –266.18 cal/mol·K

$T_{m\ vh}$ = 37.73°C

iv(6/RNA) C_t = 6 μM

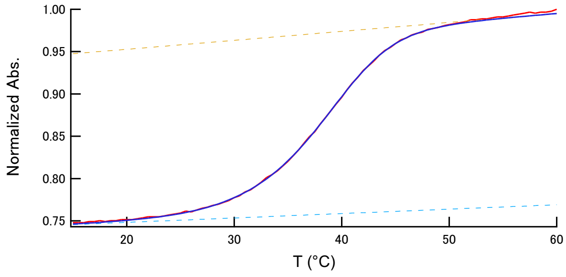

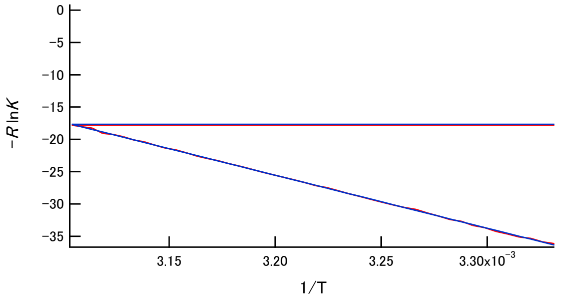

$\Delta G°_{37}$ = –8.54 kcal/mol

$\Delta H°$ = –82.10 kcal/mol

$\Delta S°$ = –237.18 cal/mol·K

T_m = 38.04°C

$\Delta G°_{37vh}$ = –8.54 kcal/mol

$\Delta H°_{vh}$ = –82.08 kcal/mol

$\Delta S°_{vh}$ = –237.11 cal/mol·K

$T_{m\ vh}$ = 38.04°C

Figure S1. Melting curves (upper panels) and van't Hoff plots (lower panels) of the duplexes at varying concentrations. For each sample, representative data from up to quadruplicated experiments are shown. Actual melting curves and results of the curve fitting are shown in red and blue, respectively. In the conversion of the two melting curves to the 1/T vs. –Rln*K* plots, horizontal lines starting from the y-intercept appeared probably due to a bug of the program. The thermodynamic parameters obtained by the curve fitting and the van't Hoff plots of each data shown in the left panel are listed.

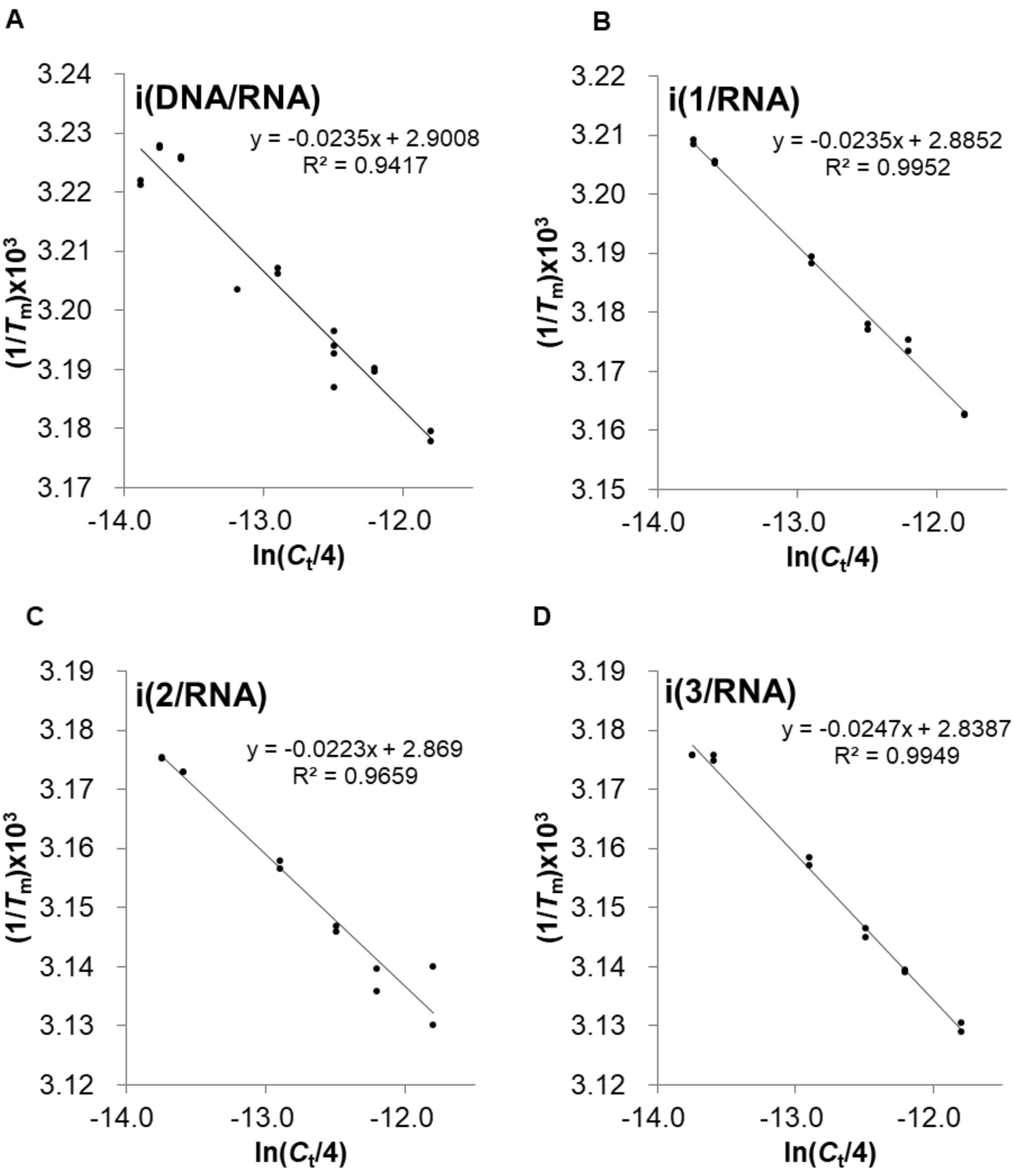
A
i(DNA/RNA)
y = -0.0235x + 2.9008
R² = 0.9417
3.24
3.23
3.22
3.21
3.20
3.19
3.18
3.17
(1/Tm)x10³
-14.0
-13.0
-12.0
ln(Ct/4)
B
i(1/RNA)
y = -0.0235x + 2.8852
R² = 0.9952
3.22
3.21
3.20
3.19
3.18
3.17
3.16
3.15
(1/Tm)x10³
-14.0
-13.0
-12.0
ln(Ct/4)
C
i(2/RNA)
y = -0.0223x + 2.869
R² = 0.9659
3.19
3.18
3.17
3.16
3.15
3.14
3.13
3.12
(1/Tm)x10³
-14.0
-13.0
-12.0
ln(Ct/4)
D
i(3/RNA)
y = -0.0247x + 2.8387
R² = 0.9949
3.19
3.18
3.17
3.16
3.15
3.14
3.13
3.12
(1/Tm)x10³
-14.0
-13.0
-12.0
ln(Ct/4)

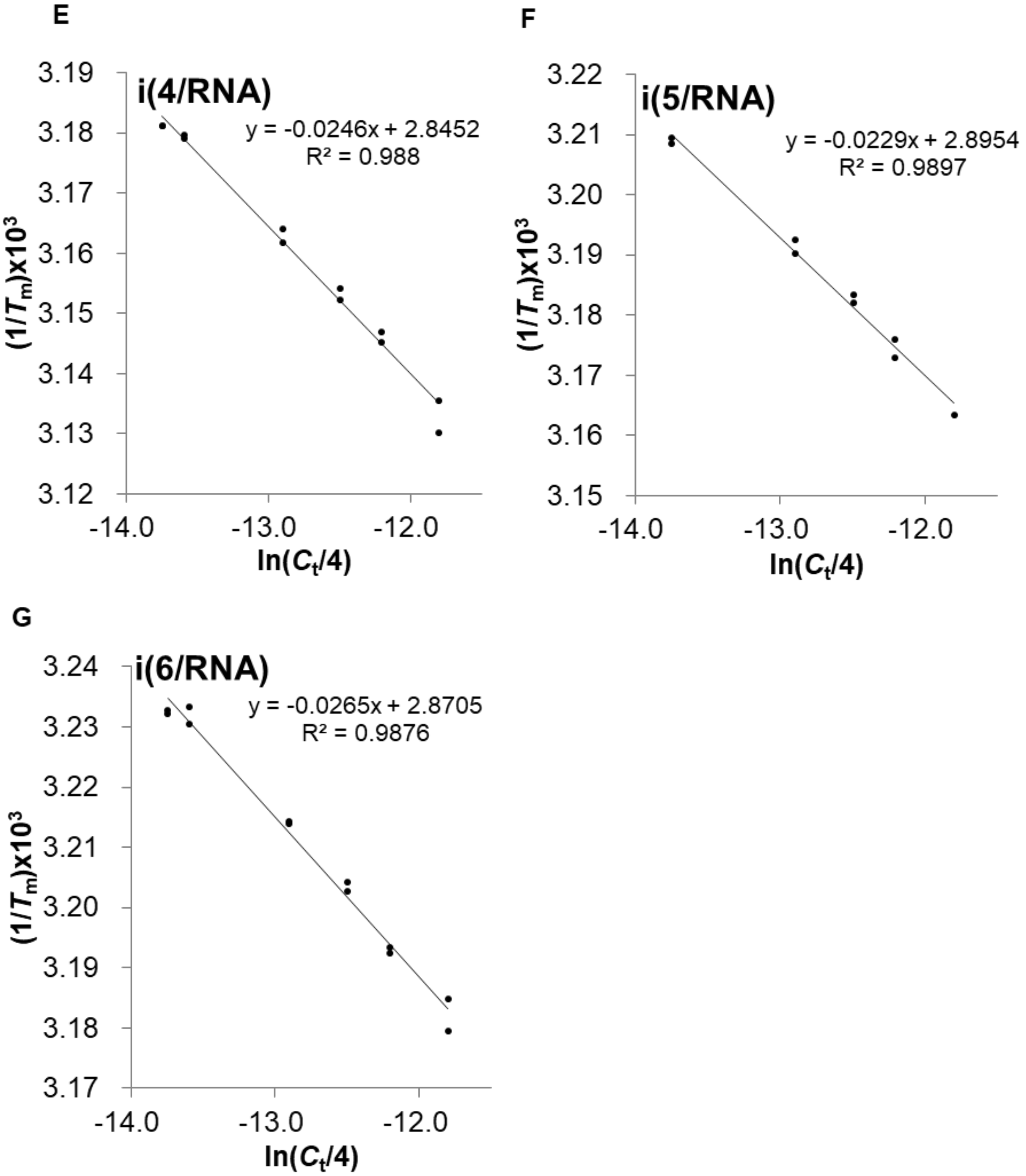

Figure S2. Linear least squares fitting (black lines) of van't Hoff plots showing inverse melting point temperatures ($1/T_m$) obtained by measurements of melting curves at different total oligonucleotide concentrations (C_t) of DNA/RNA duplexes (with sequence i). The duplex identities and R^2 values are also shown for each plot. (A), (D) and (E) are reproduced from our previous study [14].

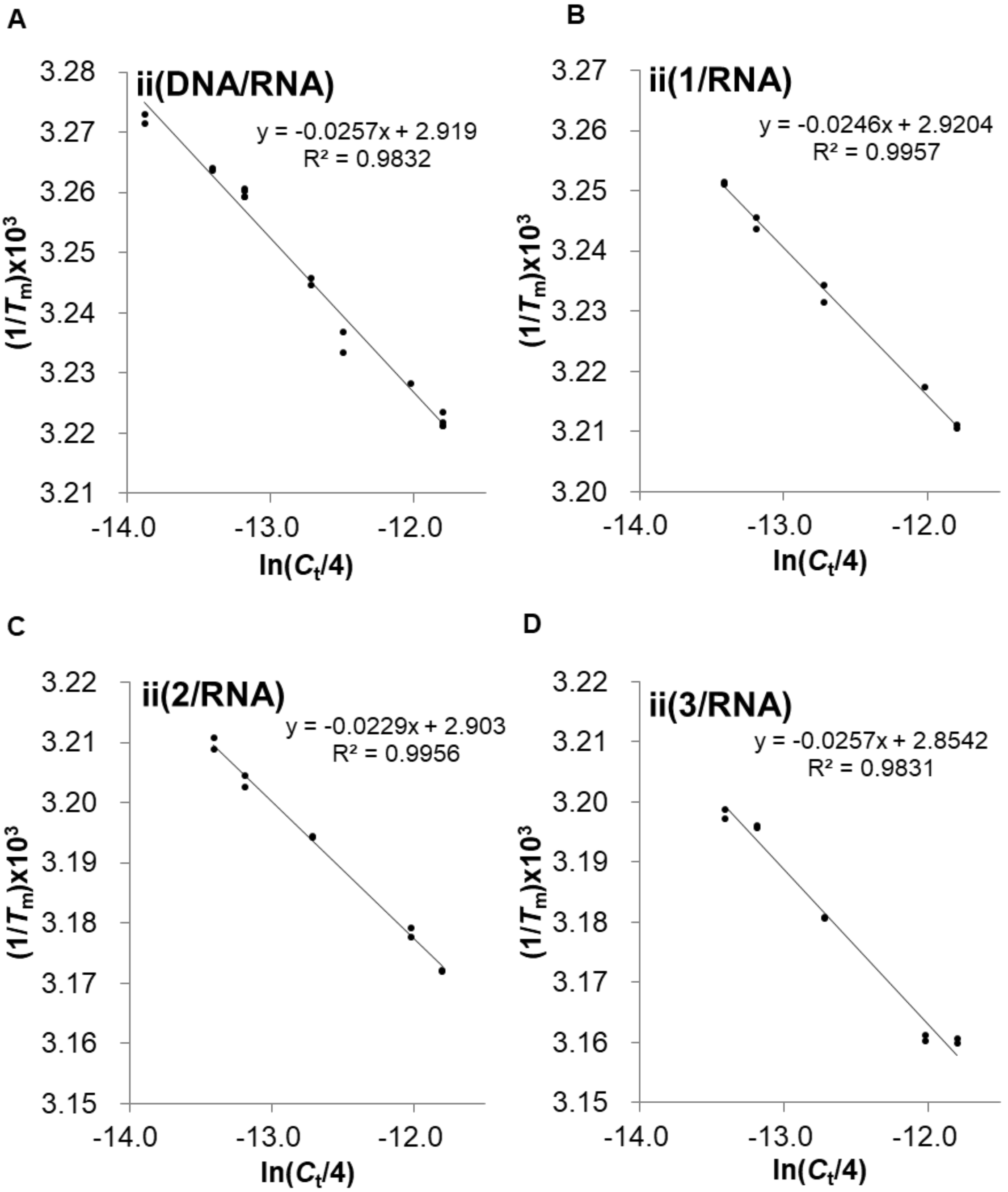
A
ii(DNA/RNA)
y = -0.0257x + 2.919
R² = 0.9832
(1/Tm)x10³
ln(Ct/4)
3.28
3.27
3.26
3.25
3.24
3.23
3.22
3.21
-14.0
-13.0
-12.0
B
ii(1/RNA)
y = -0.0246x + 2.9204
R² = 0.9957
(1/Tm)x10³
ln(Ct/4)
3.27
3.26
3.25
3.24
3.23
3.22
3.21
3.20
-14.0
-13.0
-12.0
C
ii(2/RNA)
y = -0.0229x + 2.903
R² = 0.9956
(1/Tm)x10³
ln(Ct/4)
3.22
3.21
3.20
3.19
3.18
3.17
3.16
3.15
-14.0
-13.0
-12.0
D
ii(3/RNA)
y = -0.0257x + 2.8542
R² = 0.9831
(1/Tm)x10³
ln(Ct/4)
3.22
3.21
3.20
3.19
3.18
3.17
3.16
3.15
-14.0
-13.0
-12.0

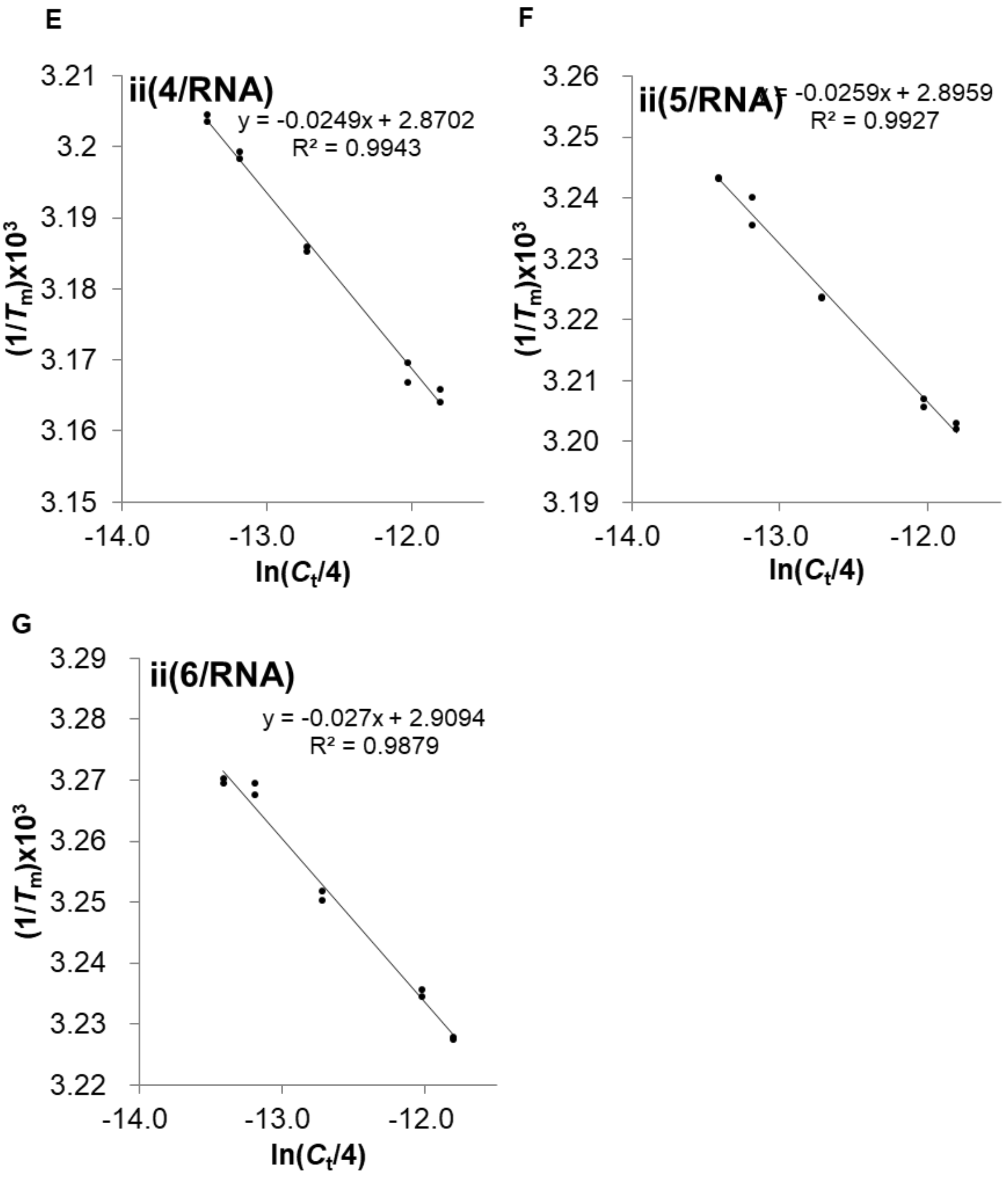

Figure S3. Linear least squares fitting (black lines) of van't Hoff plots showing inverse melting point temperatures ($1/T_m$) obtained by measurements of melting curves at different total oligonucleotide concentrations (C_t) of DNA/RNA duplexes (with sequence ii). The duplex identities and R^2 values are also shown for each plot. (A), (D) and (E) are reproduced from our previous study [14].

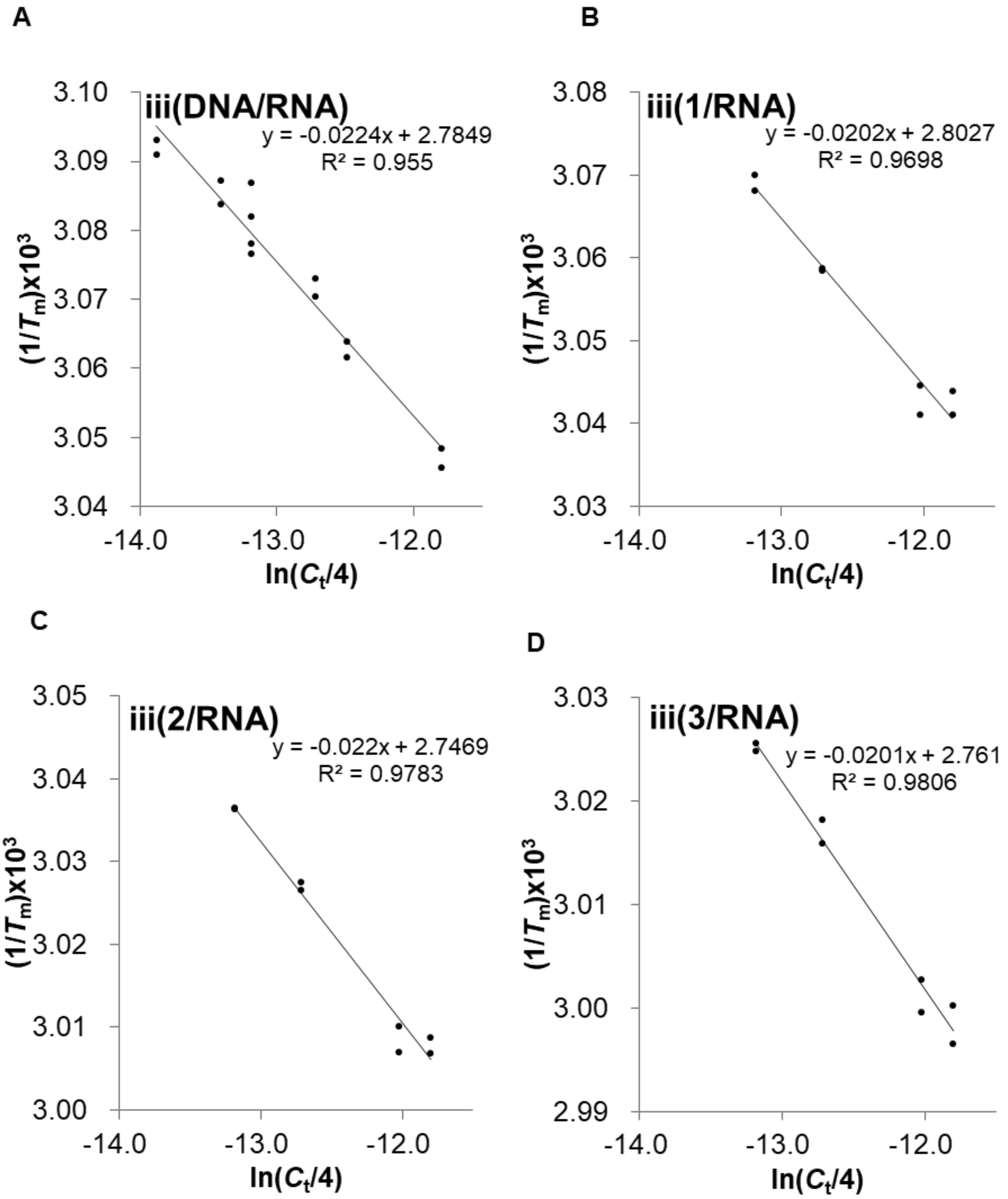

A
iii(DNA/RNA)
y = -0.0224x + 2.7849
R² = 0.955
(1/Tm)x10³
ln(Ct/4)
3.10
3.09
3.08
3.07
3.06
3.05
3.04
-14.0
-13.0
-12.0
B
iii(1/RNA)
y = -0.0202x + 2.8027
R² = 0.9698
(1/Tm)x10³
ln(Ct/4)
3.08
3.07
3.06
3.05
3.04
3.03
-14.0
-13.0
-12.0
C
iii(2/RNA)
y = -0.022x + 2.7469
R² = 0.9783
(1/Tm)x10³
ln(Ct/4)
3.05
3.04
3.03
3.02
3.01
3.00
-14.0
-13.0
-12.0
D
iii(3/RNA)
y = -0.0201x + 2.761
R² = 0.9806
(1/Tm)x10³
ln(Ct/4)
3.03
3.02
3.01
3.00
2.99
-14.0
-13.0
-12.0

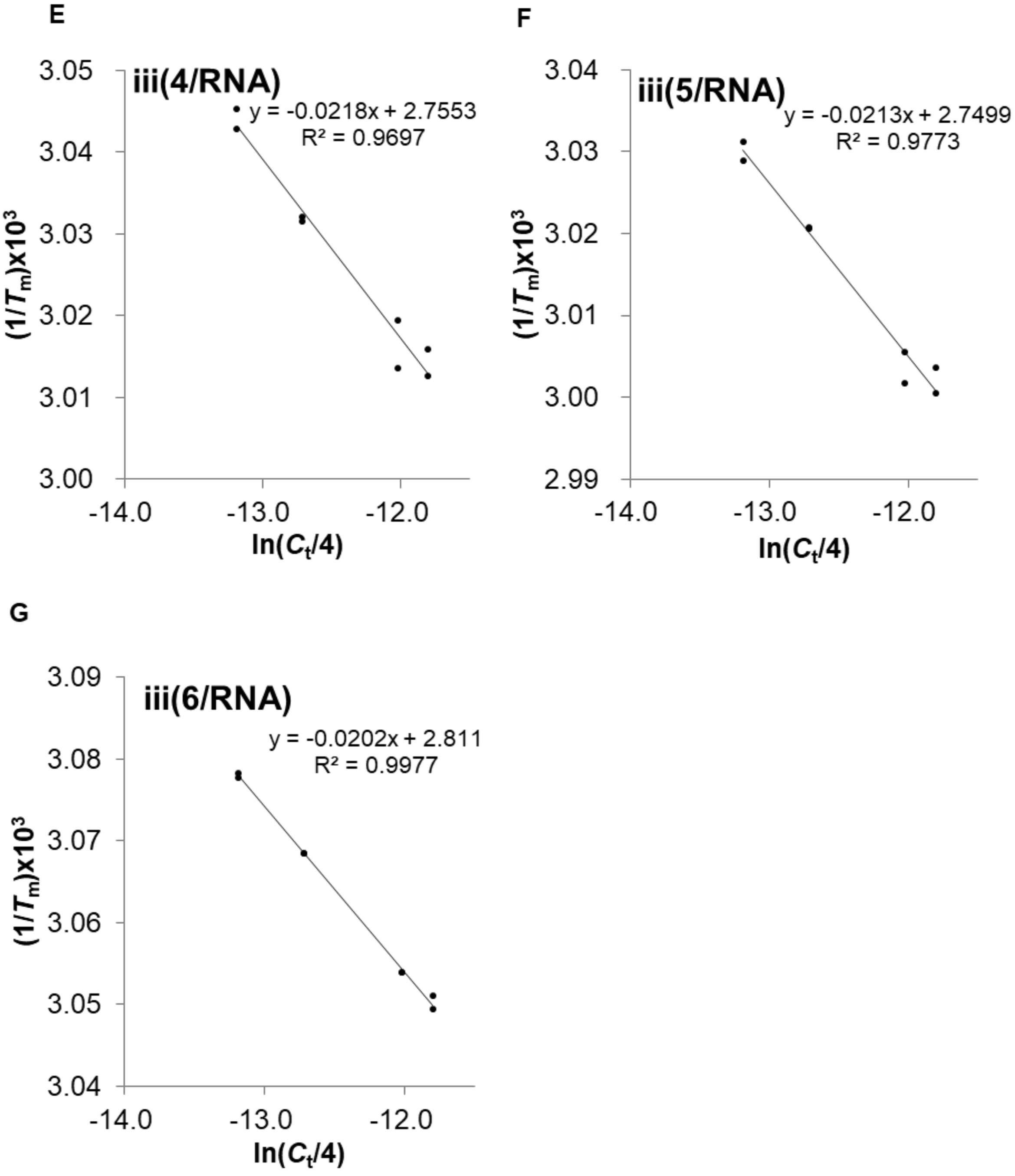

Figure S4. Linear least squares fitting (black lines) of van't Hoff plots showing inverse melting point temperatures ($1/T_m$) obtained by measurements of melting curves at different total oligonucleotide concentrations (C_t) of DNA/RNA duplexes (with sequence iii). The duplex identities and R^2 values are also shown for each plot. (A), (B), (D), (E) and (F) are reproduced from our previous study [14].

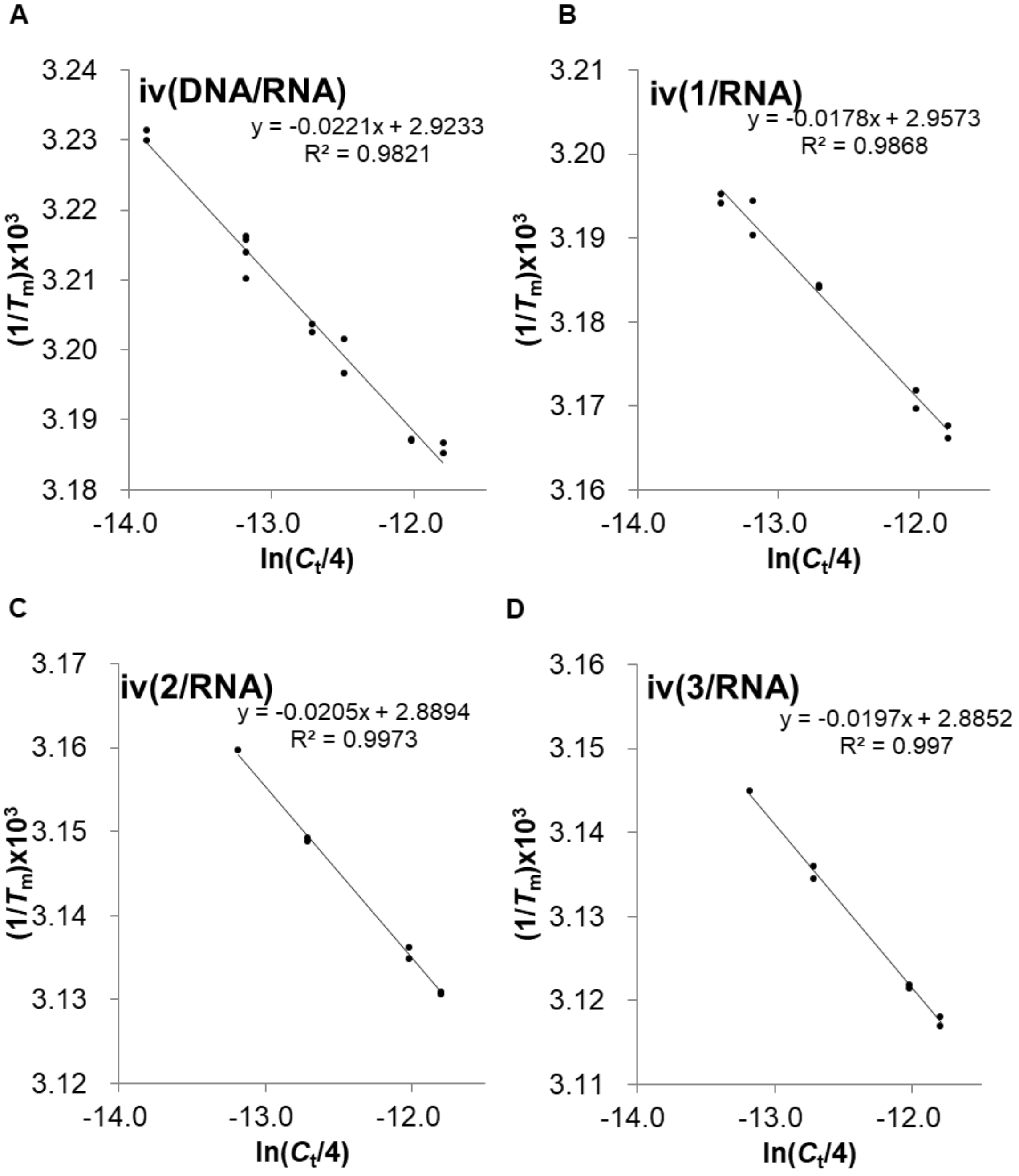

A
iv(DNA/RNA)
y = -0.0221x + 2.9233
R² = 0.9821
(1/Tm)x10³
3.24
3.23
3.22
3.21
3.20
3.19
3.18
-14.0
-13.0
-12.0
ln(Ct/4)
B
iv(1/RNA)
y = -0.0178x + 2.9573
R² = 0.9868
(1/Tm)x10³
3.21
3.20
3.19
3.18
3.17
3.16
-14.0
-13.0
-12.0
ln(Ct/4)
C
iv(2/RNA)
y = -0.0205x + 2.8894
R² = 0.9973
(1/Tm)x10³
3.17
3.16
3.15
3.14
3.13
3.12
-14.0
-13.0
-12.0
ln(Ct/4)
D
iv(3/RNA)
y = -0.0197x + 2.8852
R² = 0.997
(1/Tm)x10³
3.16
3.15
3.14
3.13
3.12
3.11
-14.0
-13.0
-12.0
ln(Ct/4)

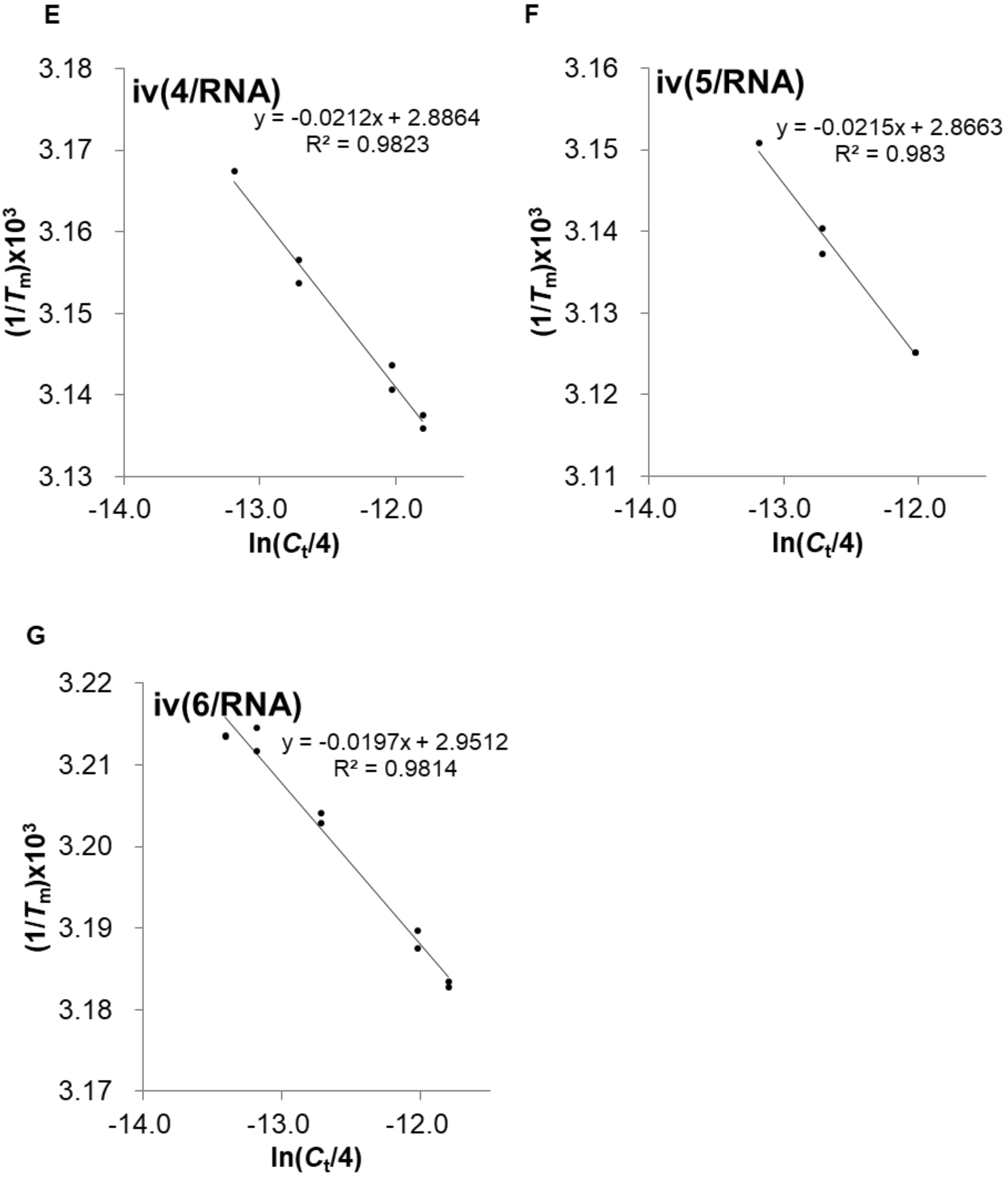

Figure S5. Linear least squares fitting (black lines) of van't Hoff plots showing inverse melting point temperatures ($1/T_m$) obtained by measurements of melting curves at different total oligonucleotide concentrations (C_t) of DNA/RNA duplexes (with sequence iv). The duplex identities and R^2 values are also shown foreach plot. (A), (B), (D), (E) and (F) are reproduced from our previous study [14].
